# Methylation dynamics during the maternal-to-zygotic genome transition in dioecious species

**DOI:** 10.1371/journal.pone.0200028

**Published:** 2018-07-10

**Authors:** Willian T. A. F. Silva

**Affiliations:** Department of Evolutionary Biology, Uppsala University, Norbyvägen 18D, 753 10 Uppsala, Sweden; University of Iceland, ICELAND

## Abstract

The starting point of a new generation in sexually reproducing species is fertilization. In many species, fertilization is followed by cell divisions controlled primarily by maternal transcripts, with little to no zygotic transcription. The activation of the zygotic genome (ZGA) is part of a process called maternal-to-zygotic transition (MZT), during which transcripts from the zygotic genome take control of development, setting the conditions for cellular specialization. While we know that epigenetic processes (e.g. methylation) are involved in the MZT, their roles and interplay in the transition are largely unknown. I developed a model and used simulations to elucidate the interaction between possible epigenetic processes, namely methylation processes, involved in the MZT. The model focuses on the dynamics of global methylation levels and how these interact with factors such as a parental repressor and the nucleocytoplasmic ratio to trigger the ZGA, followed by development from fertilization to adulthood. In addition, I included transgenerational effects transmitted to the zygote from both parents through their gametes to show that these may set the stage for plastic developmental processes. I demonstrate that the rates of maintenance methylation and demethylation, which are important for the achievement of the final methylation levels of an individual, exhibit a certain level of flexibility in terms of parameter values. I find that high final methylation levels require more restricted combinations of parameter values. The model is discussed in the context of the current empirical knowledge and provide suggestions for directions of future empirical and theoretical studies.

## Introduction

How can the fusion of two highly specialized cells (sperm and egg) generate a state of totipotency, from which all other cell types descend? It is known that the genome of a cell at any time point is only partially activated, with the majority of genes being tightly regulated by highly specialized mechanisms [1]. Given the vast number of possible combinations of activated and repressed genes in a genome, an immense number of potentially different cell types can be generated by a single genome. The expression of a gene is generally known to be controlled at multiple molecular levels by pre-transcriptional and post-transcriptional mechanisms. While mechanisms such as mRNA targeting by small RNAs [2] and protein modification [3] regulate gene expression at the post-transcriptional level, epigenetic mechanisms such as DNA methylation [4] and chromatin modifications [5] are involved in the pre-transcriptional control of gene expression. These mechanisms are particularly important during the initial stages of development right after fertilization, when the parental genomes need to function together and synchronize their genes in order to create a viable individual [6]. The strong evidence provided in recent studies highlighting the importance of non-genetic information inherited from both the mother [7] and the father [8] raises further questions about the dynamics involved during those crucial early developmental steps and throughout life into adulthood. Based on the current empirical knowledge, I propose a model for the dynamics of the intra- and transgenerational epigenetic remodeling of global methylation levels in particular that occur during the transition from a totipotent zygote into the highly specialized epigenetic patterns of the gametes in the adult germline.

The transition from parentally controlled gene expression to zygotic gene expression (maternal-to-zygotic transition, MZT) is referred to as “Zygotic Genome Activation” (ZGA) and takes place at a very specific time point during early embryo development [9]. Given that the eggs are the providers of the zygotic cytoplasm and the parental genomes show little to no transcription, it is believed that the very early stages of development are largely controlled by maternally inherited factors, like RNAs and proteins [10–12]. The relative importance of paternally contributed RNAs transferred through sperm is still poorly understood. Detailed analyses of the transcriptome and the methylome throughout different developmental stages have improved our understanding of the changes in gene expression patterns during early development [13, 14]. These observations revealed the timing of activation of the zygotic genome and helped identifying the potential factors involved in the process. One such factor is the nucleocytoplasmic ratio, which changes as development proceeds due to the conservation of the total volume of the embryo while the number of cells increases exponentially. The nucleocytoplasmic ratio is thought to act through the titration of a maternally-loaded repressor that controls the timing of ZGA in *Xenopus* [15] and *Drosophila* [16]. Changes in the concentration of the maternal transcription factor *tramtrack* (*ttk*) in *Drosophila* embryos, for example, are associated with changes in the timing of expression initiation of *fushi tarazu* (*ftz*), a gene that is required for proper segmentation of the embryo [17], because the action of *ttk* on the *ftz* promoter is repressive [16].

Another important factor during the MZT is the methylation repatterning process along the genome. Enzymes such as the DNA methyltransferases are essential for the maintenance of methylation patterns throughout cell divisions and creation of new methylation patterns through the methylation of previously unmethylated regions [18]. The same is true for the demethylation of previously methylated regions, which is partly increased by the semi-conservative DNA replication during cell divisions [19]. However, this latter process is less well understood. These dynamics are a potential source of changes in the methylation pattern throughout development, which may in turn affect gene expression and hence key developmental processes. Besides their key role during early embryo development, methylation dynamics continue to influence gene expression both in somatic cell lineages as well as in the germline during gametogenesis [20, 21]. The continuous dynamics in methylation patterns during gamete production in particular increase the chance of significant changes either due to mistakes or the incorporation of environmental effects experienced by the parents into the newly formed gametes. These changes create an association between parental environmental conditions and offspring phenotypes even at the very early developmental stages [8] and may therefore affect MZT.

In dioecious species, sex represents an additional level of complexity in methylation dynamics across generations for several reasons. First, because males and females produce different gametes, the zygote will experience a conflict between different sex-specific methylation patterns [22]. Second, due to the difference in gamete size, males and females have different quantitative and qualitative non-genetic contributions to the zygote (e.g. females contribute to most of the initial cytoplasmic material of the zygote) [23, 24]. Third, males and females may respond to environmental factors in different ways and might, therefore, transmit different epigenetic effects to the next generation. This parental conflict may have led to the evolution of genomic imprinting [25]. For example, while in mice the methylation state of some genes is inherited from the oocytes [26], in zebrafish the methylome of early embryos is inherited from the sperm [27, 28]. By modeling methylation dynamics across generations, we can hypothesize about the role of different methylation processes (e.g. repair methylation and de novo methylation) in embryonic development and investigate the consequences of sexual conflict in patterning the subsequence generation.

I developed a model of methylation dynamics throughout development including MZT through the addition of a nucleocytoplasmic ratio function and the effect of a parental factor (parental repressor) in a dioecious species to look at the dynamics of methylation processes during and across generations in order to understand how methylation and demethylation rates and transgenerational inheritance can affect development, in terms of global methylation levels.

## Materials and methods

The proposed model is based on the Otto and Walbot model of DNA methylation kinetics during the life cycle of an organism [29]. The model was modified in order to include sex, transgenerational and MZT components. In addition, I consider the effect of repair demethylation, active demethylation and sex-specific random environmental effects on methylation rates. These processes are explained in detail when types of methylation states are introduced in the methods. Each generation in the model represents the process of transformation in the cell lineage that goes from a zygote to a gamete in terms of global methylation levels. Changes in global methylation levels in this specific cell lineage is only used as an example. The model can also be interpreted in terms of locus-specific methylation levels. In fact, locus-specific methylation patterns have been shown to represent cell types accurately [30–32] because of the unique combinations of methylation states of different genes in different cell types. However, because methylation dynamics across loci are highly variable, global methylation levels seem to be a reasonable simplification for defining cell types. This is based on observations of global methylation levels as well as locus-specific methylation levels in different human normal tissues [30–33] and human tumors [34]. Nevertheless, the model is a general mechanism of change from one methylation level to another, so this assumption can be modified without affecting the functionality of the model. Additionally, the model can be interpreted in terms of different cell lineages or developmental stage intervals, in which cases a transgenerational effect is not possible. In other words, the model describes the changes in methylation levels from one stage to another, and here we define these stages as zygote and gamete, allowing us to include both intra- and transgenerational processes.

### Cell division dynamics

Two sets of equations are used subsequently to represent (i) the cell division dynamics and (ii) the methylation dynamics during cell divisions. We assume that transgenerational effects, when present, occur during the fertilization step at the end of each generation as they reflect environmental effects on rates of methylation coming from one or both parents and are transferred into the zygote through the gametes. During the maternal-to-zygotic transition, the initiation time of methylation dynamics during development is assumed to be delayed as a consequence of the dependence on the nucleocytoplasmic ratio *ν* [35, 36] and the parental repressor *ρ*, which is assumed to speed up cell divisions during the early stages of development and to delay the achievement of full methylation rates after the initiation of methylation changes as a consequence of the conflict between maternal and zygotic genomes. Although there is no empirical evidence for the cause of this delay, it is plausible to speculate that it might have evolved as a way to solve the male-female epigenomic conflict before the zygotic epigenome takes full control over development. However, this question needs to be properly addressed in future empirical studies.

Cell division dynamics follow a logistic growth process (Eq 1), as an approximation of division rates based on empirical data from the Northern leopard frog *Rana pipiens* and the loach *Misgurnus fossilis* [37, 38], with a variable growth rate (Eq 2) that depends on the amount of parental repressor in individual cells:

$$\begin{array}{c} N_{g,d+1}=N_{g,d}+\mu_{g,d}\cdot N_{g,d}\cdot \left(1-\frac{N_{g,d}}{K}\right) \end{array}$$ (1)

with

$$\begin{array}{c} \mu_{g,d}=\left(1+\rho_{g,d}\right)\cdot r \end{array}$$ (2)

where *N*_*g*,*d*_ is the number of cells in generation *g* and cell division *d*, *K* is the equilibrium developmental state in terms of number of cells, *μ* is the realized cell division rate, *ρ* is the amount of parental repressor and *r* is the intrinsic cell division rate.

The parental repressor *ρ* changes across cell divisions at a constant degradation rate *ρ*_*deg*_ (Eq 3), and its repressing effect *ρ*_*c*_ on methylation dynamics is a function of its current value *ρ*_*g*,*d*_ and initial value *ρ*_0_ (Eq 4), when *ρ*_0_ > 0. *ρ* is a dimensionless variable that represents the effect of the repressor on methylation rates and cell division rate, and decreases across cell divisions due to the degradation rate *ρ*_*deg*_. The effect of the repressor on cell division rate is one of the hypotheses that can explain the fast cell divisions during the early stages of development (as reviewed by [39]) and it has support from experimental studies (e.g. [40]). By assigning *ρ*_0_ = 1.0, we assume that the parental repressor causes an initial twofold increase in the speed of cell division, as indicated in Eq (2), and its effect decreases throughout development.

$$\begin{array}{c} \rho_{g,d+1}=\rho_{g,d}-\rho_{deg}\cdot \rho_{g,d} \end{array}$$ (3)

$$\begin{array}{c} \rho_{c_{g,d}}=\left\{ \begin{array}{cc} \frac{\rho_{0}-\rho_{g,d}}{\rho_{0}} & if\;\rho_{0}>0\\ 1 & if\;\rho_{0}=0 \end{array}\right. \end{array}$$ (4)

Given that cell divisions during the early stages of development happen without cell growth and hence the initial total cytoplasmic volume is conserved, the nucleocytoplasmic ratio *ν* can be calculated as a function of the number of cells (Eq 5).

$$\begin{array}{c} \nu_{g,d}=\frac{1}{N_{g,d}} \end{array}$$ (5)

According to experimental evidence [15], the maternal-to-zygotic transition (here assumed to be represented by methylation levels) is initiated when the nucleocytoplasmic ratio reaches a specific threshold (*ν*_*t*_). The process of ZGA assumes that the initial methylation level is conserved across cell divisions [28, 41, 42] and only changes when *ν* ≤ *ν*_*t*_. Once that is achieved, methylation dynamics towards lineage-specific methylation levels (e.g. gametic cell lineage) are initiated.

### Methylation dynamics

**Fig 1:**
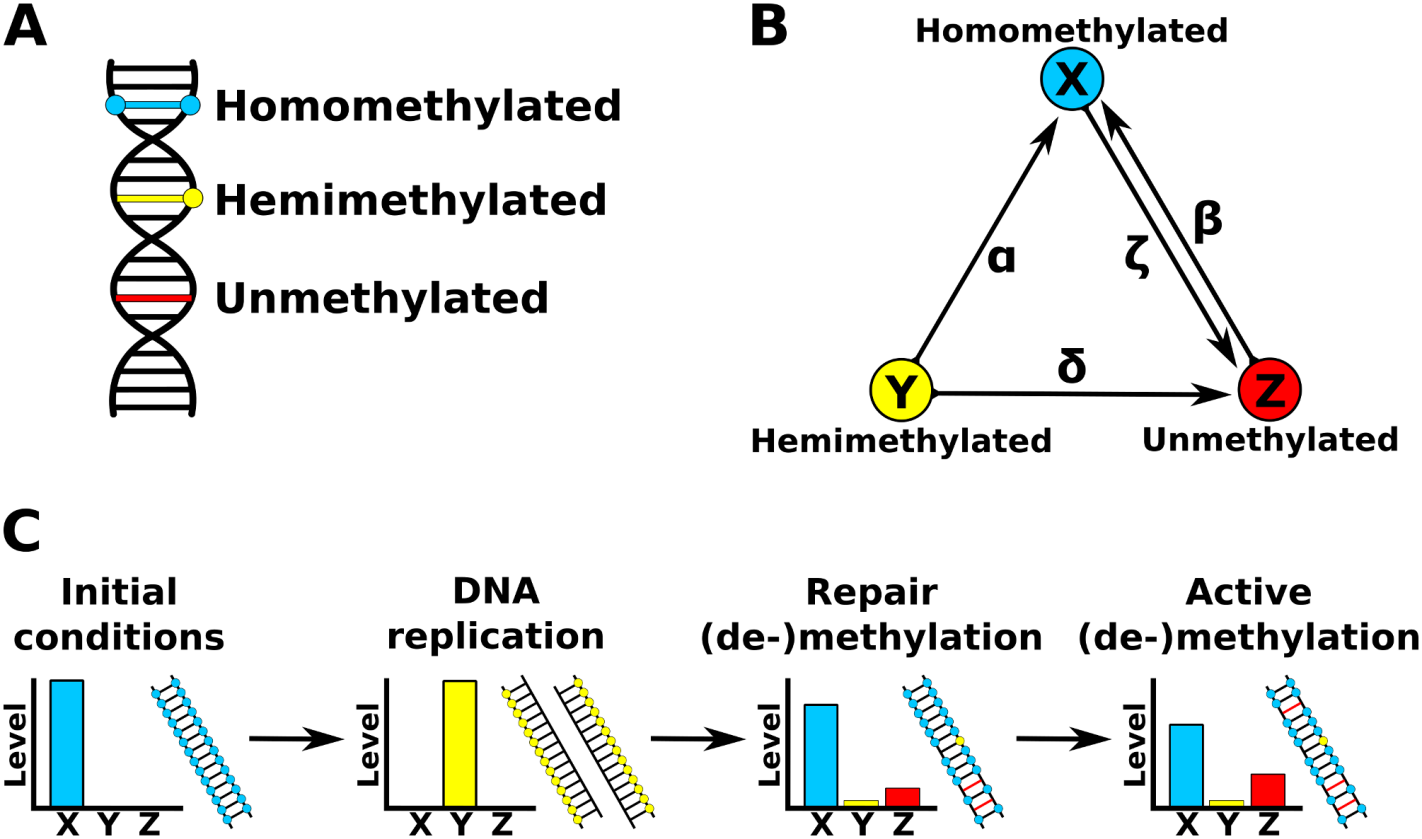
Model design. (A) Methylation dynamics variables present in the model. Three mutually exclusive methylation states are possible at each site. (B) Rates of conversion between the three mutually exclusive methylation states present in the model. (C) Sequence of events in the model and their representative effects on levels of different methylation states.

Three methylation states are considered in the model: (i) homomethylated (*X*), in which both strands of the DNA molecule are methylated; (ii) hemimethylated (*Y*), a transitional state in which only one of the strands is methylated; and (iii) unmethylated (*Z*), in which both strands are unmethylated (Fig 1A). Rates of conversion between methylation states are represented in Fig 1B. The rates *α* (repair or maintenance methylation) and *δ* (repair or maintenance demethylation) represent the conversion of hemimethylated sites into homomethylated and unmethylated sites, respectively. The rates *β* (active methylation) and *ζ* (active demethylation) represent the conversion of unmethylated sites into homomethylated sites and of homomethylated sites into unmethylated sites, respectively. Due to the lack of empirical information about the active generation of hemimethylated sites and the complications that potential active hemimethylation processes would bring into the model, the model assumes that hemimethylated sites are a consequence of DNA replication and are treated as transitional, that is, hemimethylated sites arise from homomethylated sites during DNA replication. Although a recent study has shown that a small number of hemimethylated sites is maintained after DNA replication and inherited across cell divisions [43], it is unclear whether active hemimethylation is a functional process or a failure in the de novo/maintenance methylation of both DNA strands.

These methylation and demethylation rates have different values before and after ZGA. Regardless of zygotic methylation levels, Pre-ZGA *α* in sex *i* (*α*_*i*_0__) is set to *α*_*i*_0__ = 1.0, while pre-ZGA *β* (*β*_*i*_0__), *δ* (*δ*_*i*_0__) and *ζ* (*ζ*_*i*_0__) are set to zero in order to simulate the approximately constant methylation level (*X* + *Y*) across cell divisions before the onset of the ZGA that has been observed in empirical studies [28, 41]. By setting *α*_*i*_0__ = 1.0 (maximum rate of maintenance methylation) while keeping the other (de-)methylation processes inactive, the model assumes that the methylation pattern observed during ZGA is possible only when enzymatic activity analogous to *α* compensate for the demethylation caused by DNA replication during cell divisions and enzymatic activity analogous to *δ* and *ζ*, maintaining methylation levels near zygotic levels until ZGA. A similar effect can be achieved with a more complicated scenario in which the rate of de novo methylation is equal to the rates of active demethylation and passive demethylation (caused by DNA replication), resulting in a constant methylation level across cell divisions.

Post-ZGA rates (*α*_*i*_1__, *β*_*i*_1__, *δ*_*i*_1__ and *ζ*_*i*_1__) are characteristic of specific final methylation levels (cell types). Changes in methylation and demethylation rates throughout development are represented by the logistic Eqs (6)–(9), that model the transition between pre-ZGA and post-ZGA rates:

$$\begin{array}{c} \alpha_{i_{g,d}}=\frac{\alpha_{i_{1}}-\alpha_{i_{0}}}{1+\exp(-Q_{\alpha }\cdot \left(\rho_{power}\cdot \frac{\rho_{0}}{\rho_{g,d}}-\frac{1}{\nu_{t}}\right))}+\alpha_{i_{0}} \end{array}$$ (6)

$$\begin{array}{c} \beta_{i_{g,d}}=\frac{\beta_{i_{1}}-\beta_{i_{0}}}{1+\exp(-Q_{\beta }\cdot \left(\rho_{power}\cdot \frac{\rho_{0}}{\rho_{g,d}}-\frac{1}{\nu_{t}}\right))}+\beta_{i_{0}} \end{array}$$ (7)

$$\begin{array}{c} \delta_{i_{g,d}}=\frac{\delta_{i_{1}}-\delta_{i_{0}}}{1+\exp(-Q_{\delta }\cdot \left(\rho_{power}\cdot \frac{\rho_{0}}{\rho_{g,d}}-\frac{1}{\nu_{t}}\right))}+\delta_{i_{0}} \end{array}$$ (8)

$$\begin{array}{c} \zeta_{i_{g,d}}=\frac{\zeta_{i_{1}}-\zeta_{i_{0}}}{1+\exp(-Q_{\zeta }\cdot \left(\rho_{power}\cdot \frac{\rho_{0}}{\rho_{g,d}}-\frac{1}{\nu_{t}}\right))}+\zeta_{i_{0}} \end{array}$$ (9)

where *i* indicates sex (male or female), *Q* is the slope of the rate transition from pre-ZGA values to post-ZGA values, with pre-ZGA rates indicated by the subscript 0 and post-ZGA rates indicated by the subscript 1, and *ρ*_*power*_ is the magnitude of the effect of *ρ* on triggering the transition, with high *ρ*_*power*_ indicating a rapid transition (and it depends on the locus of interest in the locus-specific version).

Three sequential genomic events happen during a defined number of cell cycles (Fig 1C). The dynamics of these events are sex specific and change once MZT is initiated with ZGA. The first step is DNA replication (Eqs 10–12), in which the double-stranded molecule is replicated in a semiconservative way. During this step, all homomethylated sites generate hemimethylated sites, hemimethylated sites generate equal proportions of hemimethylated and unmethylated sites, and all unmethylated sites generate unmethylated sites.

$$\begin{array}{c} X_{id_{g,d+1}}=\frac{X_{i_{g,d}}\cdot 0}{2} \end{array}$$ (10)

$$\begin{array}{c} Y_{id_{g,d+1}}=\frac{2X_{i_{g,d}}+Y_{i_{g,d}}}{2} \end{array}$$ (11)

$$\begin{array}{c} Z_{id_{g,d+1}}=\frac{2Z_{i_{g,d}}+Y_{i_{g,d}}}{2} \end{array}$$ (12)

The second step is repair or maintenance methylation and demethylation (Eqs 13–15), in which hemimethylated sites are converted into homomethylated sites or unmethylated sites at rates *α* and *δ*, respectively. The model assumes that the proportion of unmethylated sites is positively correlated with expression levels, so *δ* is affected by the level of parental repressor *ρ*_*c*_. This assumption provides a functional interpretation for the model and is based on empirical studies [4, 44]. However, when the model is viewed purely in terms of methylation dynamics, this assumption can be ignored without compromising the functionality of the model.

$$\begin{array}{c} X_{ir_{g,d+1}}=\alpha_{i_{g,d}}\cdot Y_{id_{g,d+1}}+\beta_{i_{g,d}}\cdot Z_{id_{g,d+1}} \end{array}$$ (13)

$$\begin{array}{c} Y_{ir_{g,d+1}}=\left(1-\alpha_{i_{g,d}}-\rho_{c_{g,d}}\cdot \delta_{i_{g,d}}\right)\cdot Y_{id_{g,d+1}} \end{array}$$ (14)

$$\begin{array}{c} Z_{ir_{g,d+1}}=\left(1-\beta_{i_{g,d}}\right)\cdot Z_{id_{g,d+1}}+\rho_{c_{g,d}}\cdot \delta_{i_{g,d}}\cdot Y_{id_{g,d+1}} \end{array}$$ (15)

Since homomethylated sites do not exist at this step as a consequence of DNA replication, only the conversion from unmethylated to homomethylated states (active methylation) happen at a rate *β*, while conversion from the homomethylated to the unmethylated state happens at a rate *ζ* during the next step, called active demethylation (Eqs 16–18). The process goes on for a constant number of cell divisions *D* each generation.

$$\begin{array}{c} X_{im_{g,d+1}}=\left(1-\zeta_{i_{g,d}}\right)\cdot X_{ir_{g,d+1}} \end{array}$$ (16)

$$\begin{array}{c} Y_{im_{g,d+1}}=Y_{ir_{g,d+1}} \end{array}$$ (17)

$$\begin{array}{c} Z_{im_{g,d+1}}=\left(Z_{ir_{g,d+1}}+\zeta_{i_{g,d}}\cdot X_{ir_{g,d+1}}\right) \end{array}$$ (18)

### Fertilization

At the end of each generation *g*, there is a fertilization step (Eq 19), triggered by a Boolean threshold function *ϕ*. The threshold function allows fertilization to happen when the number of cell divisions *d* reaches the specified constant number of divisions *D* per generation. The model can be modified so that fertilization happens as soon as the equilibrium methylation levels are reached, but the former option was chosen as a simplification.

$$\begin{array}{c} \phi =\left\{ \begin{array}{cc} 0 & if\;d\neq D\\ 1 & if\;d=D \end{array}\right. \end{array}$$ (19)

During the fertilization step, the initial methylation level of generation *g* + 1 is set to the average of the final methylation levels of male and female of generation *g* (Eqs 20–22).

$$\begin{array}{c} X_{i_{g+1,1}}=\phi \cdot \frac{X_{mm_{g,D}}+X_{fm_{g,D}}}{2} \end{array}$$ (20)

$$\begin{array}{c} Y_{i_{g+1,1}}=\phi \cdot \frac{Y_{mm_{g,D}}+Y_{fm_{g,D}}}{2} \end{array}$$ (21)

$$\begin{array}{c} Z_{i_{g+1,1}}=\phi \cdot \frac{Z_{mm_{g,D}}+Z_{fm_{g,D}}}{2} \end{array}$$ (22)

Based on this model, the number of cells and the proportion of homomethylated sites are expected to reach an equilibrium during development. Due to the sex specific parameters of the model, the methylation level equilibrium will also be sex specific, which will cause the methylation state proportions to diverge towards their respective sex specific equilibria as soon as the nucleocytoplasmic ratio (*ν*) threshold and critical levels of *ρ* are reached, triggering the ZGA.

Although the simulations use different methylation levels to characterize males and females, there is no intrinsic qualitative distinction between sexes in the model. Depending on the parameter values used, males and females might eventually reach equal methylation levels. However, the methylation level alone does not account for the qualitative pattern of methylation and may represent completely different sets of genes for different sexes. We ignore qualitative differences between sexes and assume that sex is a genetic property of the system, not a variable epigenetic property. In other words, sex is not determined by the methylation levels and, therefore, levels of males and females can potentially reach any value within the range of methylation levels without implying sex conversion.

The model assumes that the rates of methylation and demethylation are constant throughout development but not necessarily across generations. These rates represent the average rate of modification from the zygotic to the gametic methylation level. At the end of every generation, the rates of methylation and demethylation can be set to change randomly within a specified range. The environment can have a directional effect rather than random, forcing the levels of methylation to go up or down or remain stable as long as environmental conditions are stable. Variable values used in the simulations are shown in Table 1. The model and the simulations were developed on R version 3.3.3 [45]. The R script is available in S1 File.

**Table 1:** Description of parameters and values used in the model simulations to obtain Figs 2–6.

Variable	Description	Value
*D*	Number of cell divisions per generation.	250
*r*	Intrinsic cell division rate.	0.1
*K*	Equilibrium number of cells during development.	1024
*ρ*_0_	Initial amount of parental repressor.	1.0
*ρ*_*deg*_	Intrinsic rate of degradation of parental repressor.	0.1
*ρ*_*power*_	Magnitude of the effect of parental repressor in triggering the ZGA.	1.0
*Q*_*α*,*β*,*δ*,*ζ*_	Slope of rate transition from pre-ZGA to post-ZGA values.	100
*ν*_*t*_	Nucleocytoplasmic ratio threshold.	0.01
*X*_*i*0_	Initial proportion of homomethylated sites {male, female}.	{1.0, 1.0}
*Y*_*i*0_	Initial proportion of hemimethylated sites {male, female}.	{0.0, 0.0}
*Z*_*i*0_	Initial proportion of unmethylated sites {male, female}.	{0.0, 0.0}
*α*_*i*_0__	Pre-ZGA rate of conversion of hemimethylated into homomethylated sites after DNA replication {male, female}.	{1.0, 1.0}
*δ*_*i*_0__	Pre-ZGA rate of conversion of hemimethylated into unmethylated sites after DNA replication {male, female}.	{0.0, 0.0}
*β*_*i*_0__	Pre-ZGA rate of conversion of unmethylated into methylated sites after DNA replication {male, female}.	{0.0, 0.0}
*ζ*_*i*_0__	Pre-ZGA rate of de novo conversion of methylated into unmethylated sites after DNA replication {male, female}.	{0.0, 0.0}
*α*_*i*_1__	Post-ZGA rate of conversion of hemimethylated into homomethylated sites after DNA replication {male, female}.	{0.99, 0.99}
*δ*_*i*_1__	Post-ZGA rate of conversion of hemimethylated into unmethylated sites after DNA replication {male, female}.	{0.01, 0.01}
*β*_*i*_1__	Post-ZGA rate of conversion of unmethylated into methylated sites after DNA replication {male, female}.	{0.04, 0.1}
*ζ*_*i*_1__	Post-ZGA rate of de novo conversion of methylated into unmethylated sites after DNA replication {male, female}.	{0.0, 0.0}
*α*_*envmin*_, *α*_*envmax*_	Range of additive random variation in *α* across generations.	[-0.01, 0.01]
*δ*_*envmin*_, *δ*_*envmax*_	Range of additive random variation in *δ* across generations.	[-0.01, 0.01]

### Empirical analogy

Additionally, I propose an equation (Eq 23) that draws an analogy between empirical measurements and the (de-)methylation rates used in the proposed model. This equation calculates the speed of the enzymatic activity (*A*) responsible for (de-)methylation dynamics, and can be used to measure the average (de-)methylation rates between two developmental stages:

$$\begin{array}{c} A=\frac{\left(X_{S1}-X_{S0}\right)\cdot L_{n}\cdot H}{T_{d}} \end{array}$$ (23)

where *A* is the average speed of conversion between homomethylated and unmethylated states, *X*_*S*0_ and *X*_*S*1_ are the global proportions of homomethylated sites at developmental stages *S*0 and *S*1, respectively, *L*_*n*_ is the total haploid genome-wide CpG length (number of CpG sites), *H* is the ploidy of the organism (*H* = 2 for diploid organisms), and *T*_*d*_ is the interval between developmental stages *S*0 and *S*1.

The variables in Eq (23) can be used with different units, depending on the question of interest. For example, *T*_*d*_ can be measured as units of time or number of cell divisions, and *X*_*S*0_ and *X*_*S*1_ can be measured at two developmental stages or two cell divisions (for higher accuracy). When *A* > 0, the overall methylation activity is higher than the demethylation activity; the opposite is true when *A* < 0. With Eq (23), it is possible to infer the relative activity levels of enzymes responsible for (de-)methylation processes throughout development.

## Results

**Fig 2:**
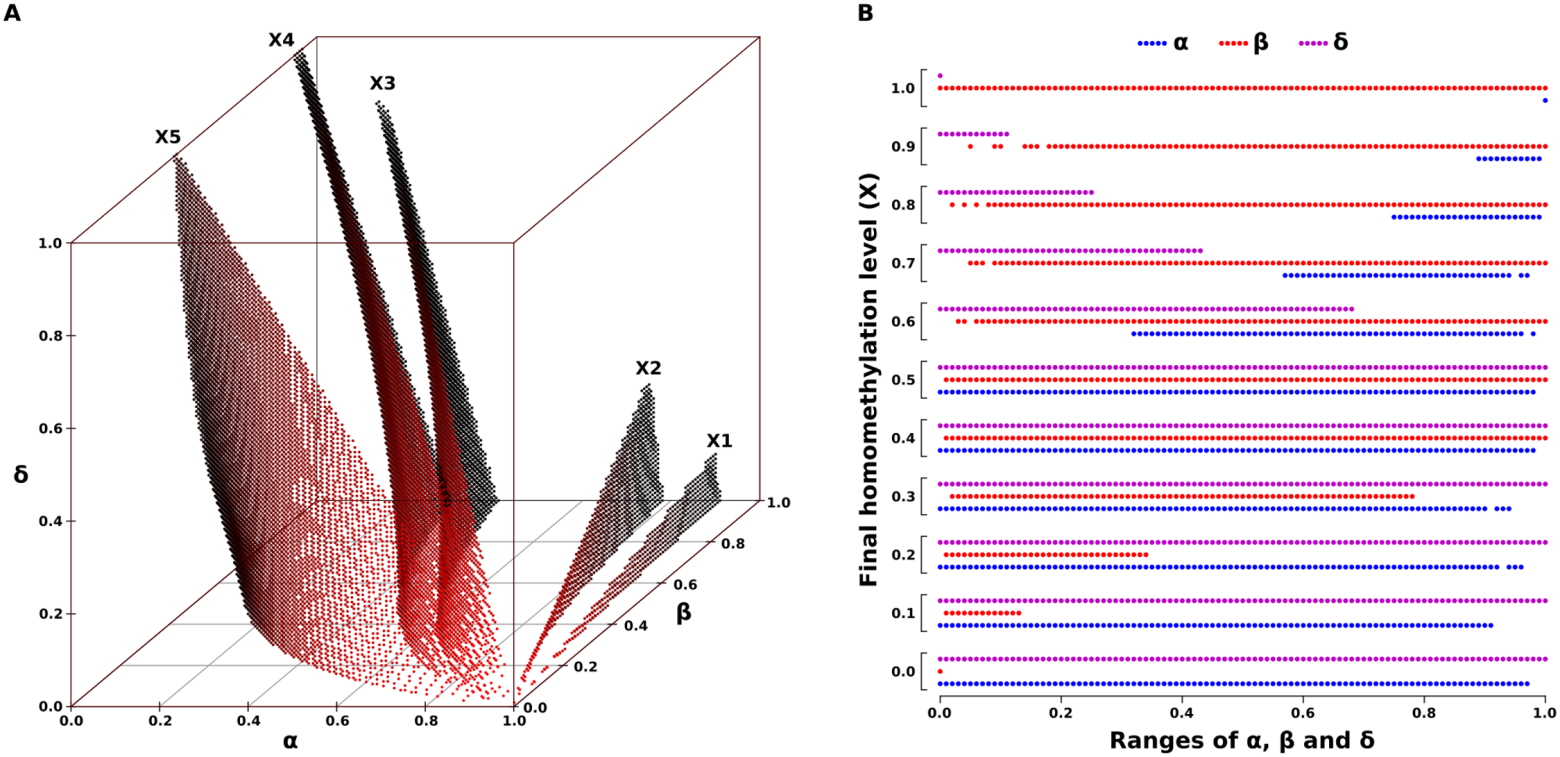
Possible combinations of values of *α*, *β* and *δ* that result in different gametic homomethylation levels (A) and ranges of *α*, *β* and *δ* values which can yield different homomethylation levels (B). (X1) *X* = 0.91 (e.g. zebrafish sperm), (X2) *X* = 0.80 (e.g. zebrafish oocytes), (X3) *X* = 0.54 (e.g. human sperm), (X4) *X* = 0.48 (e.g. human oocyte), and (X5) *X* = 0.30 (hypothetical gamete with low methylation level).

We performed a parameter exploration of the model to identify parameter values that yield the specific final methylation levels of different gametes during the first generation of the simulation. The exploration was carried out assuming methylation levels as described for those in gametes of zebrafish and human. However, as mentioned in the model description, global methylation levels of gametes are only used because it avoids the highly variable (de-)methylation rates across loci and can include transgenerational effects. In zebrafish, the oocytes are hypomethylated (methylation level = 0.80) compared to sperm (methylation level = 0.91) [28]. In humans, methylation levels are 0.54 in sperm and 0.48 in metaphase II oocytes [46]. The methylation level of chimpanzee sperm is 0.67 [47] but this information was not included in the analyses due to the absence of information for chimpanzee oocytes. Information from experimental studies on methylation levels in different species was assumed to represent the proportion of homomethylated sites in the genome. The combinations of *α*, *β* and *δ* values that result in the given methylation levels in zebrafish and human gametes were estimated to an accuracy of 0.01 (Fig 2), as well as a hypothetical gamete (*X* = 0.30) to account for species with low levels of methylation, e.g. *Drosophila melanogaster*, which has been reported to show near-zero levels [48]. Because the effect of *ζ* is analogous to DNA replication (contributes to demethylation) and takes place in the last step before every cell division and fertilization, and therefore affects the final methylation level of every cycle, its value was not estimated and a value of zero was taken as default, unless stated otherwise. However, *ζ* is an essential part of methylation dynamics, and therefore must be taken into consideration in the explanation of the process. Methylation levels of zebrafish gametes were taken as an example to run the simulations and one of the possible combinations of *α*, *β* and *δ* values was chosen randomly as a model default.

Variation in the level of homomethylation as observed in zebrafish and human gametes (accuracy of 0.01) requires rather distinct parameter combinations (*α*, *β* and *δ*), leading to decreasing three-dimensional parameter spaces with increasing homomethylation levels (Fig 2A). The ranges of the parameter values also depend on the expected levels of homomethylation, which might reflect the flexibility of the system in the achievement of the optimal levels. We generally found that the rates of methylation *α* and demethylation *δ* repair required to achieve high homomethylation levels were more restricted than the rate of *de novo* methylation *β* (Fig 2B), whereas for intermediate levels of homomethylation, values of *α*, *β* and *δ* yielding the predicted levels vary across the entire range of the individual parameter dimensions. These results indicate that there is a developmental constraint on the transition between two levels of methylation, which can potentially represent different cell types.

**Fig 3:**
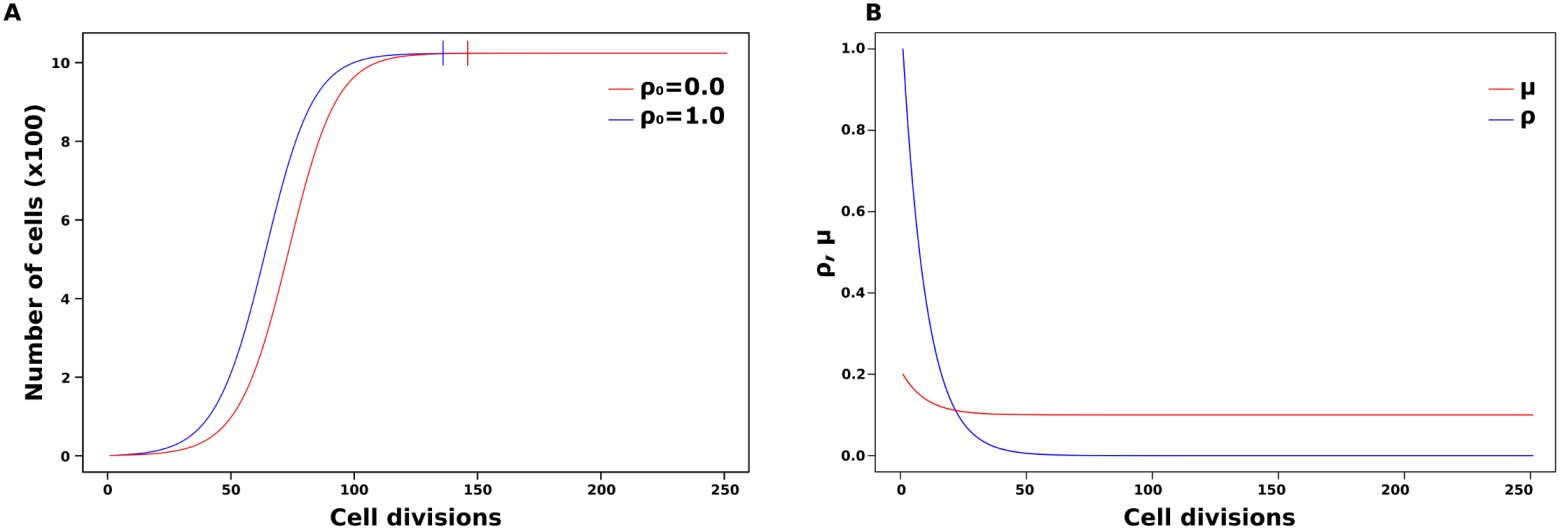
Effect of the parental repressor on cell division speed. (A) Comparison of a cell division dynamics model with the effect of parental repressor (*ρ*_0_ = 1.0) and without (*ρ*_0_ = 0). (B) Change in amount of parental repressor *ρ* and speed of cell division *μ* throughout development, with *ρ*_*deg*_ = 0.1. Vertical bars mark cell divisions at which the equilibrium is achieved.

When it comes to the cell division dynamics and the effect of parental repressor, the simulations show how the amount of parental repressor can speed up development and possibly advance ZGA compared to the standard logistic growth model. In the simulations, the parental repressor is set to increase up to double (*ρ*_0_ = 1.0) the intrinsic division rate *r*. More precisely, the simulations showed an advancement of 10 cell divisions to the equilibrium number of cells (from 146 to 136 cell divisions) when the parental repressor is set to double cell division rate. Because the parental repressor is continuously degraded and titrated during every cell division, its effect is limited to the very first cell divisions. As a consequence, the rate of cell division sharply decreases during the first divisions due to the exponential decrease in the amount of repressor per cell (Fig 3). However, an increase in cell division speed due to higher initial levels of parental repressor can advance the onset of ZGA because it will affect the timing of change in the nucleocytoplasmic ratio.

**Fig 4:**
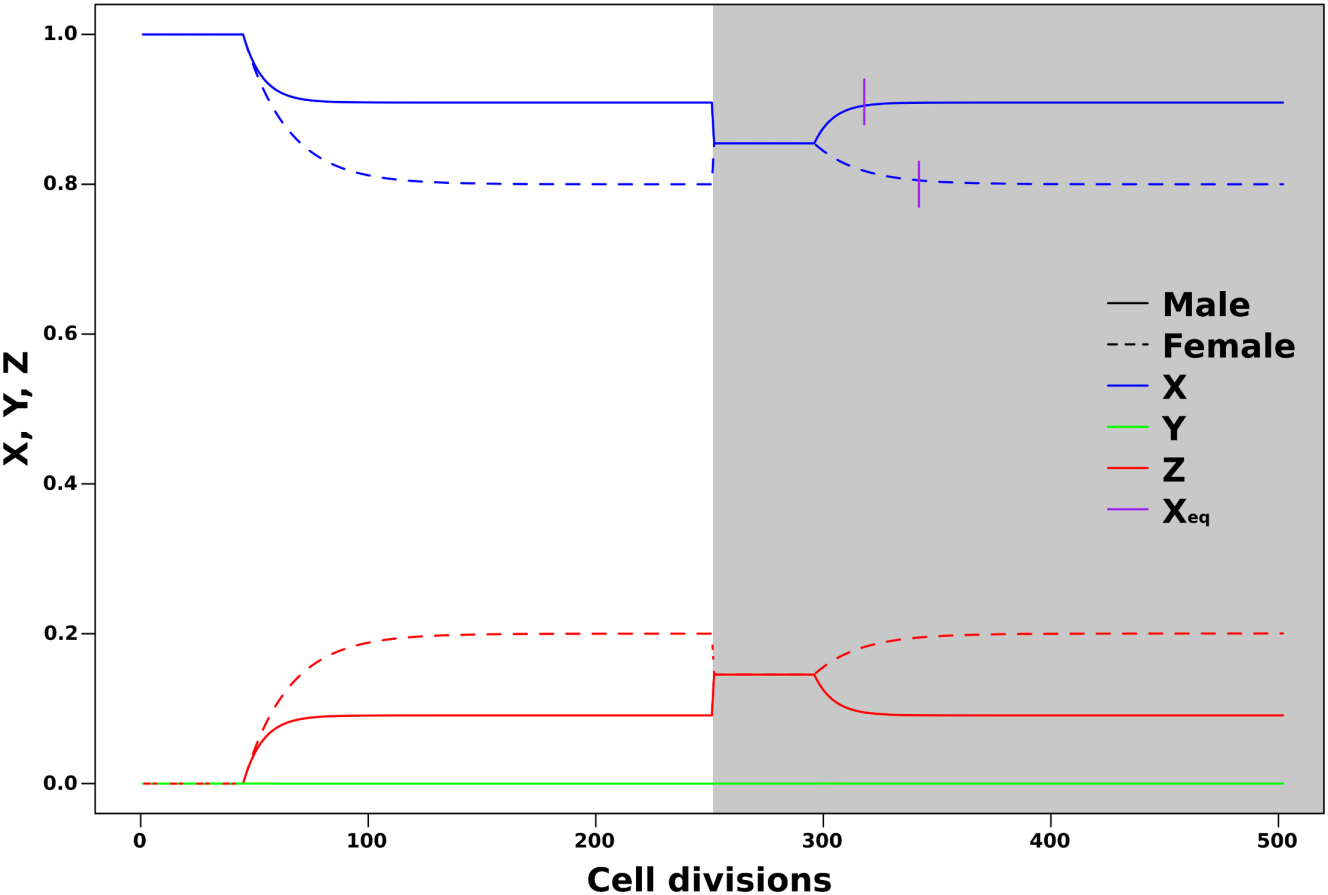
Methylation dynamics between sexes (dashed lines vs. solid lines) during development and across two generations (shades of grey) with 250 cell divisions each. Parameter values used in the simulations are specified in Table 1. Vertical bars mark cell divisions at which the equilibrium is achieved.

Once ZGA is triggered by the critical nucleocytoplasmic ratio and levels of the parental repressor, the post-ZGA methylation processes lead to directional changes in the three methylation states at the defined rates. The methylation dynamics patterns observed in the simulations are very similar to the patterns observed in experiments in zebrafish [28]. Throughout development, the methylation levels in males and females approach an equilibrium value, which is equivalent to the methylation levels of specific cell types (e.g. gametes). The following generation starts with a value reflecting the average between male and female gametes (Fig 4). During the second generation, methylation levels reach the equilibrium states of *X* = 0.91 (dashed line) and *X* = 0.80 (solid line) after 67 and 91 cell divisions, respectively.

**Fig 5:**
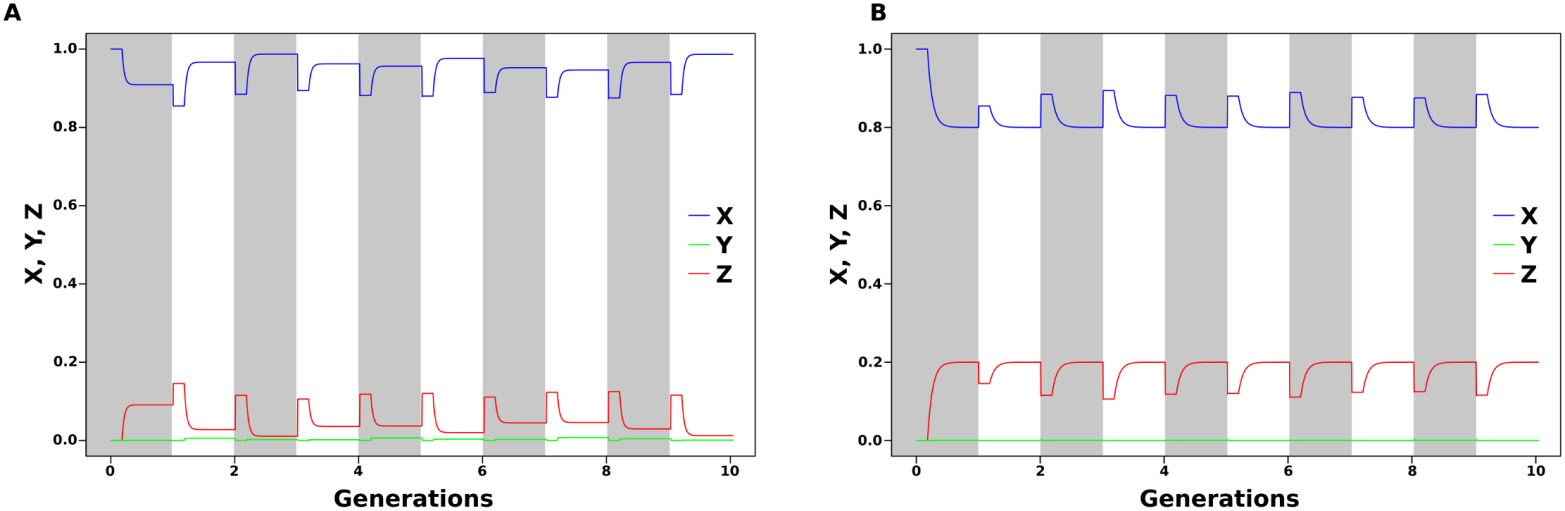
The effect of random changes in *α* and *δ* across generations in one sex (A, female) does not affect the final methylation level of the adults in the other sex (B, male). Plot shows 10 generations (shades of grey) of 250 cell divisions each.

By allowing changes in methylation rates in one sex, we can simulate environmental effects on methylation levels across generations. In Fig 5, random changes in rates of methylation and demethylation repair in females cause changes in the final methylation state of females in the parental generation, which affect the initial methylation state of the next generation. However, given that males keep their methylation rates constant, their final methylation level continues constant across generations, regardless of the changes in methylation rates of females. This effect can be assumed to happen when methylation rates are controlled by sex-linked loci. When environmental conditions can affect only one sex, that particular sex will show the effect in the next generation, while the final methylation level of the other sex will be unaffected relative to the level in the previous generation. When both sexes are sensitive to the effect of environmental conditions on methylation rates, the pattern of random variation observed in Fig 5A is observed in both sexes, which show variable gametic methylation levels across generations.

**Fig 6:**
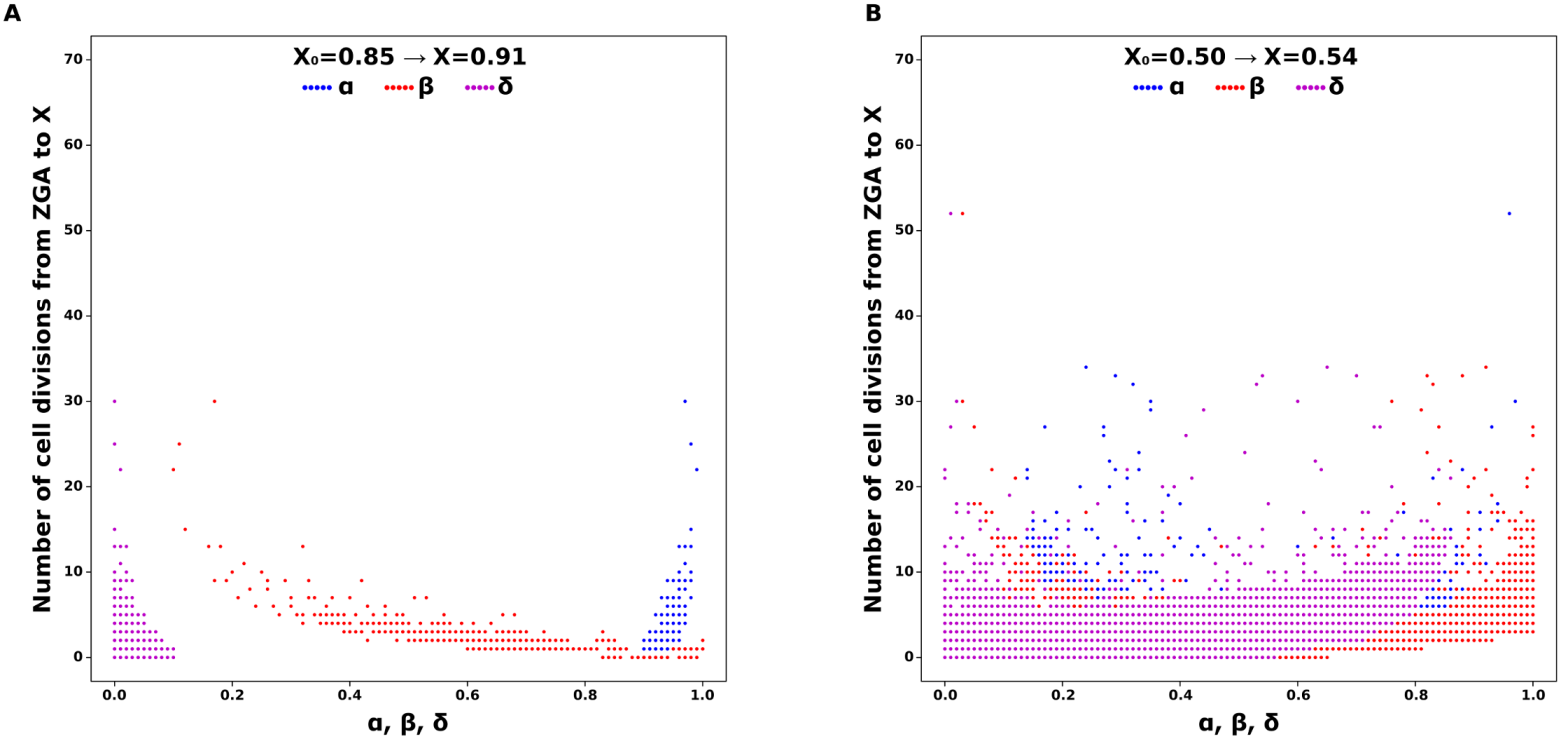
Number of cell divisions necessary to reach a specific value of homomethylation level (X) depending on values of *α*, *β* and *δ*. Parameter values used in the simulations are specified in Table 1.

The methylation states will reach the equilibrium values as long as enough cell divisions are allowed to occur before fertilization and the methylation dynamics rates are held constant throughout development. The value of the nucleocytoplasmic ratio will also affect the number of divisions after fertilization required to reach a stable methylation level, and the value of *ρ*-related parameters can advance or delay the onset of ZGA. However, once post-ZGA methylation dynamics are initiated, only very few cell divisions (22 and 46 post-ZGA cell divisions for *X* = 0.91 and *X* = 0.80, respectively) are sufficient to reach a stable methylation value (at a given accuracy). The number of cell divisions to reach a specific homomethylation level varies for different combinations of *α*, *β* and *δ* and for different values of X (Fig 6). This can be explained by possible species-specific limitations in (de-)methylation activity, with high activity levels needing a smaller number of cell divisions to achieve certain methylation levels.

The (de-)methylation activity of zebrafish during particular developmental periods can be roughly estimated using Eq (23). The zebrafish haploid genome contains approximately 24.2 million CpG sites [28]. There is little change in global methylation levels between zygote and 32-cell stages, with *A* = ∼0.0, which means that enzymatic activity responsible for (de-)methylation remained constant during this developmental interval. However, between 32-cell (*X* = ∼0.85) and 128-cell (*X* = ∼0.87) stages (an interval of two cell divisions), the net methylation activity is different from zero, with an average increase in methylation activity of *A* = ∼48.4 ⋅ 10^4^ CpGs/cell division.

## Discussion

The proposed model of methylation dynamics from fertilization to the adult germline is the first to approach MZT from a theoretical perspective and the first to link the processes occurring during early development with transgenerational dynamics. It provides a set of predictions about the possible mechanisms involved in the transition from a mixed methylome in the zygote into a specialized methylome in the adult gametes, including different types of methylation and demethylation processes. We show that variation in average rates of methylation affects the optimal methylation levels characteristic of specific cell types, which may explain the variation observed across different species. I used the model to compare the methylation levels of zebrafish and human gametes and identified the specific combinations of methylation rates resulting in the respective methylation levels.

**Fig 7:**
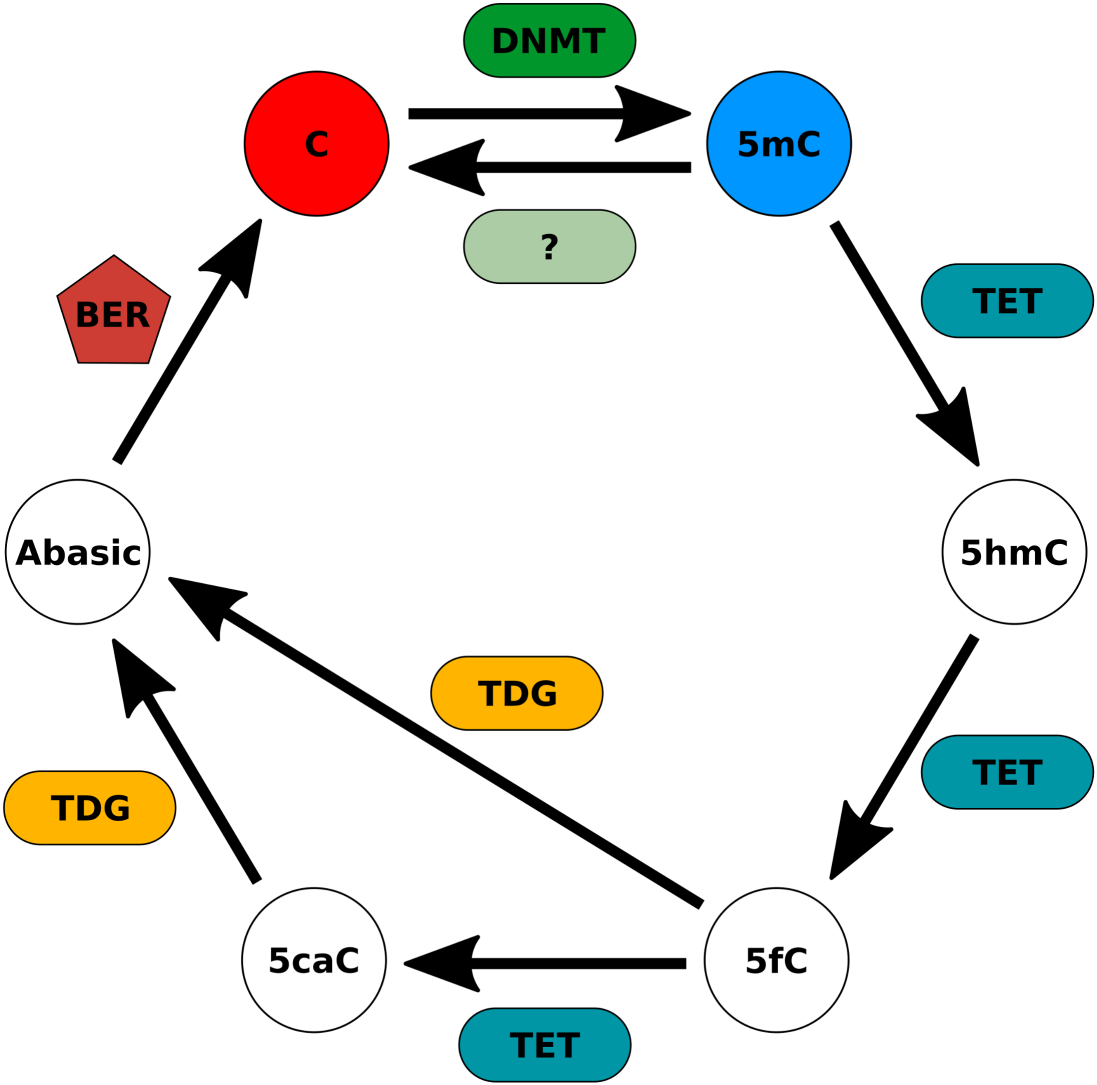
Enzymes that can possibly play the role of the rates *α*, *β*, *δ* and *ζ* used in the model. The question mark indicates the possible existence of an enzyme that directly converts 5-methylcytosine into cytosine. C—Cytosine; 5mC—5-Methylcytosine; 5hmC—5-Hydroxymethylcytosine; 5fC—5-Formylcytosine; 5caC—5-Carboxylcytosine; Abasic—Abasic site; DNMT—DNA methyltransferase; TET—Ten-eleven translocation enzymes; TDG—Thymine DNA glycosylase; BER—Base excision repair.

I show that higher levels of methylation in a cell type require higher rates of maintenance methylation (*α*) and *de novo* methylation (*β*) during development, and the number of cell divisions necessary to reach specific levels strongly depend on the initial conditions of the model. These findings are supported by the fact that enzymatic activity involved in active (de-)methylation processes are necessary throughout development [21, 26, 49, 50] and that these rates are the direct result of the activity of enzymes like the DNA methyltransferases [51, 52]. Enzymes that have been shown to be involved in these processes are represented in Fig 7 [42, 53].

The methylation dynamics process in the model is part of the transition that converts the average parental methylation level present in the zygote right after fertilization into the specialized methylation levels of gametes in the adult offspring. The model takes different rates of methylation transformation into consideration, including methylation maintenance after DNA replication. Given that a methylation maintenance rate of *α* = 1.0 (no errors) would maintain the methylation state of subsequent cell generations equal to the zygotic state (or above if the active methylation rate *β* > 0, until it reaches a fully methylated genome state), it is possible that the evolution of methylation-dependent developmental processes such as gene expression [1, 54, 55] has benefited from an imperfect methylation maintenance mechanism (*α* < 1.0) because of the possible contribution to gene expression pattern diversity and potentially new gene interactions. However, methylation maintenance errors are random and can cause embryo inviability. A more plausible explanation is therefore that a near-perfect methylation maintenance mechanism (*α* ≈ 1.0) would require a highly active and precise mechanism of demethylation in order to generate the diversity of methylation patterns, both quantitatively and qualitatively, that is typically observed in different cell types present in a single organism [31, 33, 34]. Experimental studies have shown that active methylation and demethylation processes are necessary for normal development in mice and zebrafish [26, 56, 57] and that there is an increasing preference for homomethylated sites over hemimethylated sites throughout development as cells differentiate, indicating an increase in epigenetic stability [58]. This supports the idea that high maintenance methylation rates are essential for achieving differentiated cell types. In addition, methylation levels in sperm have been shown to affect pregnancy rates in *in vitro* fertilization results in humans [59], indicating the necessity for an accurate methylation process. The model provides information about the accuracy of the system in terms of the flexibility of methylation rates to achieve specific methylation levels, showing that low final methylation levels can be achieved by a broader range of maintenance (de-)methylation rates, while high final methylation levels require a very narrow range of the same rates.

Besides within generation methylation dynamics, I also simulated the maternal-to-zygotic transition across generations where random environmental effects on one of the parents may affect methylation rates in the subsequent generation. The model predicts that, although both sexes would be affected, the sex with constant rates across generations would only be affected in the initial conditions of every generation but would eventually recover its original adult methylation pattern. That happens because the change in the gametic pattern of the sex with variable rates would only interfere in the initial state of the sex with constant rate. This is a consequence of the model assumption that methylation rates are sex-specific and transmitted in a *cis* fashion, that is, changes in methylation rates of one sex will only affect the rates of offspring of the same sex in the next generation. This assumption is based on the fact that male and female gametes show characteristic methylation levels in zebrafish [28] and humans [46] and must therefore have sex specific methylation rates. The model also assumes that the methylation process is independent from the methylation pattern of the zygotic genome, which might not be the case *in vivo*.

The model also included three factors involved in the initiation of zygotic transcription: (i) the nucleocytoplasmic ratio in the growing embryo, (ii) the effect of a parental repressor on the speed of cell division (positive effect) and methylation dynamics (negative effect), and (iii) the intrinsic rate of degradation of parental products (such as mRNAs and enzymes etc.). Although experiments have demonstrated that the nucleocytoplasmic ratio is indeed linked to the transcriptional activation of the zygotic genome both in vertebrates [15] and invertebrates [16], the nucleocytoplasmic ratio *per se* is not a direct physical mechanism of activation, but a measurement that is associated with the actual triggering mechanism. It is currently not known how the nucleocytoplasmic ratio is linked to the ZGA and in the proposed model it is used as a trigger for the transition from pre-ZGA methylation rates to post-ZGA rates. Nevertheless, the exact role of the nucleocytoplasmic ratio needs further investigation to verify the assumptions made in the model. Additionally, as observed in previous studies, the parental repressor can delay methylation dynamics [60] and increase the cell division rate (reviewed by [61]), providing an empirical basis for its function in the model. The presence of parental products in the cytoplasm (in particular, maternal products) has been shown to be important for MZT, which has been supported by transcriptome studies on early developmental stages. In *Xenopus*, the presence of functional cytoplasmic products of maternal origin has been reported [12, 15] but little is known about the specific roles of these mRNAs and proteins in the control of ZGA. In the proposed model, the intrinsic rate of degradation of parental product can be interpreted as the action of any parental product responsible for the acceleration of the degradation process.

These three factors discussed above are among the several hypotheses that have been proposed to explain how ZGA is triggered, but experimental studies have only elucidated a small part of the process, providing clues about the possible mechanisms that might be in effect during the transition [62, 63]. Future experimental studies are necessary to fully elucidate all the factors and processes involved, including the actual rates and limits of enzymatic activity involved in (de-)methylation throughout development. The proposed model provides a theoretical hypothesis for the role of the parental repressor in MZT as well as a prediction of the flexibility of the methylation dynamics in different species based on the methylation level of their gametes.

When it comes to gene expression during the MZT, methylation levels may be an indication of transcription levels of genes regulated by such epigenetic mechanism. However, as stated previously, gene expression is a complex process that is not only controlled by methylation patterns but also by several other regulatory mechanisms, e.g. histone modifications [5], which need to be taken into consideration when comparing the model results with future empirical observations. Furthermore, methylation patterns seem to work in different ways depending on their locations (e.g. promoter, gene body) [64, 65], demanding more in-depth empirical investigations on the role of methylation across the genome.

Although the mechanism through which methylation regulates gene expression is still unclear, its importance has gained experimental support from studies in which methylation processes are disturbed [66, 67]. Nevertheless, it is important to note that the diversity of processes regulating gene expression demands more complex experimental and theoretical approaches. Furthermore, questions about specific loci need locus-specific empirical data in order to draw conclusions about the locus expression dynamics throughout development. The same is true for global expression dynamics. That said, the proposed model can fail to predict expression levels when, for example, histone modifications and RNA interference outperform the effect of methylation, which can also happen throughout MZT, during pre-ZGA and post-ZGA.

The model focuses on global methylation levels of specific cell types. However, global methylation levels result from diverse and complex patterns of locus-specific methylation statuses, which can be responsible for the functional states of genes. At the locus-specific level, different theoretical approaches can address different developmental and evolutionary questions related to, for example, locus-specific methylation dynamics throughout development, mechanisms of enzymatic recognition of DNA sequences that need to be (de-)methylated, long-term fitness consequences of methylation mistakes in loci with different functions, and the evolutionary consequences of disturbances in locus-specific methylation dynamics during MZT. New theoretical approaches can be built on the current model in order to address more specific questions.

Finally, the proposed model is the first attempt to look at the maternal-to-zygotic transition from a theoretical perspective. Although it lacks a complete description of the mechanisms involved in methylation dynamics, it provides an insight into the possible consequences of the mixing of homologous genomes with different global methylation levels and the mechanisms involved in the transition from a mixed methylome into a sex-specific (or tissue-specific) methylome. Future experimental studies will elucidate the molecular mechanisms involved in the methylome transition and the processes responsible for the activation of the zygotic genome. With that, accurate models of the system will be able to answer more detailed developmental and evolutionary questions.

## Supporting information

S1 File R script for model simulations and figures. (PDF)
