# Supplementary material for: Methylation dynamics during the maternal-to-zygotic genome transition in dioecious species
Source: PLoS One. 2018 Jul 10;13(7):e0200028. doi: 10.1371/journal.pone.0200028 (PMC6039002; doi:10.1371/journal.pone.0200028)
Supplement: S1 File — (PDF) [file pone.0200028.s001.pdf]

Code ▾

Modeling methylation dynamics during the maternal-to-zygotic transition in dioecious species.

Willian Silva

Model

Concepts

This figure shows the types of methylation states that can be possibly found in a genome (A), the rates of the processes that are considered in the model (B) and the sequence of processes that take place during cell divisions (C).

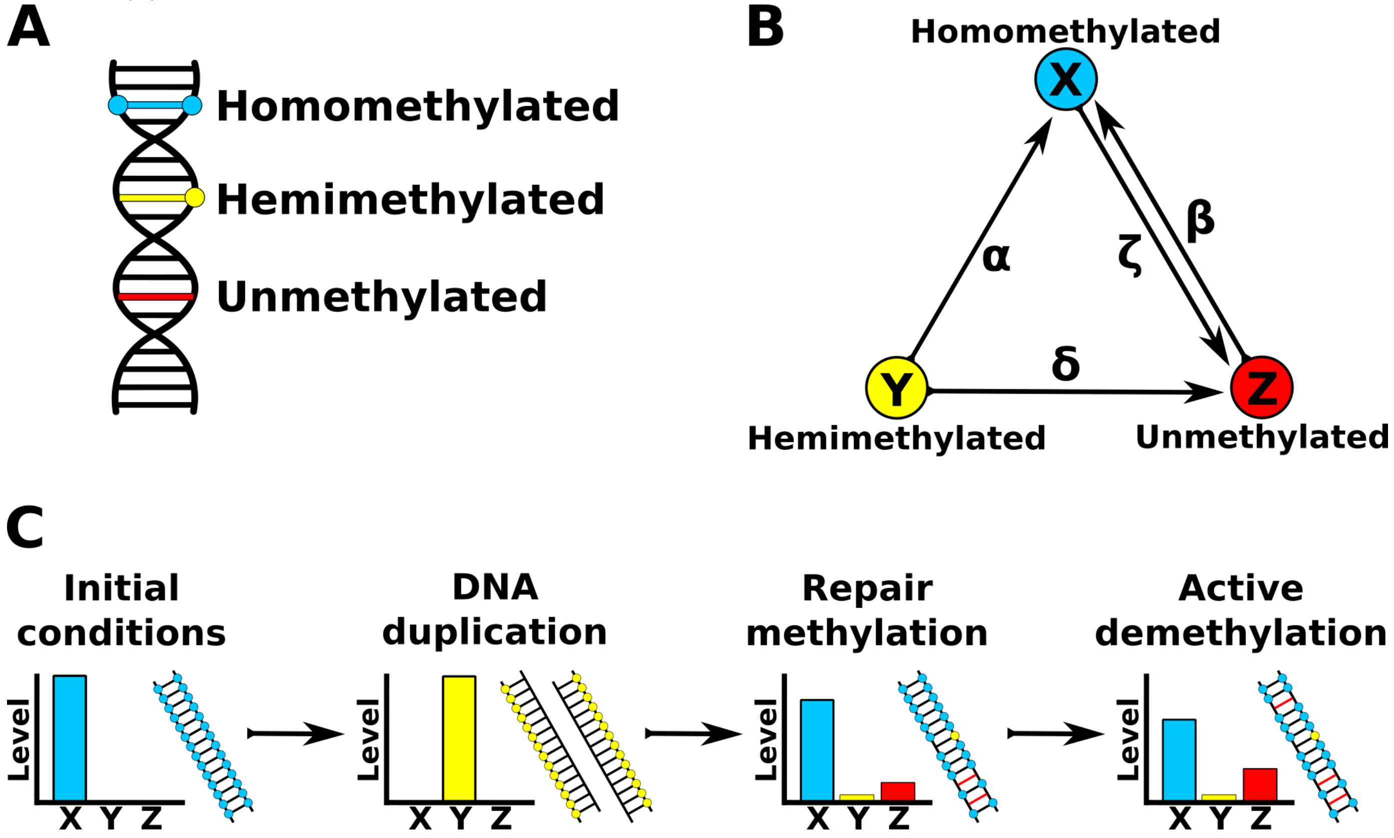

Set parameter values

Hide

```
seed<-777 #Seed for random variation in alpha and delta.
```

```
Warning messages:
1: In scan(file = file, what = what, sep = sep, quote = quote, dec = dec,  :
  EOF within quoted string
2: In scan(file = file, what = what, sep = sep, quote = quote, dec = dec,  :
  EOF within quoted string
```

Hide

```
ngen<-10 #Number of generations.
ndiv<-250 #Number of cell divisions per generation.
r<-0.1 #Intrinsic cell division rate.
K<-1024 #Number of cells at developmental equilibrium.
rho0<-1.0 #Initial amount of maternal repressor per generation (as a proportion of
 r).
rhod<-0.1 #Maternal repressor degradation rate.
nuthreshold<-1/100 #Nucleocytoplasmic ration at which the ZGA takes place.
rhotrigger<-"T" #Set the level of rho as the trigger of ZGA instead of nu.
rhofactor<-1 #Scaling factor of rho for triggering ZGA.
Xm0<-1.0 #Initial proportion of homomethylated sites in males.
Ym0<-0 #Initial proportion of hemimethylated sites in males.
Zm0<-0 #Initial proportion of unmethylated sites in males.
Xf0<-1 #Initial proportion of homomethylated sites in females.
Yf0<-0 #Initial proportion of hemimethylated sites in females.
Zf0<-0 #Initial proportion of unmethylated sites in females.
alpham<-0.99 #Alpha in males.
betam<-0.1 #Beta in males.
deltam<-0.01 #Delta in males.
zetam<-0 #Zeta in males.
alphaf<-0.99 #Alpha in females.
betaf<-0.04 #Beta in females.
deltaf<-0.01 #Delta in females.
zetaf<-0 #Zeta in females.
gendist<-"F" #Frequent disturbances between generations.
genfreq<-25 #Frequency of disturbances between generations (in number of generation
s).
alphamin<--0.01 #Minimum of addition to alpha during disturbances.
alphamax<-0.01 #Maximum of addition to alpha during disturbances.
deltamin<--0.01 #Minimum of addition to delta during disturbances.
deltamax<-0.01 #Maximum of addition to delta during disturbances.
distmale<-"F" #Disturbances across generations in males.
distfemale<-"F" #Disturbances across generations in females.
devgen<-2 #Generation to be plotted.
accuracy<-2 #Accuracy of values (number of decimals).
kroneckermodel<-"F" #TRUE for kronecker (boolean) model and FALSE for Heaviside mode
l.
heavisideslope<-100 #Steepness of heaviside step function (high values for abrupt cha
nge) when kroneckermodel=FALSE.
parexplor<-"F" #Run parameter exploration again.
saveplots<-"F" #Save plots.
modelversion<-1 #Version of the model.
note<-"Male disturbance." #Scenario description.
```

Set saving options

Hide

```
#Create a folder for the simulations, if it doesn't exist yet.
system("mkdir -p article1-output")
#Saving files.
fileout<-format(Sys.time(), "simmet-%Y%m%d%H%M%S-") #Define name of new file based on
 current date and time.
#Save input values.
inputvalues=array(data=0,dim=c(2,40))
inputvalues[1,1:40]=c("modelversion","seed","ngen","ndiv","r","K","rho0","rhod","nuth
reshold","rhotrigger","rhofactor","Xm0","Ym0","Zm0","Xf0","Yf0","Zf0","alpham","beta
m","deltam","zetam","alphaf","betaf","deltaf","zetaf","alphamin","alphamax","deltami
n","deltamax","distmale","distfemale","accuracy","kroneckermodel","heavisideslope","p
arexplor","devgen","gendist","genfreq","saveplots","note")
inputvalues[2,1:40]=c(modelversion,seed,ngen,ndiv,r,K,rho0,rhod,nuthreshold,rhotrigge
r,rhofactor,Xm0,Ym0,Zm0,Xf0,Yf0,Zf0,alpham,betam,deltam,zetam,alphaf,betaf,deltaf,zet
af,alphamin,alphamax,deltamin,deltamax,distmale,distfemale,accuracy,kroneckermodel,he
avisideslope,parexplor,devgen,gendist,genfreq,saveplots,note)
if(saveplots=="T"|saveplots=="TRUE"){
 write(inputvalues,file=paste0("article1-output/",fileout,"inputvalues.txt"),ncolumns
=2,append=FALSE,sep="\t")
}
```

Create matrices

Hide

```
#Store values of X, Y and Z across cell divisions and generations.
Xm=array(data=0,dim=c(ngen,ndiv+1)); Xm[1,1]=Xm0
Ym=array(data=0,dim=c(ngen,ndiv+1)); Ym[1,1]=Ym0
Zm=array(data=0,dim=c(ngen,ndiv+1)); Zm[1,1]=Zm0
Xf=array(data=0,dim=c(ngen,ndiv+1)); Xf[1,1]=Xf0
Yf=array(data=0,dim=c(ngen,ndiv+1)); Yf[1,1]=Yf0
Zf=array(data=0,dim=c(ngen,ndiv+1)); Zf[1,1]=Zf0
#Gametes.
Xmg=array(data=0,dim=c(ngen))
Ymg=array(data=0,dim=c(ngen))
Zmg=array(data=0,dim=c(ngen))
Xfg=array(data=0,dim=c(ngen))
Yfg=array(data=0,dim=c(ngen))
Zfg=array(data=0,dim=c(ngen))
#Cell division dynamics.
ncells=array(data=0,dim=c(ngen,ndiv+1)); ncells[,1]=1
nullncells=array(data=0,dim=c(ndiv+1)); nullncells[1]=1
rho=array(data=0,dim=c(ngen,ndiv+1)); rho[,1]=rho0
mu=array(data=0,dim=c(ngen,ndiv+1)); mu[,1]=(1+rho0)*r
nu=array(data=0,dim=c(ngen,ndiv+1))

#Random changes in alpha and delta (environmental effects).
newalpham=array(data=0,dim=c(ngen)); newalpham[1]=alpham
newdeltam=array(data=0,dim=c(ngen)); newdeltam[1]=deltam
newalphaf=array(data=0,dim=c(ngen)); newalphaf[1]=alphaf
newdeltaf=array(data=0,dim=c(ngen)); newdeltaf[1]=deltaf
```

Methylation dynamics (Kronecker delta model)

Hide

```
if(kroneckermodel=="T"|kroneckermodel=="TRUE"){
  for (g in 1:ngen){
    for (d in 1:ndiv){
      #Cell division dynamics.
      if (g==1){
        nullncells[d+1]=nullncells[d]+r*nullncells[d]*(1-nullncells[d]/K)
      } #Null model (constant rate of division).

      mu[g,d]=(1+rho[g,d])*r #Rate of division.
      ncells[g,d+1]=ncells[g,d]+mu[g,d]*ncells[g,d]*(1-ncells[g,d]/K) #Number of cell
s.
      nu[g,d]=1/ncells[g,d] #Nucleocytoplasmic ratio.
      rho[g,d+1]=rho[g,d]-rhod*rho[g,d] #Amount of maternal repressor.

      #Calculate rate of division and nucleocytoplasmic ratio of last developmental d
ivision.
      if (d==ndiv){
        mu[g,d+1]=(1+rho[g,d+1])*r
        nu[g,d+1]=1/ncells[g,d+1]
      }

      #Calculate the impact of rho (rhoc) on methylation dynamics when ZGA is trigger
ed.
      if (rho0>0){
        rhoc=(rho0-rho[g,d])/rho0
      }else{
        rhoc=1
      }

      if (nu[g,d]>nuthreshold){
        Xm[g,d+1]=Xm[g,d]; Ym[g,d+1]=Ym[g,d]; Zm[g,d+1]=Zm[g,d]
        Xf[g,d+1]=Xf[g,d]; Yf[g,d+1]=Yf[g,d]; Zf[g,d+1]=Zf[g,d]
      }else{

        #Instead of using this threshold function, it's more realistic to use the ana
lytic approximation of the heaviside step function (https://en.wikipedia.org/wiki/Hea
viside_step_function).
        #heaviside01<-function(steepness,nuthreshold,div,gofrom,goto){
        #  (goto-gofrom)/(1+exp(-steepness*(div-nuthreshold)))+gofrom
        #}
        #plot(heaviside01(5,50,0:200,0.0,0.5),type="l",lty=1,ylim=c(0,1.0),xlab="Cell
 divisions",ylab="Alpha",col="red")
        #par(new=TRUE)
        #plot(heaviside01(5,100,0:200,0.3,0.7),type="l",lty=3,ylim=c(0,1.0),xlab=NA,y
lab=NA,col="red")
        #par(new=TRUE)
        #plot(heaviside01(5,50,0:200,0.9,0.3),type="l",lty=1,ylim=c(0,1.0),xlab="Cell
 divisions",ylab="Alpha",col="blue")
        #par(new=TRUE)
        #plot(heaviside01(5,100,0:200,0.6,0.0),type="l",lty=3,ylim=c(0,1.0),xlab=NA,y
lab=NA,col="blue")
        #Methylation levels after DNA duplication.
        Xmd=0/2
        Ymd=(2*Xm[g,d]+Ym[g,d])/2
        Zmd=(2*Zm[g,d]+Ym[g,d])/2
        Xfd=0/2
        Yfd=(2*Xf[g,d]+Yf[g,d])/2
```

```
        Zfd=(2*Zf[g,d]+Yf[g,d])/2

        #Methylation levels after methylation repair and delay due to maternal effec
t.
        Xmr=alpham*Ymd+betam*Zmd
        Ymr=(1-alpham-rhoc*deltam)*Ymd
        Zmr=(1-betam)*Zmd+rhoc*deltam*Ymd
        Xfr=alphaf*Yfd+betaf*Zfd
        Yfr=(1-alphaf-rhoc*deltaf)*Yfd
        Zfr=(1-betaf)*Zfd+rhoc*deltaf*Yfd

        #Methylation levels after active demethylation.
        Xmm=(1-zetam)*Xmr
        Ymm=Ymr
        Zmm=Zmr+zetam*Xmr
        Xfm=(1-zetaf)*Xfr
        Yfm=Yfr
        Zfm=Zfr+zetaf*Xfr

        Xm[g,d+1]=Xmm
        Ym[g,d+1]=Ymm
        Zm[g,d+1]=Zmm
        Xf[g,d+1]=Xfm
        Yf[g,d+1]=Yfm
        Zf[g,d+1]=Zfm

        if (d==ndiv){
          #Gametes.
          Xmg[g]=Xm[g,d+1]
          Ymg[g]=Ym[g,d+1]
          Zmg[g]=Zm[g,d+1]
          Xfg[g]=Xf[g,d+1]
          Yfg[g]=Yf[g,d+1]
          Zfg[g]=Zf[g,d+1]

          if(g<ngen){
            #Fertilization.
            Xm[g+1,1]=(Xmg[g]+Xfg[g])/2
            Ym[g+1,1]=(Ymg[g]+Yfg[g])/2
            Zm[g+1,1]=(Zmg[g]+Zfg[g])/2
            Xf[g+1,1]=(Xmg[g]+Xfg[g])/2
            Yf[g+1,1]=(Ymg[g]+Yfg[g])/2
            Zf[g+1,1]=(Zmg[g]+Zfg[g])/2

            #Random changes in alpha and delta in the next generation.
            set.seed(seed)
            if (gendist=="T"){
              if (distmale=="T"){
                newalpham[g+1]=newalpham[g]+runif(1,alphamin,alphamax)
                newdeltam[g+1]=newdeltam[g]+runif(1,deltamin,deltamax)
                while ((newalpham[g+1]+newdeltam[g+1]>1) || (newalpham[g+1]<0) || (ne
wdeltam[g+1]<0)){
                  newalpham[g+1]=newalpham[g]+runif(1,alphamin,alphamax)
                  newdeltam[g+1]=newdeltam[g]+runif(1,deltamin,deltamax)}
                alpham=newalpham[g+1]
                deltam=newdeltam[g+1]
              }
              if (distfemale=="T"){
```

```
                newalphaf[g+1]=newalphaf[g]+runif(1,alphamin,alphamax)
                newdeltaf[g+1]=newdeltaf[g]+runif(1,deltamin,deltamax)
                while ((newalphaf[g+1]+newdeltaf[g+1]>1) || (newalphaf[g+1]<0) || (ne
wdeltaf[g+1]<0)){
                 newalphaf[g+1]=newalphaf[g]+runif(1,alphamin,alphamax)
                  newdeltaf[g+1]=newdeltaf[g]+runif(1,deltamin,deltamax)}
                alphaf=newalphaf[g+1]
                deltaf=newdeltaf[g+1]
              }
            }
          }
        }
      }
    }
  }
}
```

Methylation dynamics (Heaviside model)

This model assumes that alpha=1.0, beta=0.0, delta=0.0 and zeta=0.0 before ZGA. As a consequence, hemimethylated sites are transitional and will be turned into homomethylated sites because of alpha=1.0.

Hide

```
if(kroneckermodel=="F"|kroneckermodel=="FALSE"){

  #Heaviside step Functions.
  heaviside01<-function(steepness,nuthreshold,div,gofrom,goto){
    ((goto-gofrom)/(1+exp(-steepness*(div-nuthreshold))))+gofrom
  } #This functions steps up (0 to 1) or down (1 to 0).

  for (g in 1:ngen){
    for (d in 1:ndiv){
      #Cell division dynamics.
      if (g==1){
        nullncells[d+1]=nullncells[d]+r*nullncells[d]*(1-nullncells[d]/K)
      } #Null model (constant rate of division).

      mu[g,d]=(1+rho[g,d])*r #Rate of division.
      ncells[g,d+1]=ncells[g,d]+mu[g,d]*ncells[g,d]*(1-ncells[g,d]/K) #Number of cell
s.
      nu[g,d]=1/ncells[g,d] #Nucleocytoplasmic ratio.
      rho[g,d+1]=rho[g,d]-rhod*rho[g,d] #Amount of maternal repressor.

      #Calculate rate of division and nucleocytoplasmic ratio of last developmental d
ivision.
      if (d==ndiv){
        mu[g,d+1]=(1+rho[g,d+1])*r
        nu[g,d+1]=1/ncells[g,d+1]
      }

      #Calculate the impact of rho (rhoc) on methylation dynamics when ZGA is trigger
ed.
      if (rho0>0){
        rhoc=(rho0-rho[g,d])/rho0
      }else{
        rhoc=1
      }

      halpham<-heaviside01(heavisideslope,(nuthreshold)^(-1),ncells[g,d],1.0,alpham)
      hbetam<-heaviside01(heavisideslope,(nuthreshold)^(-1),ncells[g,d],0.0,betam)
      hdeltam<-heaviside01(heavisideslope,(nuthreshold)^(-1),ncells[g,d],0.0,deltam)
      hzetam<-heaviside01(heavisideslope,(nuthreshold)^(-1),ncells[g,d],0.0,zetam)

      halphaf<-heaviside01(heavisideslope,(nuthreshold)^(-1),ncells[g,d],1.0,alphaf)
      hbetaf<-heaviside01(heavisideslope,(nuthreshold)^(-1),ncells[g,d],0.0,betaf)
      hdeltaf<-heaviside01(heavisideslope,(nuthreshold)^(-1),ncells[g,d],0.0,deltaf)
      hzetaf<-heaviside01(heavisideslope,(nuthreshold)^(-1),ncells[g,d],0.0,zetaf)

      if(rhotrigger=="T"|rhotrigger=="TRUE"){
        #Using the maternal repressor as the initiator of methylation dynamics, rathe
r than the number of cells. rhofactoris a scaling factor so that rho can be in the sa
me scale as the number of cells.
        halpham<-heaviside01(heavisideslope,(nuthreshold)^(-1),rhofactor*rho0/rho[g,d
],1.0,alpham)
        hbetam<-heaviside01(heavisideslope,(nuthreshold)^(-1),rhofactor*rho0/rho[g,d]
,0.0,betam)
        hdeltam<-heaviside01(heavisideslope,(nuthreshold)^(-1),rhofactor*rho0/rho[g,d
],0.0,deltam)
        hzetam<-heaviside01(heavisideslope,(nuthreshold)^(-1),rhofactor*rho0/rho[g,d]
,0.0,zetam)
```

```
        halphaf<-heaviside01(heavisideslope,(nuthreshold)^(-1),rhofactor*rho0/rho[g,d
],1.0,alphaf)
        hbetaf<-heaviside01(heavisideslope,(nuthreshold)^(-1),rhofactor*rho0/rho[g,d]
,0.0,betaf)
        hdeltaf<-heaviside01(heavisideslope,(nuthreshold)^(-1),rhofactor*rho0/rho[g,d
],0.0,deltaf)
        hzetaf<-heaviside01(heavisideslope,(nuthreshold)^(-1),rhofactor*rho0/rho[g,d]
,0.0,zetaf)
      }

      #heaviside01<-function(steepness,nuthreshold,div,gofrom,goto){
      #  (goto-gofrom)/(1+exp(-steepness*(div-nuthreshold)))+gofrom
      #}
      #plot(heaviside01(5,50,0:200,0.0,0.5),type="l",lty=1,ylim=c(0,1.0),xlab="Cell d
ivisions",ylab="Alpha",col="red")
      #par(new=TRUE)
      #plot(heaviside01(5,100,0:200,0.3,0.7),type="l",lty=3,ylim=c(0,1.0),xlab=NA,yla
b=NA,col="red")
      #par(new=TRUE)
      #plot(heaviside01(5,50,0:200,0.9,0.3),type="l",lty=1,ylim=c(0,1.0),xlab="Cell d
ivisions",ylab="Alpha",col="blue")
      #par(new=TRUE)
      #plot(heaviside01(5,100,0:200,0.6,0.0),type="l",lty=3,ylim=c(0,1.0),xlab=NA,yla
b=NA,col="blue")

      #Methylation levels after DNA duplication.
      Xmd<-0/2
      Ymd<-(2*Xm[g,d]+Ym[g,d])/2
      Zmd<-(2*Zm[g,d]+Ym[g,d])/2
      Xfd<-0/2
      Yfd<-(2*Xf[g,d]+Yf[g,d])/2
      Zfd<-(2*Zf[g,d]+Yf[g,d])/2

      #Methylation levels after methylation repair and delay due to maternal effect.
      Xmr<-halpham*Ymd+hbetam*Zmd
      Ymr<-(1-halpham-rhoc*hdeltam)*Ymd
      Zmr<-(1-hbetam)*Zmd+rhoc*hdeltam*Ymd
      Xfr<-halphaf*Yfd+hbetaf*Zfd
      Yfr<-(1-halphaf-rhoc*hdeltaf)*Yfd
      Zfr<-(1-hbetaf)*Zfd+rhoc*hdeltaf*Yfd

      #Methylation levels after active demethylation.
      Xmm<-(1-hzetam)*Xmr
      Ymm<-Ymr
      Zmm<-Zmr+hzetam*Xmr
      Xfm<-(1-hzetaf)*Xfr
      Yfm<-Yfr
      Zfm<-Zfr+hzetaf*Xfr

      Xm[g,d+1]<-Xmm
      Ym[g,d+1]<-Ymm
      Zm[g,d+1]<-Zmm
      Xf[g,d+1]<-Xfm
      Yf[g,d+1]<-Yfm
      Zf[g,d+1]<-Zfm

      if (d==ndiv){
```

```
        #Gametes.
        Xmg[g]<-Xm[g,d+1]
        Ymg[g]<-Ym[g,d+1]
        Zmg[g]<-Zm[g,d+1]
        Xfg[g]<-Xf[g,d+1]
        Yfg[g]<-Yf[g,d+1]
        Zfg[g]<-Zf[g,d+1]

        if(g<ngen){
          #Fertilization.
          Xm[g+1,1]<-(Xmg[g]+Xfg[g])/2
          Ym[g+1,1]<-(Ymg[g]+Yfg[g])/2
          Zm[g+1,1]<-(Zmg[g]+Zfg[g])/2
          Xf[g+1,1]<-(Xmg[g]+Xfg[g])/2
          Yf[g+1,1]<-(Ymg[g]+Yfg[g])/2
          Zf[g+1,1]<-(Zmg[g]+Zfg[g])/2

          #Random changes in alpha and delta in the next generation.
          set.seed(seed)
          if (gendist=="T"){
            if (distmale=="T"){
              newalpham[g+1]<-newalpham[g]+runif(1,alphamin,alphamax)
              newdeltam[g+1]<-newdeltam[g]+runif(1,deltamin,deltamax)
              while ((newalpham[g+1]+newdeltam[g+1]>1) || (newalpham[g+1]<0) || (newd
eltam[g+1]<0)){
                newalpham[g+1]<-newalpham[g]+runif(1,alphamin,alphamax)
                newdeltam[g+1]<-newdeltam[g]+runif(1,deltamin,deltamax)}
              alpham<-newalpham[g+1]
              deltam<-newdeltam[g+1]
            }
            if (distfemale=="T"){
              newalphaf[g+1]<-newalphaf[g]+runif(1,alphamin,alphamax)
              newdeltaf[g+1]<-newdeltaf[g]+runif(1,deltamin,deltamax)
              while ((newalphaf[g+1]+newdeltaf[g+1]>1) || (newalphaf[g+1]<0) || (newd
eltaf[g+1]<0)){
                newalphaf[g+1]<-newalphaf[g]+runif(1,alphamin,alphamax)
                newdeltaf[g+1]<-newdeltaf[g]+runif(1,deltamin,deltamax)}
              alphaf<-newalphaf[g+1]
              deltaf<-newdeltaf[g+1]
            }
          }
        }
      }
    }
  }
}
```

Equilibrium points

Hide

```
#Number of cell divisions to reach the equilibrium number of cells in the null model.
eqnullcells<-round(nullncells[ndiv+1]) ; eqnullcells #Number of cells.
```

```
[1] 1024
```

Hide

```
eqnulldivcells<-sum(round(nullncells[],digits=0)!=K) ; eqnulldivcells #Number of divi
sions.
```

```
[1] 146
```

Hide

```
#Generation 1.
#Final number of cells (and probably equilibrium) during generation 1.
eqcells1<-round(ncells[1,ndiv+1]) ; eqcells1 #Repressor model.
```

```
[1] 1024
```

Hide

```
#Number of cell divisions to reach the equilibrium number of cells during generation
 1.
eqdivcells1<-sum(round(ncells[1,],digits=0)!=K) ; eqdivcells1 #Repressor model.
```

```
[1] 136
```

Hide

```
#Final X value during generation 1 (and probably equilibrium value).
eqXm1<-round(Xm[1,ndiv+1],digits=2) ; eqXm1 #Males.
```

```
[1] 0.91
```

Hide

```
eqXf1<-round(Xf[1,ndiv+1],digits=2) ; eqXf1 #Females.
```

```
[1] 0.8
```

Hide

```
#Number of cell divisions necessary to reach the expected methylation level during ge
neration 1.
eqdivXm1<-sum(round(Xm[1,],digits=2)!=eqXm1) ; eqdivXm1 #Males.
```

```
[1] 68
```

Hide

```
eqdivXf1<-sum(round(Xf[1,],digits=2)!=eqXf1) ; eqdivXf1 #Females.
```

```
[1] 117
```

Hide

```
#Generation 2.
#Final number of cells (and probably equilibrium) during generation 1.
eqcells2<-round(ncells[2,ndiv+1]) ; eqcells2 #Repressor model.
```

```
[1] 1024
```

Hide

```
#Number of cell divisions to reach the equilibrium number of cells during generation
 2.
eqdivcells2<-sum(round(ncells[2,],digits=0)!=K) ; eqdivcells2 #Repressor model.
```

```
[1] 136
```

Hide

```
#Final X value during generation 2 (and probably equilibrium value).
eqXm2<-round(Xm[2,ndiv+1],digits=2) ; eqXm2 #Males.
```

```
[1] 0.91
```

Hide

```
eqXf2<-round(Xf[2,ndiv+1],digits=2) ; eqXf2 #Females.
```

```
[1] 0.8
```

Hide

```
#Number of cell divisions necessary to reach the expected methylation level during ge
neration 2.
eqdivXm2<-sum(round(Xm[2,],digits=2)!=eqXm2) ; eqdivXm2 #Males.
```

```
[1] 67
```

Hide

```
eqdivXf2<-sum(round(Xf[2,],digits=2)!=eqXf2) ; eqdivXf2 #Females.
```

```
[1] 91
```

Hide

```
#Generation devgen.
#Final number of cells (and probably equilibrium) during generation 1.
eqcellsdevgen<-round(ncells[devgen,ndiv+1]) ; eqcellsdevgen #Repressor model.
```

```
[1] 1024
```

Hide

```
#Number of cell divisions to reach the equilibrium number of cells during generation devgen.
eqdivcellsdevgen<-sum(round(ncells[devgen,],digits=0)!=K) ; eqdivcellsdevgen #Repressor model.
```

```
[1] 136
```

Hide

```
#Final X value during generation devgen (and probably equilibrium value).
eqXmdevgen<-round(Xm[devgen,ndiv+1],digits=2) ; eqXmdevgen #Males.
```

```
[1] 0.91
```

Hide

```
eqXfdevgen<-round(Xf[devgen,ndiv+1],digits=2) ; eqXfdevgen #Females.
```

```
[1] 0.8
```

Hide

```
#Number of cell divisions necessary to reach the expected methylation level during generation devgen.
eqdivXmdevgen<-sum(round(Xm[devgen,],digits=2)!=eqXmdevgen) ; eqdivXmdevgen #Males.
```

```
[1] 67
```

Hide

```
eqdivXfdevgen<-sum(round(Xf[devgen,],digits=2)!=eqXfdevgen) ; eqdivXfdevgen #Females.
```

```
[1] 91
```

Plots

Plot 1

This plot shows the comparison of cell division dynamics between a simulation with a maternal repressor speeding up cell divisions and a simulation with constant intrinsic cell division rate. Vertical bars mark the cell division at which the number of cells reach the specified equilibrium.

Hide

```
if(saveplots=="T"|saveplots=="TRUE"){
 pdf(file=paste0("article1-output/",fileout,"plot1.pdf"),width=30,height=20,pointsize
=30)
}
pointsize<-1.5 #Size of text.
linethickness<-4 #Line thickness.
minx<-0 #Minimum of x-axis.
maxx<-ndiv #Maximum of x-axis.
miny<-0 #Minimum of y-axis.
maxy<-1.01*K #Maximum of y-axis.
par(xpd=T,mar=par()$mar+c(0,1,0,6))
plot(ncells[devgen,],type="l",lty=1,lwd=linethickness,col="blue",main="Cell number dy
namics",xlab="Cell divisions",ylab="Number of cells",xlim=c(minx,maxx), ylim=c(miny,m
axy),cex.axis=pointsize,cex.lab=pointsize,cex.main=pointsize)
par(new=T)
```

Hide

```
plot(nullncells[],type="l",lty=1,lwd=linethickness,col="red",main=NA,xlab=NA,ylab=NA,
xlim=c(minx,maxx), ylim=c(miny,maxy),cex.axis=pointsize,cex.lab=pointsize,cex.main=po
intsize)
polygon(x=c(eqdivcellsdevgen,eqdivcellsdevgen),y=c(eqcellsdevgen-0.03*eqcellsdevgen,e
qcellsdevgen+0.03*eqcellsdevgen),lty=1,lwd=linethickness,border="blue")
```

Hide

```
polygon(x=c(eqnulldivcells,eqnulldivcells),y=c(eqnullcells-0.03*eqnullcells,eqnullcel
ls+0.03*eqnullcells),lty=1,lwd=linethickness,border="red")
legend(maxx+0.08*maxx,maxy,c(paste0("Rho0=",rho0),"Rho0=0"),lty=c(1,1),lwd=linethickn
ess,col=c("blue","red"))
```

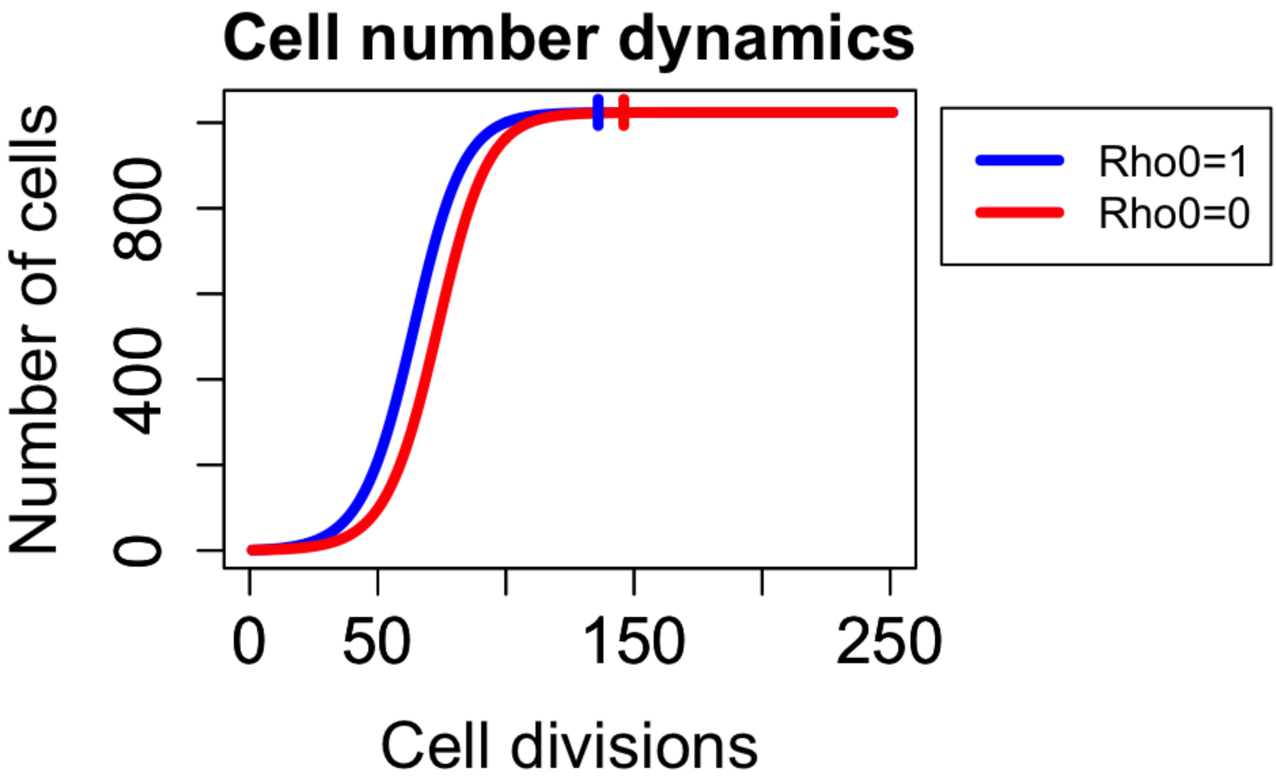

Hide

```
if(saveplots=="T"|saveplots=="TRUE"){
 dev.off()
}
```

Plot 2

This plot shows the change in maternal repressor across cell divisions (rho) and the consequent change in cell division speed (mu).

Hide

```
if(saveplots=="T"|saveplots=="TRUE"){
 pdf(file=paste0("article1-output/",fileout,"plot2.pdf"),width=30,height=20,pointsize
=30)
}
pointsize<-1.5 #Size of text.
linethickness<-4 #Line thickness.
minx<-0 #Minimum of x-axis.
maxx<-ndiv #Maximum of x-axis.
miny<-0 #Minimum of y-axis.
maxy<-rho0 #Maximum of x-axis.
par(xpd=T,mar=par()$mar+c(0,1,0,5))
plot(rho[devgen,],type="l",lty=1,lwd=linethickness,col="blue",main="Decay of rho and
 cell division rate",xlab="Cell divisions",ylab="Rho, Mu",xlim=c(minx,maxx), ylim=c(m
iny,maxy),cex.axis=pointsize,cex.lab=pointsize,cex.main=pointsize)
par(new=T)
```

Hide

```
plot(mu[devgen,],type="l",lty=1,lwd=linethickness,col="red",main=NA,xlab=NA,ylab=NA,x
lim=c(minx,maxx), ylim=c(miny,maxy),cex.axis=pointsize,cex.lab=pointsize,cex.main=poi
ntsize)
legend(maxx+0.08*(maxx),maxy,c("Rho","Mu"),lty=c(1,1),lwd=linethickness,col=c("blue",
"red"))
```

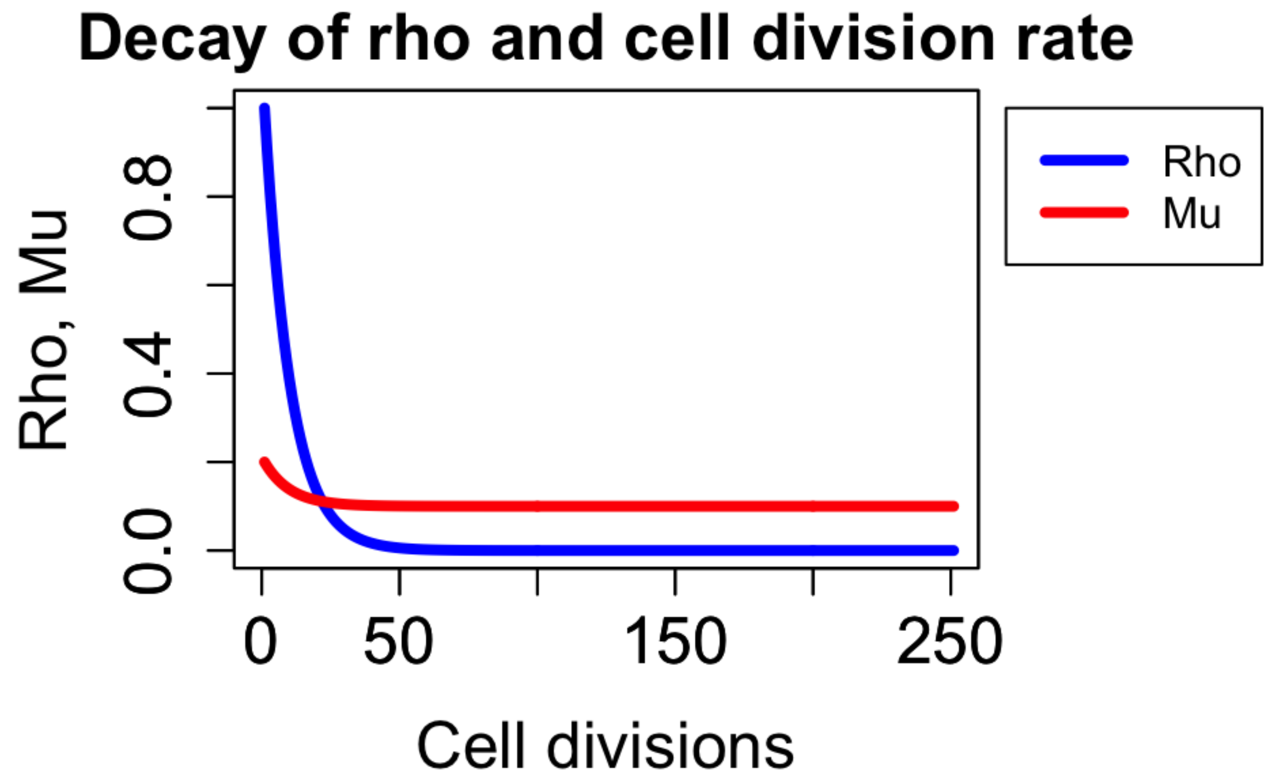

Hide

```
if(saveplots=="T"|saveplots=="TRUE"){
 dev.off()
}
```

Plot 3

This plot shows the methylation dynamics during the first generation, in which the initial proportion of homomethylated sites reach an equilibrium marked by the vertical bars.

Hide

```
if(saveplots=="T"|saveplots=="TRUE"){
 pdf(file=paste0("article1-output/",fileout,"plot3.pdf"),width=30,height=20,pointsize
=30)
}
pointsize<-1.5 #Size of text.
linethickness<-4 #Line thickness.
minx<-0 #Minimum of x-axis.
maxx<-ndiv #Maximum of x-axis.
miny<-0 #Minimum of y-axis.
maxy<-1.0 #Maximum of x-axis.
par(xpd=T,mar=par()$mar+c(0,1,0,6))
plot(Xm[1,],type="l",lty=1,lwd=linethickness,col="blue",main="Methylation dynamics (g
eneration #1)",xlab="Cell divisions",ylab="X, Y, Z",xlim=c(minx,maxx),ylim=c(miny,max
y),cex.axis=pointsize,cex.lab=pointsize,cex.main=pointsize)
par(new=T)
```

Hide

```
plot(Zm[1,],type="l",lty=1,lwd=linethickness,col="red",main=NA,xlab=NA,ylab=NA,xlim=c
(minx,maxx),ylim=c(miny,maxy),cex.axis=pointsize,cex.lab=pointsize,cex.main=pointsize
)
par(new=T)
```

Hide

```
plot(Ym[1,],type="l",lty=1,lwd=linethickness,col="green",main=NA,xlab=NA,ylab=NA,xlim
=c(minx,maxx),ylim=c(miny,maxy),cex.axis=pointsize,cex.lab=pointsize,cex.main=pointsi
ze)
par(new=T)
```

Hide

```
plot(Xf[1,],type="l",lty=3,lwd=linethickness,col="blue",main=NA,xlab=NA,ylab=NA,xlim=
c(minx,maxx),ylim=c(miny,maxy),cex.axis=pointsize,cex.lab=pointsize,cex.main=pointsiz
e)
par(new=T)
```

Hide

```
plot(Zf[1,],type="l",lty=3,lwd=linethickness,col="red",main=NA,xlab=NA,ylab=NA,xlim=c
(minx,maxx),ylim=c(miny,maxy),cex.axis=pointsize,cex.lab=pointsize,cex.main=pointsize
)
par(new=T)
```

Hide

```
plot(Yf[1,],type="l",lty=3,lwd=linethickness,col="green",main=NA,xlab=NA,ylab=NA,xlim
=c(minx,maxx),ylim=c(miny,maxy),cex.axis=pointsize,cex.lab=pointsize,cex.main=pointsi
ze)
polygon(x=c(eqdivXm1,eqdivXm1),y=c(eqXm1-0.03,eqXm1+0.03),lty=1,lwd=linethickness,bor
der="purple")
```

Hide

```
polygon(x=c(eqdivXf1,eqdivXf1),y=c(eqXf1-0.03,eqXf1+0.03),lty=1,lwd=linethickness,bor
der="purple")
legend(maxx+0.1*(maxx),maxy,c("Male","Female","X","Y","Z","Xeq"),lty=c(1,3,1,1,1,1),l
wd=linethickness,col=c("black","black","blue","red","green","purple"))
```

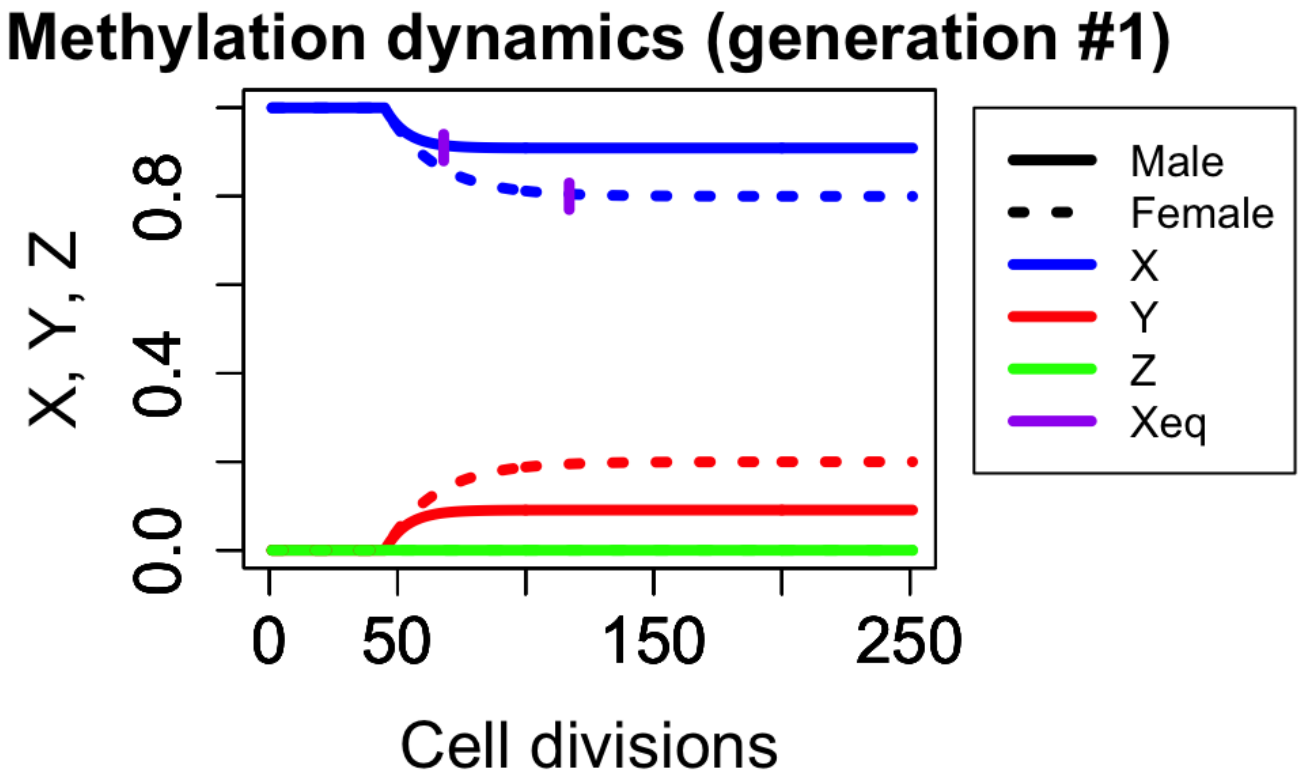

Hide

```
if(saveplots=="T"|saveplots=="TRUE"){
 dev.off()
}
```

Plot 4

This plot shows the methylation dynamics during generation devgen, in which the proportion of homomethylated sites starts at the average of the equilibria of the sexes at the previous generation. Vertical bars mark the cell division at which the equilibrium is reached.

Hide

```
if(saveplots=="T"|saveplots=="TRUE"){
 pdf(file=paste0("article1-output/",fileout,"plot4.pdf"),width=30,height=20,pointsize
=30)
}
pointsize<-1.5 #Size of text.
linethickness<-4 #Line thickness.
minx<-0 #Minimum of x-axis.
maxx<-ndiv #Maximum of x-axis.
miny<-0 #Minimum of y-axis.
maxy<-1.0 #Maximum of x-axis.
par(xpd=T,mar=par()$mar+c(0,1,0,6))
plot(Xm[devgen,],type="l",lty=1,lwd=linethickness,col="blue",main=paste0("Methylation
 dynamics (generation #",devgen,")"),xlab="Cell divisions",ylab="X, Y, Z",xlim=c(minx
,maxx),ylim=c(miny,maxy),cex.axis=pointsize,cex.lab=pointsize,cex.main=pointsize)
par(new=T)
```

Hide

```
plot(Zm[devgen,],type="l",lty=1,lwd=linethickness,col="red",main=NA,xlab=NA,ylab=NA,x
lim=c(minx,maxx),ylim=c(miny,maxy),cex.axis=pointsize,cex.lab=pointsize,cex.main=poin
tsize)
par(new=T)
```

Hide

```
plot(Ym[devgen,],type="l",lty=1,lwd=linethickness,col="green",main=NA,xlab=NA,ylab=NA
,xlim=c(minx,maxx),ylim=c(miny,maxy),cex.axis=pointsize,cex.lab=pointsize,cex.main=po
intsize)
par(new=T)
```

Hide

```
plot(Xf[devgen,],type="l",lty=3,lwd=linethickness,col="blue",main=NA,xlab=NA,ylab=NA,
xlim=c(minx,maxx),ylim=c(miny,maxy),cex.axis=pointsize,cex.lab=pointsize,cex.main=poi
ntsize)
par(new=T)
```

Hide

```
plot(Zf[devgen,],type="l",lty=3,lwd=linethickness,col="red",main=NA,xlab=NA,ylab=NA,x
lim=c(minx,maxx),ylim=c(miny,maxy),cex.axis=pointsize,cex.lab=pointsize,cex.main=poin
tsize)
par(new=T)
```

Hide

```
plot(Yf[devgen,],type="l",lty=3,lwd=linethickness,col="green",main=NA,xlab=NA,ylab=NA
,xlim=c(minx,maxx),ylim=c(miny,maxy),cex.axis=pointsize,cex.lab=pointsize,cex.main=po
intsize)
polygon(x=c(eqdivXmdevgen,eqdivXmdevgen),y=c(eqXmdevgen-0.03,eqXmdevgen+0.03),lty=1,l
wd=linethickness,border="purple")
```

Hide

```
polygon(x=c(eqdivXfdevgen,eqdivXfdevgen),y=c(eqXfdevgen-0.03,eqXfdevgen+0.03),lty=1,l
wd=linethickness,border="purple")
legend(maxx+0.08*maxx,maxy,c("Male","Female","X","Y","Z","Xeq"),lty=c(1,3,1,1,1,1),lw
d=linethickness,col=c("black","black","blue","green","red","purple"))
```

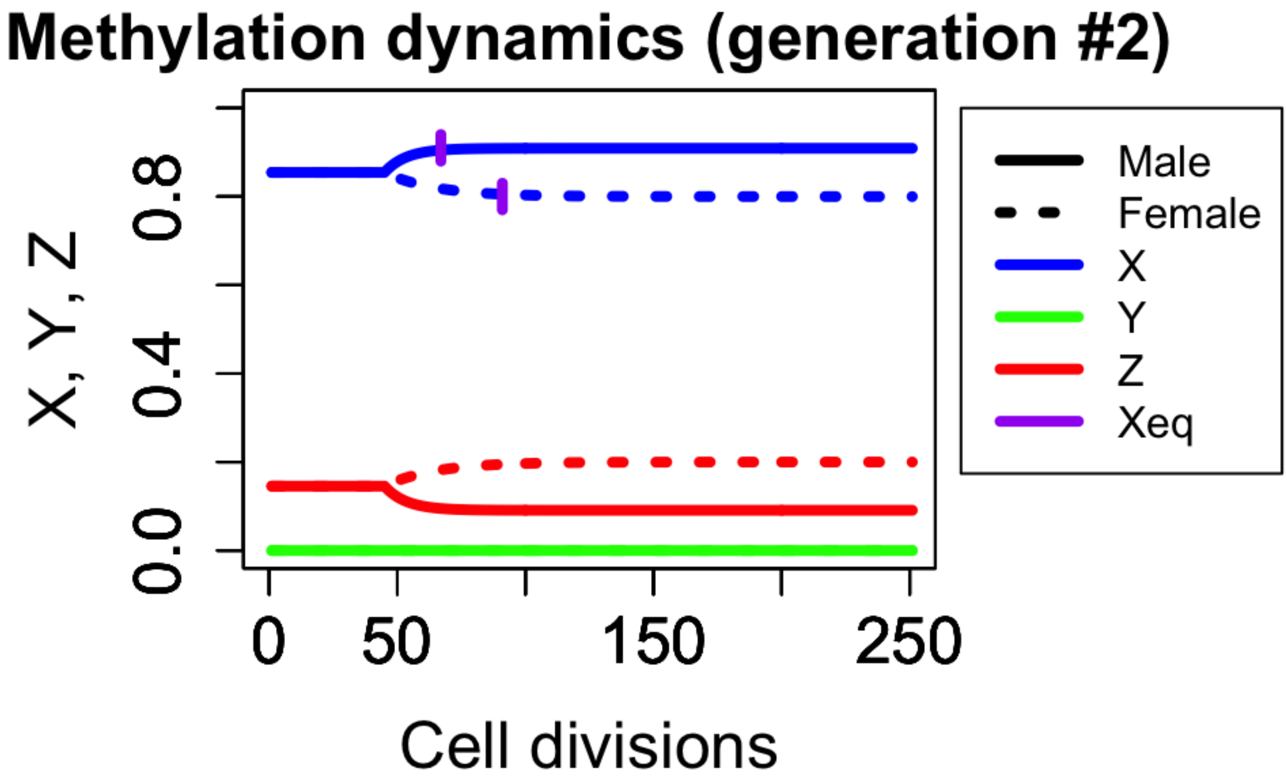

Hide

```
if(saveplots=="T"|saveplots=="TRUE"){
 dev.off()
}
```

Plot 5

This plot shows the methylation dynamics across ngen generations in males.

Hide

```
if(saveplots=="T"|saveplots=="TRUE"){
 pdf(file=paste0("article1-output/",fileout,"plot5.pdf"),width=30,height=20,pointsize
=30)
}
pointsize<-1.5 #Size of text.
linethickness<-4 #Line thickness.
minx<-0 #Minimum of x-axis.
maxx<-ngen*ndiv #Maximum of x-axis.
miny<-0 #Minimum of y-axis.
maxy<-1.0 #Maximum of x-axis.
par(xpd=T,mar=par()$mar+c(0,1,0,4))
plot(c(as.vector(t(Xm))),type="l",lty=1,lwd=4,col="blue",main="Methylation dynamics i
n males",xlab="Cell divisions",ylab="X, Y, Z",xlim=c(minx,maxx), ylim=c(miny,maxy),ce
x.axis=pointsize,cex.lab=pointsize,cex.main=pointsize)
par(new=T)
```

Hide

```
plot(c(as.vector(t(Zm))),type="l",lty=1,lwd=linethickness,col="red",main=NA,xlab=NA,y
lab=NA,xlim=c(minx,maxx), ylim=c(miny,maxy),cex.axis=pointsize,cex.lab=pointsize,cex.
main=pointsize)
par(new=T)
```

Hide

```
plot(c(as.vector(t(Ym))),type="l",lty=1,lwd=linethickness,col="green",main=NA,xlab=NA
,ylab=NA,xlim=c(minx,maxx), ylim=c(miny,maxy),cex.axis=pointsize,cex.lab=pointsize,ce
x.main=pointsize)
legend(maxx+0.08*maxx,maxy,c("X","Y","Z"),lty=1,lwd=linethickness,col=c("blue","gree
n","red"))
```

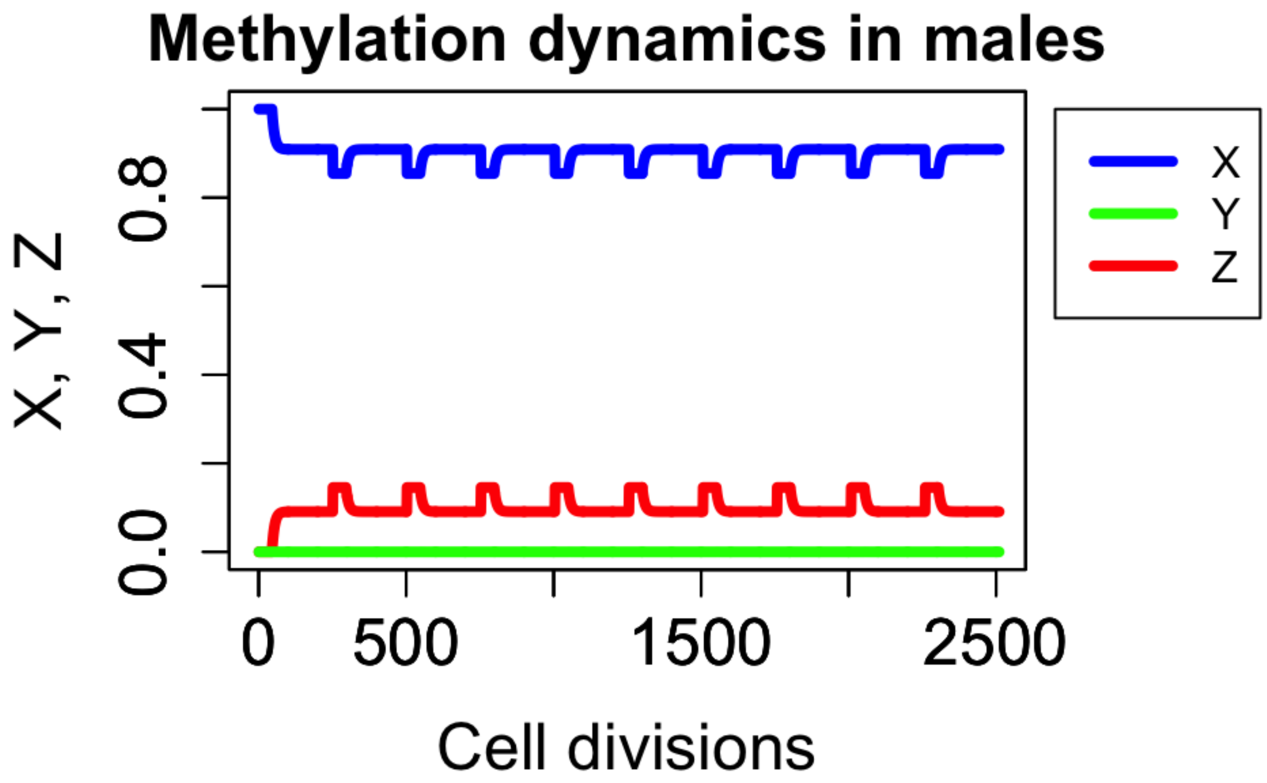

Hide

```
if(saveplots=="T"|saveplots=="TRUE"){
 dev.off()
}
```

Plot 6

This plot shows the methylation dynamics across ngen generations in females.

Hide

```
if(saveplots=="T"|saveplots=="TRUE"){
 pdf(file=paste0("article1-output/",fileout,"plot6.pdf"),width=30,height=20,pointsize
=30)
}
pointsize<-1.5 #Size of text.
linethickness<-4 #Line thickness.
minx<-0 #Minimum of x-axis.
maxx<-ngen*ndiv #Maximum of x-axis.
miny<-0 #Minimum of y-axis.
maxy<-1.0 #Maximum of x-axis.
par(xpd=T,mar=par()$mar+c(0,1,0,4))
plot(c(as.vector(t(Xf))),type="l",lty=1,lwd=4,col="blue",main="Methylation dynamics i
n females",xlab="Cell divisions",ylab="X, Y, Z",xlim=c(minx,maxx), ylim=c(miny,maxy),
cex.axis=pointsize,cex.lab=pointsize,cex.main=pointsize)
par(new=T)
```

Hide

```
plot(c(as.vector(t(Zf))),type="l",lty=1,lwd=linethickness,col="red",main=NA,xlab=NA,y
lab=NA,xlim=c(minx,maxx), ylim=c(miny,maxy),cex.axis=pointsize,cex.lab=pointsize,cex.
main=pointsize)
par(new=T)
```

Hide

```
plot(c(as.vector(t(Yf))),type="l",lty=1,lwd=linethickness,col="green",main=NA,xlab=NA
,ylab=NA,xlim=c(minx,maxx), ylim=c(miny,maxy),cex.axis=pointsize,cex.lab=pointsize,ce
x.main=pointsize)
legend(maxx+0.08*maxx,maxy,c("X","Y","Z"),lty=1,lwd=linethickness,col=c("blue","gree
n","red"))
```

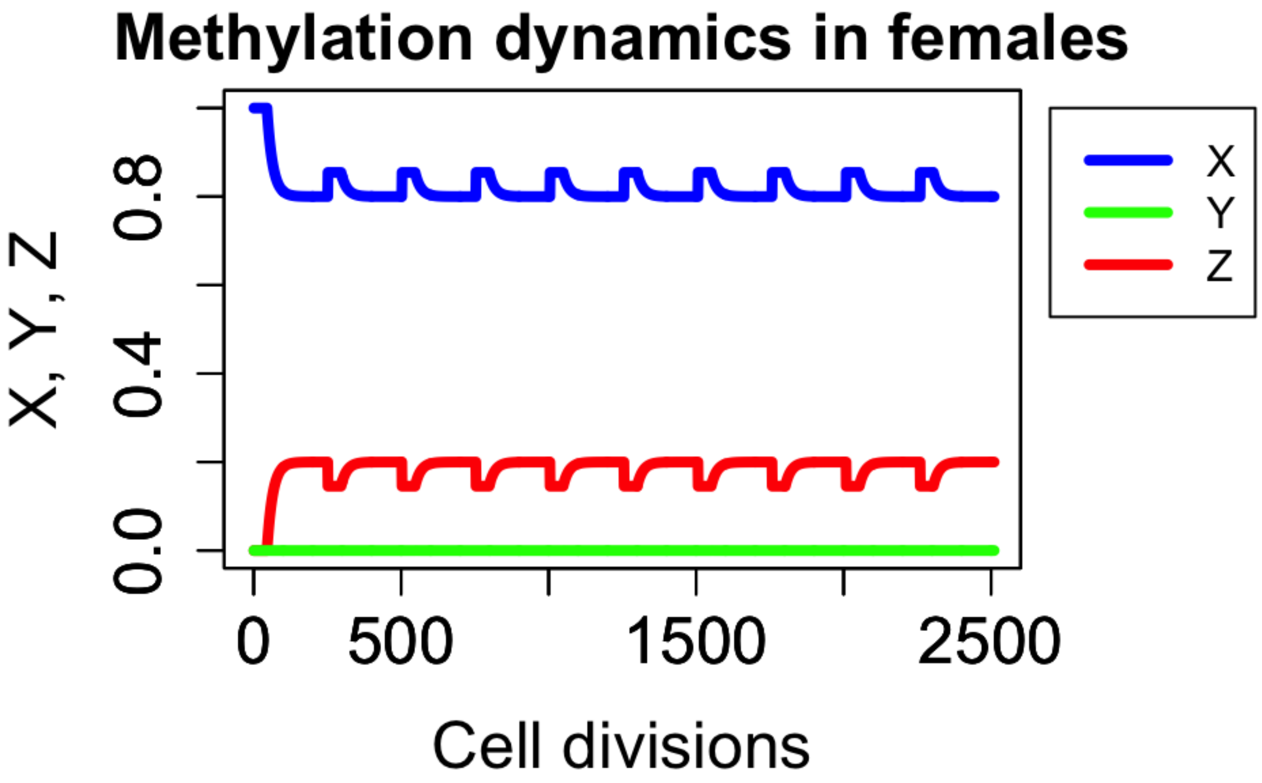

Hide

```
if(saveplots=="T"|saveplots=="TRUE"){
 dev.off()
}
```

Plot 7

This plot shows the methylation levels of the zygotes across ngen generations.

Hide

```
if(saveplots=="T"|saveplots=="TRUE"){
 pdf(file=paste0("article1-output/",fileout,"plot7.pdf"),width=30,height=20,pointsize
=30)
}
pointsize<-1.5 #Size of text.
linethickness<-4 #Line thickness.
minx<-1 #Minimum of x-axis.
maxx<-ngen #Maximum of x-axis.
miny<-0 #Minimum of y-axis.
maxy<-1.0 #Maximum of x-axis.
par(xpd=T,mar=par()$mar+c(0,1,0,4))
plot(Xm[,1],type="l",lty=1,lwd=4,col="blue",main="Methylation levels in zygotes acros
s generations",xlab="Generations",ylab="X, Y, Z",xlim=c(minx,maxx), ylim=c(miny,maxy)
,cex.axis=pointsize,cex.lab=pointsize,cex.main=pointsize)
par(new=T)
```

Hide

```
plot(Zm[,1],type="l",lty=1,lwd=linethickness,col="red",main=NA,xlab=NA,ylab=NA,xlim=c
(minx,maxx), ylim=c(miny,maxy),cex.axis=pointsize,cex.lab=pointsize,cex.main=pointsiz
e)
par(new=T)
```

Hide

```
plot(Ym[,1],type="l",lty=1,lwd=linethickness,col="green",main=NA,xlab=NA,ylab=NA,xlim
=c(minx,maxx), ylim=c(miny,maxy),cex.axis=pointsize,cex.lab=pointsize,cex.main=points
ize)
legend(maxx+0.08*maxx,maxy,c("X","Y","Z"),lty=1,lwd=linethickness,col=c("blue","gree
n","red"))
```

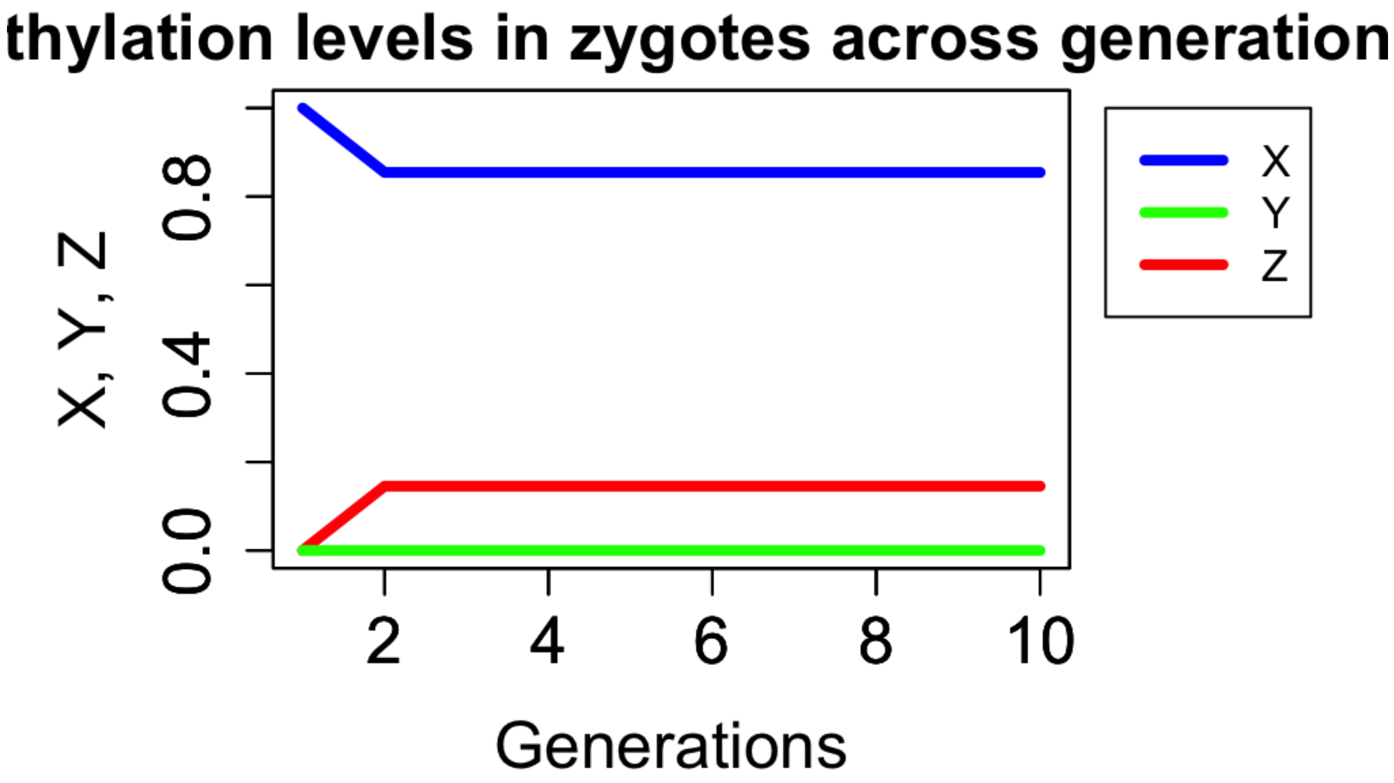

Hide

```
if(saveplots=="T"|saveplots=="TRUE"){
 dev.off()
}
```

Plot 8

This plot shows the methylation levels of the adults across ngen generations.

Hide

```
if(saveplots=="T"|saveplots=="TRUE"){
 pdf(file=paste0("article1-output/",fileout,"plot8.pdf"),width=30,height=20,pointsize
=30)
}
pointsize<-1.5 #Size of text.
linethickness<-4 #Line thickness.
minx<-1 #Minimum of x-axis.
maxx<-ngen #Maximum of x-axis.
miny<-0 #Minimum of y-axis.
maxy<-1.0 #Maximum of x-axis.
par(xpd=T,mar=par()$mar+c(0,1,0,6))
plot(Xm[,ndiv+1],type="l",lty=1,lwd=4,col="blue",main="Methylation levels in adults a
cross generations",xlab="Generations",ylab="X, Y, Z",xlim=c(minx,maxx), ylim=c(miny,m
axy),cex.axis=pointsize,cex.lab=pointsize,cex.main=pointsize)
par(new=T)
```

Hide

```
plot(Zm[,ndiv+1],type="l",lty=1,lwd=linethickness,col="red",main=NA,xlab=NA,ylab=NA,x
lim=c(minx,maxx), ylim=c(miny,maxy),cex.axis=pointsize,cex.lab=pointsize,cex.main=poi
ntsize)
par(new=T)
```

Hide

```
plot(Ym[,ndiv+1],type="l",lty=1,lwd=linethickness,col="green",main=NA,xlab=NA,ylab=NA
,xlim=c(minx,maxx), ylim=c(miny,maxy),cex.axis=pointsize,cex.lab=pointsize,cex.main=p
ointsize)
par(new=T)
```

Hide

```
plot(Xf[,ndiv+1],type="l",lty=3,lwd=linethickness,col="red",main=NA,xlab=NA,ylab=NA,x
lim=c(minx,maxx), ylim=c(miny,maxy),cex.axis=pointsize,cex.lab=pointsize,cex.main=poi
ntsize)
par(new=T)
```

Hide

```
plot(Zf[,ndiv+1],type="l",lty=3,lwd=linethickness,col="red",main=NA,xlab=NA,ylab=NA,x
lim=c(minx,maxx), ylim=c(miny,maxy),cex.axis=pointsize,cex.lab=pointsize,cex.main=poi
ntsize)
par(new=T)
```

Hide

```
plot(Yf[,ndiv+1],type="l",lty=3,lwd=linethickness,col="green",main=NA,xlab=NA,ylab=NA
,xlim=c(minx,maxx), ylim=c(miny,maxy),cex.axis=pointsize,cex.lab=pointsize,cex.main=p
ointsize)
legend(maxx+0.08*maxx,maxy,c("Male","Female","X","Y","Z"),lty=1,lwd=linethickness,col
=c("black","black","blue","green","red"))
```

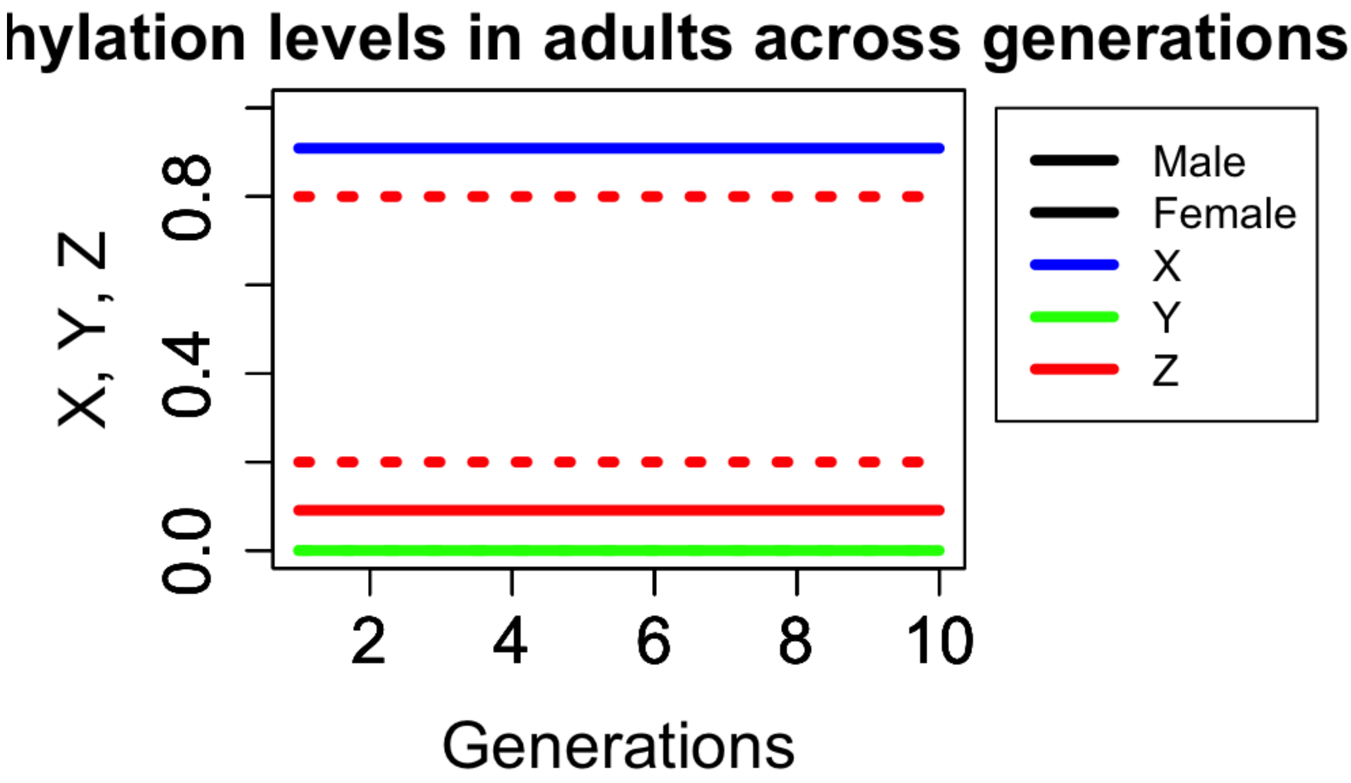

Hide

```
if(saveplots=="T"|saveplots=="TRUE"){
 dev.off()
}
```

Plot 9

This plot shows the methylation dynamics during the first two generations.

Hide

```
if(saveplots=="T"|saveplots=="TRUE"){
 pdf(file=paste0("article1-output/",fileout,"plot9.pdf"),width=30,height=20,pointsize
=30)
}
pointsize<-1.5 #Size of text.
linethickness<-4 #Line thickness.
minx<-0 #Minimum of x-axis.
maxx<-2*ndiv #Maximum of x-axis.
miny<-0 #Minimum of y-axis.
maxy<-1.0 #Maximum of x-axis.
par(xpd=T,mar=par()$mar+c(0,1,0,6))
plot(c(as.vector(t(Xm[1:2,]))),type="l",lty=1,lwd=linethickness,col="blue",cex.axis=p
ointsize,cex.lab=pointsize,cex.main=pointsize,main="Methylation dynamics during gener
ations #1 and #2",xlab="Cell division",ylab="X, Y, Z",xlim=c(minx,maxx), ylim=c(miny,
maxy))
par(new=T)
```

Hide

```
plot(c(as.vector(t(Zm[1:2,]))),type="l",lty=1,lwd=linethickness,col="red",cex.axis=po
intsize,cex.lab=pointsize,cex.main=pointsize,main=NA,xlab=NA,ylab=NA,xlim=c(minx,maxx
), ylim=c(miny,maxy))
par(new=T)
```

Hide

```
plot(c(as.vector(t(Ym[1:2,]))),type="l",lty=1,lwd=linethickness,col="green",cex.axis=
pointsize,cex.lab=pointsize,cex.main=pointsize,main=NA,xlab=NA,ylab=NA,xlim=c(minx,ma
xx), ylim=c(miny,maxy))
par(new=T)
```

Hide

```
plot(c(as.vector(t(Xf[1:2,]))),type="l",lty=3,lwd=linethickness,col="blue",cex.axis=p
ointsize,cex.lab=pointsize,cex.main=pointsize,main=NA,xlab=NA,ylab=NA,xlim=c(minx,max
x), ylim=c(miny,maxy))
par(new=T)
```

Hide

```
plot(c(as.vector(t(Zf[1:2,]))),type="l",lty=3,lwd=linethickness,col="red",cex.axis=po
intsize,cex.lab=pointsize,cex.main=pointsize,main=NA,xlab=NA,ylab=NA,xlim=c(minx,maxx
), ylim=c(miny,maxy))
par(new=T)
```

Hide

```
plot(c(as.vector(t(Yf[1:2,]))),type="l",lty=3,lwd=linethickness,col="green",cex.axis=
pointsize,cex.lab=pointsize,cex.main=pointsize,main=NA,xlab=NA,ylab=NA,xlim=c(minx,ma
xx), ylim=c(miny,maxy))
polygon(x=c(ndiv+1+eqdivXm2,ndiv+1+eqdivXm2),y=c(eqXm2-0.03,eqXm2+0.03),lty=1,lwd=lin
ethickness,border="purple")
```

Hide

```
polygon(x=c(ndiv+1+eqdivXf2,ndiv+1+eqdivXf2),y=c(eqXf2-0.03,eqXf2+0.03),lty=1,lwd=lin
ethickness,border="purple")
legend(maxx+0.08*maxx,maxy,c("Male","Female","X","Y","Z","Xeq"),lty=c(1,3,1,1,1,1),lw
d=linethickness,col=c("black","black","blue","green","red","purple"))
```

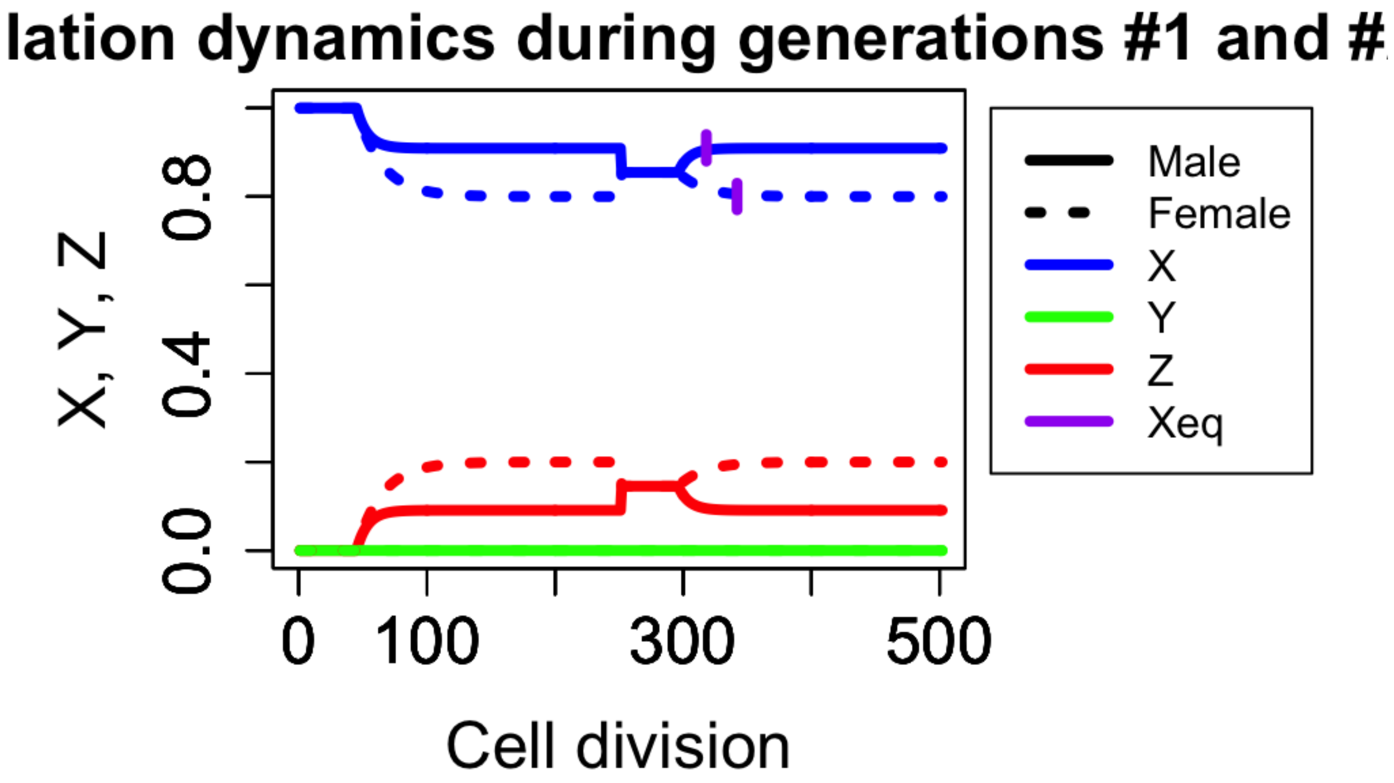

Hide

```
if(saveplots=="T"|saveplots=="TRUE"){
 dev.off()
}
```

Parameter exploration

Set parameter values

Hide

```
ndiv<-250 #Number of cell divisions per generation.
r<-0.1 #Intrinsic cell division rate.
K<-1024 #Number of cells at developmental equilibrium.
rho0<-1.0 #Initial amount of maternal repressor per generation (as a proportion of
 r).
rhod<-0.1 #Maternal repressor degradation rate.
nuthreshold<-1/100 #Nucleocytoplasmic ration at which the ZGA takes place.
X0<-1 #Initial proportion of homomethylated sites.
Y0<-0 #Initial proportion of hemimethylated sites.
Z0<-0 #Initial proportion of unmethylated sites.
nalpha<-100
nbeta<-100
ndelta<-100
nzeta<-100
accuracy<-2 #Accuracy of values (number of decimals).
kroneckermodel<-"F" #TRUE for kronecker (boolean) model and FALSE for Heaviside mode
l.
heavisideslope<-100 #Steepness of heaviside step function (high values for abrupt cha
nge) when kroneckermodel=FALSE.
note<-"Parameter estimation." #Scenario description.
#Original values: simmet(seed=001, ngeninput=ngen, ndivinput=ndiv, rinput=0.1, Kinput
=1024, rho0input=1.0, rhodinput=0.2, nuthresholdinput=1.0, Xm0=1.0, Ym0=0.0, Zm0=0.0,
 Xf0=1.0, Yf0=0.0, Zf0=0.0, alphamale=alpha, betamale=beta, deltamale=delta, zetamale
=zeta, alphafemale=alpha, betafemale=beta, deltafemale=delta, zetafemale=zeta, alphae
nvmin=-0.01, alphaenvmax=0.01, deltaenvmin=-0.01, deltaenvmax=0.01, distmale="F", dis
tfemale="F", accuracyinput=2, devgeninput=1, devdistinput="F", devfreqinput=25, gendi
stinput="F", genfreqinput=25, plots="F")
```

Create matrices

Hide

```
if(parexplor=="T"|parexplor=="TRUE"){
  #Matrix of final methylation levels for each combination of alpha, beta, delta and
 zeta.
  #parresultsXs<-array(data=100,dim=c(nalpha+1,nbeta+1,ndelta+1,nzeta+1))
  #parresultsZs<-array(data=100,dim=c(nalpha+1,nbeta+1,ndelta+1,nzeta+1))
  #parresultsYs<-array(data=100,dim=c(nalpha+1,nbeta+1,ndelta+1,nzeta+1))

  #Simple matrix of final methylation levels for each combination of alpha, beta, del
ta and zeta.
  #Column1=combindex,column2=alpha,column3=beta,column4=delta,column5=zeta,column6=pa
rXs[ndiv+1],column7=parYs[ndiv+1],column8=parZs[ndiv+1].
  ncomb<-(nalpha+1)*(nbeta+1)*(ndelta+1)*(nzeta+1) #Number of combinations.
  parresults<-array(data=100,dim=c(ncomb,9))
  colnames(parresults)<-c("n","alpha","beta","delta","zeta","X","Y","Z","eqdiv")

  #Store values of X, Y and Z across cell divisions.
  parXs<-array(data=0,dim=ndiv+1) ; parXs[1]<-X0
  parYs<-array(data=0,dim=ndiv+1) ; parYs[1]<-Y0
  parZs<-array(data=0,dim=ndiv+1) ; parZs[1]<-Z0

  #Values of cell division dynamics.
  parncells<-array(data=0,dim=ndiv+1) ; parncells[1]<-1
  parrho<-array(data=0,dim=ndiv+1) ; parrho[1]<-rho0
  parmu<-array(data=0,dim=ndiv+1)
  parnu<-array(data=0,dim=ndiv+1)
}
```

Calculate final methylation values

Hide

```
if(parexplor=="T"|parexplor=="TRUE"){
  starttime<-format(Sys.time(), "%Y%m%d%H%M%S")

  if(kroneckermodel=="T"|kroneckermodel=="TRUE"){

    n<-0 #For counting the number of combination.
    for (a in 0:nalpha){
      for (b in 0:nbeta){
        for(d in 0:ndelta){
            for (z in 0:nzeta){
              alpha<-a/nalpha
            beta<-b/nbeta
            delta<-d/ndelta
            zeta<-z/nzeta
            n<-n+1

            if(n==1|n/1000000==round(n/1000000)){
              print(paste0("Combination #",n, " (alpha=",alpha,", beta=",beta,", delt
a=",delta,", zeta=",zeta,")."))
            }

            if (alpha+delta<=1){
              for (c in 1:ndiv){
                #Cell division dynamics.
                parmu[c]<-(1+parrho[c])*r #Rate of division.
                parncells[c+1]<-parncells[c]+parmu[c]*parncells[c]*(1-parncells[c]/K)
 #Number of cells.
                parnu[c]<-1/parncells[c] #Nucleocytoplasmic ratio.
                parrho[c+1]<-parrho[c]-rhod*parrho[c] #Amount of maternal repressor.

                #Calculate rate of division and nucleocytoplasmic ratio of last devel
opmental division.
                if (c==ndiv){
                  parmu[c+1]<-(1+parrho[c+1])*r
                  parnu[c+1]<-1/parncells[c+1]
                }

                #Calculate the impact of rho (rhoc) on methylation dynamics when ZGA
 is triggered.
                if (rho0>0){
                  rhoc<-(rho0-parrho[c])/rho0
                }else{
                  rhoc<-1
                }
                if (parnu[c]>nuthreshold){
                  parXs[c+1]<-parXs[c]; parYs[c+1]<-parYs[c]; parZs[c+1]<-parZs[c]
                }else{
                  #Methylation levels after DNA duplication.
                  Xsd<-0/2
                  Ysd<-(2*parXs[c]+parYs[c])/2
                  Zsd<-(2*parZs[c]+parYs[c])/2

                  #Methylation levels after methylation repair and delay due to mater
nal effect.
                  Xsr<-alpha*Ysd+beta*Zsd
                  Ysr<-(1-alpha-rhoc*delta)*Ysd
                  Zsr<-(1-beta)*Zsd+rhoc*delta*Ysd
```

```
                #Methylation levels after active demethylation.
                Xsm<-(1-zeta)*Xsr
                Ysm<-Ysr
                Zsm<-Zsr+zeta*Xsr

                parXs[c+1]<-Xsm
                parYs[c+1]<-Ysm
                parZs[c+1]<-Zsm
              }
            }
            parresults[n,1]<-n
            parresults[n,2]<-alpha
            parresults[n,3]<-beta
            parresults[n,4]<-delta
            parresults[n,5]<-zeta
            parresults[n,6]<-parXs[ndiv+1]
            parresults[n,7]<-parYs[ndiv+1]
            parresults[n,8]<-parZs[ndiv+1]
            parresults[n,9]<-sum(round(parXs,digits=2)!=round(parXs[ndiv+1],digits=
2))

            #parresultsXs[a+1,b+1,d+1,z+1]<-parXs[ndiv+1]
            #parresultsZs[a+1,b+1,d+1,z+1]<-parZs[ndiv+1]
            #parresultsYs[a+1,b+1,d+1,z+1]<-parYs[ndiv+1]
          }else{
            parresults[n,1]<-n
            parresults[n,2]<-alpha
            parresults[n,3]<-beta
            parresults[n,4]<-delta
            parresults[n,5]<-zeta
            parresults[n,6]<-NA
            parresults[n,7]<-NA
            parresults[n,8]<-NA
            parresults[n,9]<-NA
          }
          }
      }
    }
  }
}

if(kroneckermodel=="F"|kroneckermodel=="FALSE"){

  #Heaviside step Functions.
  heaviside01<-function(steepness,nuthreshold,div,gofrom,goto){
    ((goto-gofrom)/(1+exp(-steepness*(div-nuthreshold))))+gofrom
  } #This functions steps up (0 to 1) or down (1 to 0).

  n<-0 #For counting the number of combination.
  for (a in 0:nalpha){
    for (b in 0:nbeta){
      for(d in 0:ndelta){
          for (z in 0:nzeta){
            alpha<-a/nalpha
          beta<-b/nbeta
          delta<-d/ndelta
          zeta<-z/nzeta
```

```
            n<-n+1

            if(n==1|n/1000000==round(n/1000000)){
              print(paste0("Combination #",n, " (alpha=",alpha,", beta=",beta,", delt
a=",delta,", zeta=",zeta,")."))
            }

            if (alpha+delta<=1){
              for (c in 1:ndiv){
                #Cell division dynamics.
                parmu[c]<-(1+parrho[c])*r #Rate of division.
                parncells[c+1]<-parncells[c]+parmu[c]*parncells[c]*(1-parncells[c]/K)
 #Number of cells.
                parnu[c]<-1/parncells[c] #Nucleocytoplasmic ratio.
                parrho[c+1]<-parrho[c]-rhod*parrho[c] #Amount of maternal repressor.

                #Calculate rate of division and nucleocytoplasmic ratio of last devel
opmental division.
                if (c==ndiv){
                  parmu[c+1]<-(1+parrho[c+1])*r
                  parnu[c+1]<-1/parncells[c+1]
                }

                #Calculate the impact of rho (rhoc) on methylation dynamics when ZGA
 is triggered.
                if (rho0>0){
                  rhoc<-(rho0-parrho[c])/rho0
                }else{
                  rhoc<-1
                }
                halpha<-heaviside01(heavisideslope,(nuthreshold)^(-1),parncells[c],1.
0,alpha)
                hbeta<-heaviside01(heavisideslope,(nuthreshold)^(-1),parncells[c],0.0
,beta)
                hdelta<-heaviside01(heavisideslope,(nuthreshold)^(-1),parncells[c],0.
0,delta)
                hzeta<-heaviside01(heavisideslope,(nuthreshold)^(-1),parncells[c],0.0
,zeta)

                #Methylation levels after DNA duplication.
                Xsd<-0/2
                Ysd<-(2*parXs[c]+parYs[c])/2
                Zsd<-(2*parZs[c]+parYs[c])/2

                #Methylation levels after methylation repair and delay due to materna
l effect.
                Xsr<-halpha*Ysd+hbeta*Zsd
                Ysr<-(1-halpha-rhoc*hdelta)*Ysd
                Zsr<-(1-hbeta)*Zsd+rhoc*hdelta*Ysd

                #Methylation levels after active demethylation.
                Xsm<-(1-hzeta)*Xsr
                Ysm<-Ysr
                Zsm<-Zsr+hzeta*Xsr

                parXs[c+1]<-Xsm
                parYs[c+1]<-Ysm
                parZs[c+1]<-Zsm
```

```
              }
              parresults[n,1]<-n
              parresults[n,2]<-alpha
              parresults[n,3]<-beta
              parresults[n,4]<-delta
              parresults[n,5]<-zeta
              parresults[n,6]<-parXs[ndiv+1]
              parresults[n,7]<-parYs[ndiv+1]
              parresults[n,8]<-parZs[ndiv+1]
              parresults[n,9]<-sum(round(parXs,digits=2)!=round(parXs[ndiv+1],digits=
2))

              #parresultsXs[a+1,b+1,d+1,z+1]<-parXs[ndiv+1]
              #parresultsZs[a+1,b+1,d+1,z+1]<-parZs[ndiv+1]
              #parresultsYs[a+1,b+1,d+1,z+1]<-parYs[ndiv+1]
            }else{
              parresults[n,1]<-n
              parresults[n,2]<-alpha
              parresults[n,3]<-beta
              parresults[n,4]<-delta
              parresults[n,5]<-zeta
              parresults[n,6]<-NA
              parresults[n,7]<-NA
              parresults[n,8]<-NA
              parresults[n,9]<-NA
            }
            }
        }
      }
    }
  }

  endtime<-format(Sys.time(), "%Y%m%d%H%M%S")
}
```

Save files

Hide

```
if(parexplor=="T"|parexplor=="TRUE"){
  #Save input values.
  parinputvalues<-array(data=0,dim=c(2,19))
  parinputvalues[1,1:19]<-c("starttime","endtime","ndiv","r","K","rho0","rhod","nuthr
eshold","X0","Y0","Z0","nalpha","nbeta","ndelta","nzeta","accuracy","kroneckermodel",
"heavisideslope","note")
  parinputvalues[2,1:19]<-c(starttime,endtime,ndiv,r,K,rho0,rhod,nuthreshold,X0,Y0,Z0
,nalpha,nbeta,ndelta,nzeta,accuracy,kroneckermodel,heavisideslope,note)

  #Save results.
  save(parinputvalues,file=paste0("article1-output/parinputvalues-",starttime,".RDat
a"))
  save(parresults,file=paste0("article1-output/parresults-",starttime,".RData"))
  #save(parresultsXs,file=paste0("article1-output/parresultsXs-",starttime,".RData"))
  #save(parresultsYs,file=paste0("article1-output/parresultsYs-",starttime,".RData"))
  #save(parresultsZs,file=paste0("article1-output/parresultsZs-",starttime,".RData"))
}
```

Plots

Load parameter exploration files.

Hide

```
if(parexplor=="F"|parexplor=="FALSE"){
  #load("resultsXm.RData")
  #load("resultsYm.RData")
  #load("resultsZm.RData")

  load("parinputvalues-20170510102541.RData")
  parinputkronecker<-as.data.frame(parinputvalues)
  load("parresults-20170510102541.RData")
  parresultskronecker<-as.data.frame(parresults)

  load("parinputvalues-20170510124942.RData")
  parinputheaviside<-as.data.frame(parinputvalues)
  load("parresults-20170510124942.RData")
  parresultsheaviside<-as.data.frame(parresults)

  load("parinputvalues-20170510124942.RData")
  parinputheaviside2<-as.data.frame(parinputvalues)
  load("parresults-20170510124942.RData")
  parresultsheaviside2<-as.data.frame(parresults)

  load("parinputvalues-20170511095346.RData")
  parinputheavisidex0<-as.data.frame(parinputvalues)
  load("parresults-20170511095346.RData")
  parresultsheavisidex0<-as.data.frame(parresults)
}
if(kroneckermodel=="T"|kroneckermodel=="TRUE"){
  parresultsplots<-parresultskronecker[complete.cases(parresultskronecker),]
}else{
  parresultsplots<-parresultsheavisidex0[complete.cases(parresultsheavisidex0),]
}
```

Plot 10

This plot shows the values of X that can result from a given value of alpha, beta or delta.

Hide

```
if(saveplots=="T"|saveplots=="TRUE"){
 pdf(file=paste0("article1-output/",fileout,"plot10.pdf"),width=30,height=30,pointsiz
e=30)
}
forX0<-1.0
alphainput<-0.90
betainput<-0.20
deltainput<-0.01
alphasubset<-subset(parresultsplots,round(parresultsplots$alpha,digits=2)==alphainput
 & round(parresultsplots$X0,digits=2)==round(forX0,digits=2))
betasubset<-subset(parresultsplots,round(parresultsplots$beta,digits=2)==betainput &
round(parresultsplots$X0,digits=2)==round(forX0,digits=2))
deltasubset<-subset(parresultsplots,round(parresultsplots$delta,digits=2)==deltainput
 & round(parresultsplots$X0,digits=2)==round(forX0),digits=2)
alphaplot<-ggplot(data=alphasubset,aes(x=beta, y=delta, fill=X))+
  ggtitle(paste("X values for alpha=",alphainput))+
  geom_raster(aes(fill=X),interpolate=FALSE)+xlab("Beta")+ylab("Delta")+
  scale_fill_gradient(limits=c(0.0, 1.0),low="white",high="blue")+
  xlim(0.0,1.0)+ylim(0.0,1.0)
betaplot<-ggplot(data=betasubset,aes(x=alpha, y=delta, fill=X))+
  ggtitle(paste("X values for beta=",betainput))+
  geom_raster(aes(fill=X),interpolate=FALSE)+xlab("Alpha")+ylab("Delta")+
  scale_fill_gradient(limits=c(0.0, 1.0),low="white",high="blue")+
  xlim(0.0,1.0)+ylim(0.0,1.0)
deltaplot<-ggplot(data=deltasubset,aes(x=alpha, y=beta, fill=X))+
  ggtitle(paste("X values for delta=",deltainput))+
  geom_raster(aes(fill=X),interpolate=FALSE)+xlab("Alpha")+ylab("Beta")+
  scale_fill_gradient(limits=c(0.0, 1.0),low="white",high="blue")+
  xlim(0.0,1.0)+ylim(0.0,1.0)
grid.arrange(alphaplot,betaplot,deltaplot,ncol=2)
```

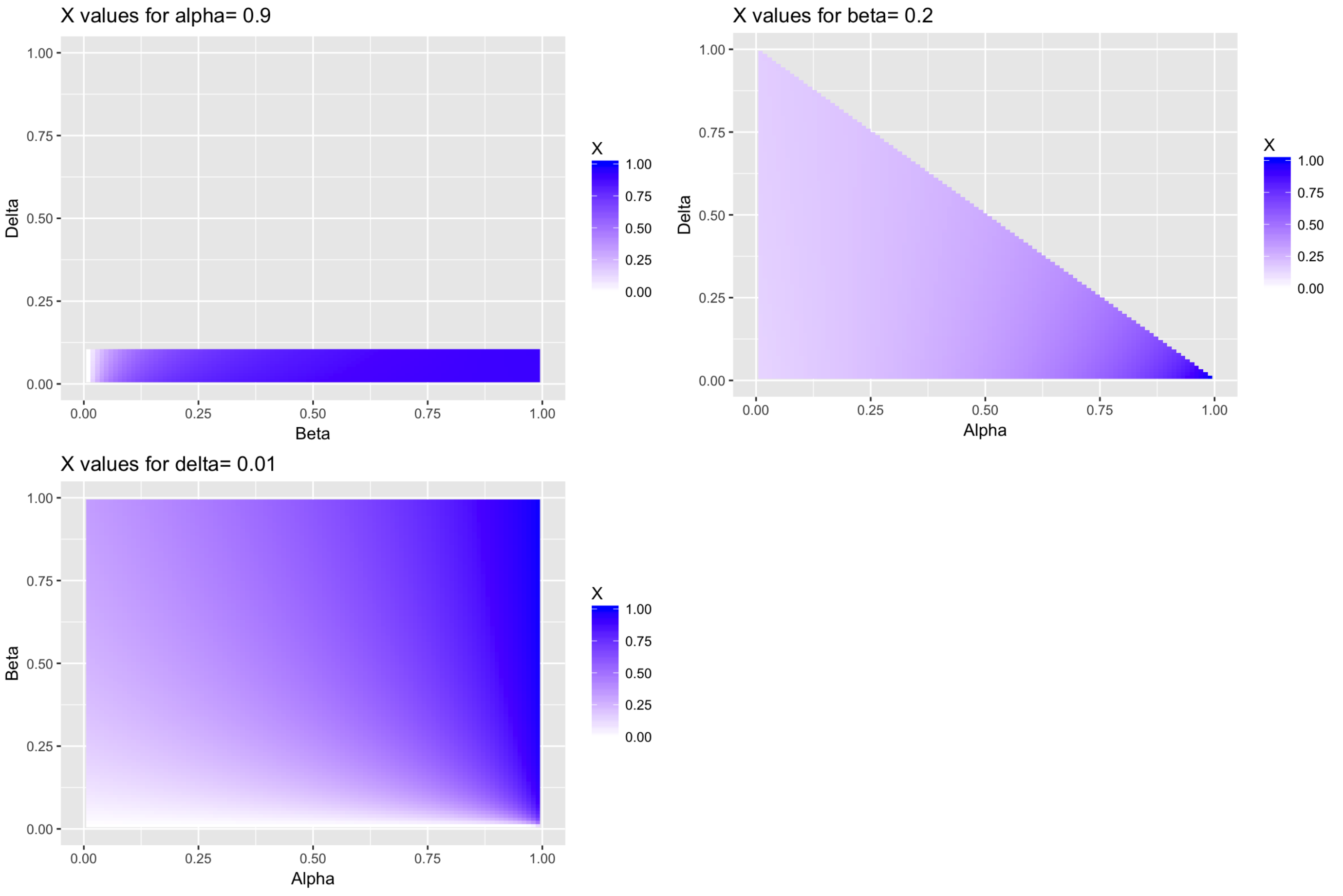

Hide

```
if(saveplots=="T"|saveplots=="TRUE"){
 dev.off()
}
```

Plots 11

These plots show the combinations of values of alpha, beta and delta that yield the specified values of X.

Hide

```
if(saveplots=="T"|saveplots=="TRUE"){
  pdf(file=paste0("article1-output/",fileout,"plot11.pdf"),width=40,height=40,pointsi
ze=30)
}
#Find which combinations of alpha, beta and delta can result in a specific X value.
forX0<-1.0
desiredX=c(0.8,0.91,0.54,0.48,0.3)
#desiredX=0.8 #Zebrafish female.
#desiredX=0.91 #Zebrafish male.
#desiredX=0.54 #Human male.
#desiredX=0.48 #Human female.
#desiredX=0.3 #Hypothetical female.
#desiredX=0.1 #Hypothetical male.
for(i in 1:length(desiredX)){
  Xsubset<-subset(parresultsplots,round(parresultsplots$X,digits=2)==desiredX[i] & ro
und(parresultsplots$X0,digits=2)==round(forX0,digits=2))
  scatterplot3d(Xsubset$alpha,Xsubset$beta,Xsubset$delta,
              highlight.3d=TRUE,col.axis="black",col.grid="lightgrey",
              main=NA,
              xlim=c(0.0,1.0), ylim=c(0.0,1.0), zlim=c(0.0,1.0),
              xlab="Alpha", ylab="Beta", zlab="Delta", pch=20, angle=40)
  par(new=TRUE)
  #scatterplot3d(Xsubset$alpha,Xsubset$beta,Xsubset$delta,
  #             highlight.3d=TRUE,col.axis="black",col.grid="lightgrey",
  #             main=paste0("X=",desiredX[i],", alpha range=",
  #             range(Xsubset$alpha)[1],"-",range(Xsubset$alpha)[2],
  #             ", beta range=",range(Xsubset$beta)[1],"-",range(Xsubset$beta)[2],
  #             ", delta range=", range(Xsubset$delta)[1],"-",range(Xsubset$delta)
[2]),
  #             xlim=c(0.0,1.0), ylim=c(0.0,1.0), zlim=c(0.0,1.0),
  #             xlab="Alpha", ylab="Beta", zlab="Delta", pch=20, angle=40)
}
```

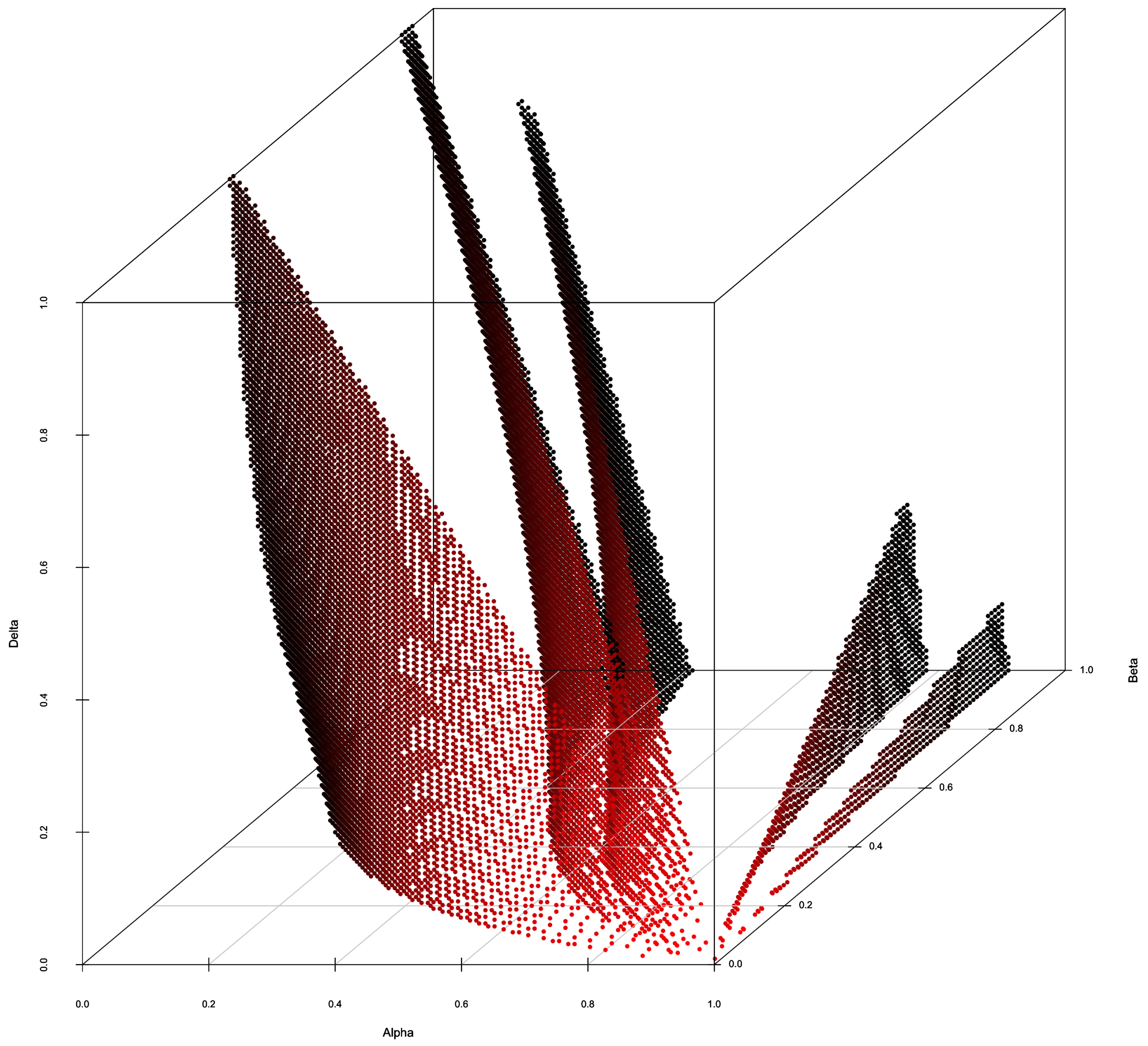

Hide

```
if(saveplots=="T"|saveplots=="TRUE"){
  dev.off()
}
```

Plot 12

This plot shows the ranges of alpha, beta and delta that can result in different values of X.

Hide

```
if(saveplots=="T"|saveplots=="TRUE"){
 pdf(file=paste0("article1-output/",fileout,"plot12.pdf"),width=30,height=30,pointsiz
e=30)
}
forX0<-1.0
par(xpd=T,mar=par()$mar+c(0,0,0,4))
for (i in 0:10){
  desiredX<-i/10
  Xsubset<-subset(parresultsplots,round(parresultsplots$X,digits=2)==desiredX & round
(parresultsplots$X0,digits=2)==round(forX0),digits=2)

  alphaycoord<-rep(desiredX-0.02,length(unique(Xsubset$alpha)))
  plot(unique(Xsubset$alpha),alphaycoord,type="p",pch=16,col="blue",xlim=c(0.0,1.0),y
lim=c(0.0,1.0),xlab="Ranges of alpha, beta and delta",ylab="Final X")
  par(new=TRUE)
  betaycoord<-rep(desiredX,length(unique(Xsubset$beta)))
  plot(unique(Xsubset$beta),betaycoord,type="p",pch=16,col="red",xlim=c(0.0,1.0),ylim
=c(0.0,1.0),xlab=NA,ylab=NA)
  par(new=TRUE)
  deltaycoord<-rep(desiredX+0.02,length(unique(Xsubset$delta)))
  plot(unique(Xsubset$delta),deltaycoord,type="p",pch=16,col="cadetblue",xlim=c(0.0,
1.0),ylim=c(0.0,1.0),xlab=NA,ylab=NA)
  par(new=TRUE)
}
legend(1.08,1.0,c("Alpha","Beta","Delta"),pch=c(16,16,16),col=c("blue","red","cadetbl
ue"))
```

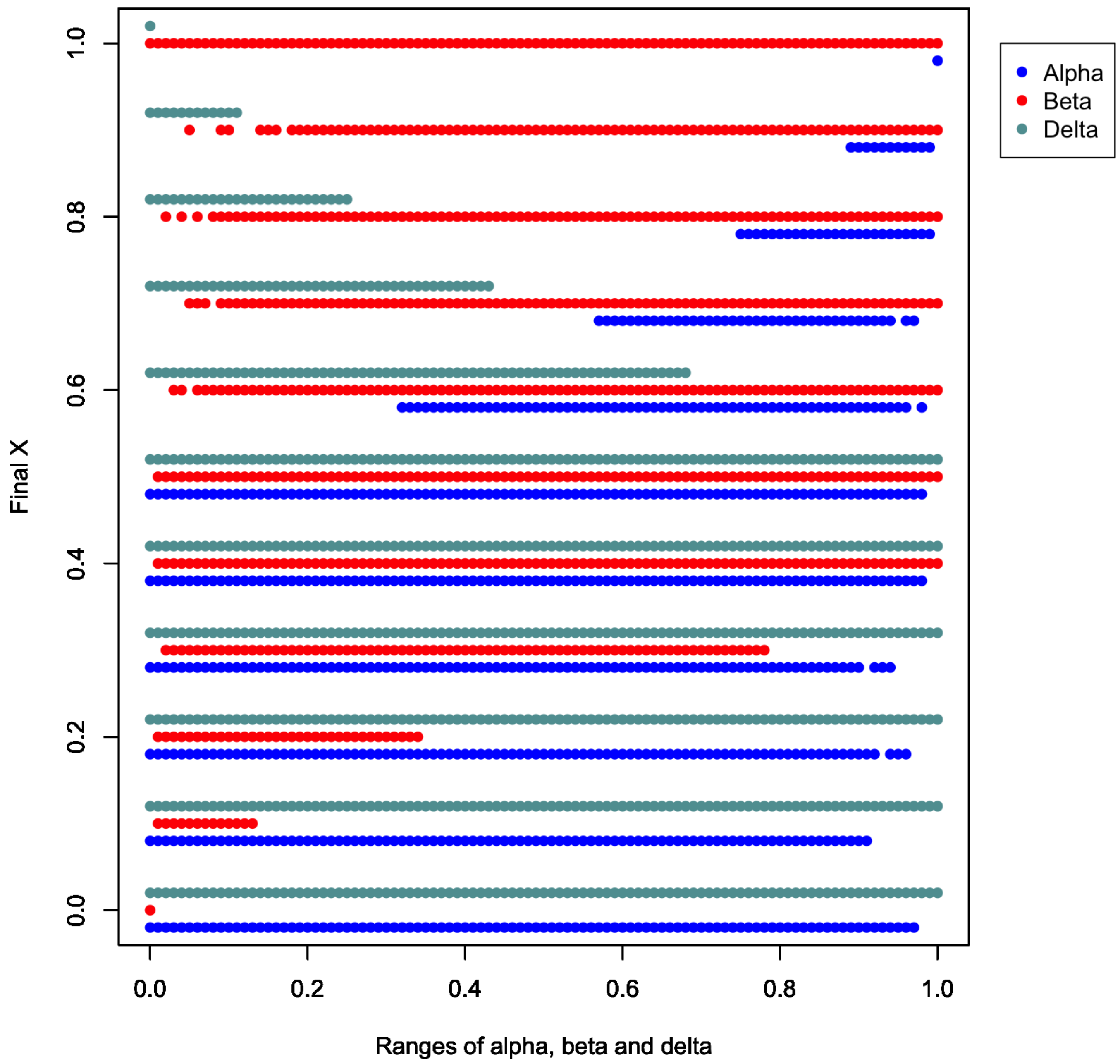

Hide

```
if(saveplots=="T"|saveplots=="TRUE"){
 dev.off()
}
```

Plot 13

This plot shows the number of cell divisions after ZGA that are necessary to reach a specific X for different values of alpha, beta and delta, starting from a specific X0.

Hide

```
if(saveplots=="T"|saveplots=="TRUE"){
 pdf(file=paste0("article1-output/",fileout,"plot13.pdf"),width=40,height=40,pointsiz
e=30)
}
ylow<-0
yhigh<-70
par(xpd=T,mar=par()$mar+c(0,0,0,4))
ZGA<-45 #Number of cell divisions to ZGA. Do not change unless you run parexploration
 again.
desiredX0<-0.85 #Zebrafish zygote.
desiredX<-0.91 #Zebrafish sperm.
X0subset<-subset(parresultsplots,round(parresultsplots$X0,digits=2)==desiredX0 & roun
d(parresultsplots$X,digits=2)==desiredX)
X0alphaplot<-plot(X0subset$alpha,X0subset$eqdiv-ZGA,type="p",pch=16,col="blue",xlim=c
(0.0,1.0),ylim=c(ylow,yhigh),xlab="Alpha, Beta and Delta",ylab="Cell divisions to equ
ilibrium",main=paste0("X0=",desiredX0,", X=",desiredX))
par(new=TRUE)
```

Hide

```
X0betaplot<-plot(X0subset$beta,X0subset$eqdiv-ZGA,type="p",pch=16,col="red",xlim=c(0.
0,1.0),ylim=c(ylow,yhigh),xlab=NA,ylab=NA,main=NA)
par(new=TRUE)
```

Hide

```
X0deltaplot<-plot(X0subset$delta,X0subset$eqdiv-ZGA,type="p",pch=16,col="cadetblue",x
lim=c(0.0,1.0),ylim=c(ylow,yhigh),xlab=NA,ylab=NA,main=NA)
legend(1.08,yhigh,c("Alpha","Beta","Delta"),pch=c(16,16,16),col=c("blue","red","cadet
blue"))
```

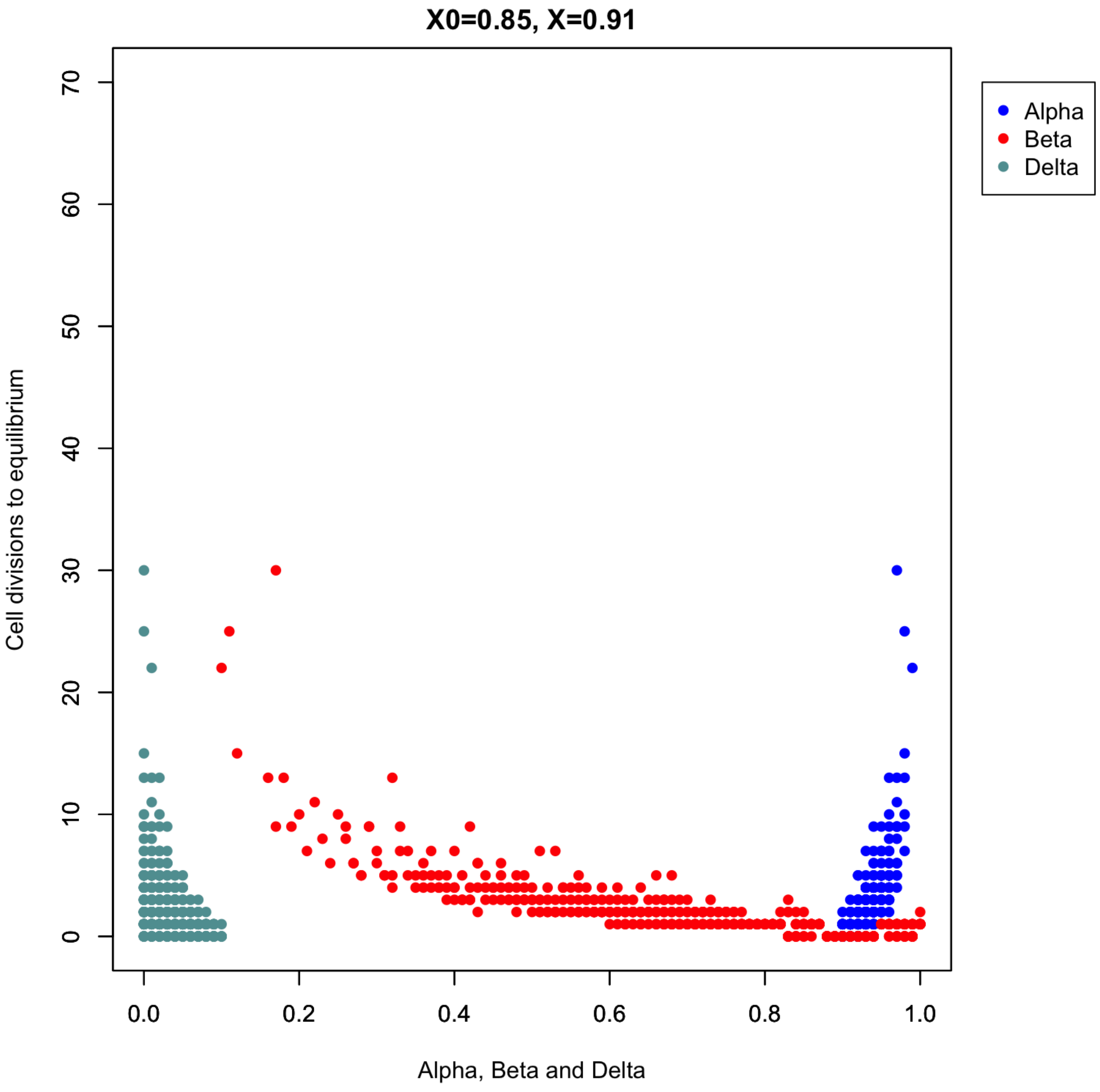

Hide

```
#max(X0subset$eqdiv-ZGA)
desiredX0<-0.85 #Zebrafish zygote.
desiredX<-0.8 #Zebrafish sperm.
X0subset<-subset(parresultsplots,round(parresultsplots$X0,digits=2)==desiredX0 & roun
d(parresultsplots$X,digits=2)==desiredX)
X0alphaplot<-plot(X0subset$alpha,X0subset$eqdiv-ZGA,type="p",pch=16,col="blue",xlim=c
(0.0,1.0),ylim=c(ylow,yhigh),xlab="Alpha, Beta and Delta",ylab="Cell divisions to equ
ilibrium",main=paste0("X0=",desiredX0,", X=",desiredX))
par(new=TRUE)
```

Hide

```
X0betaplot<-plot(X0subset$beta,X0subset$eqdiv-ZGA,type="p",pch=16,col="red",xlim=c(0.
0,1.0),ylim=c(ylow,yhigh),xlab=NA,ylab=NA,main=NA)
par(new=TRUE)
```

Hide

```
X0deltaplot<-plot(X0subset$delta,X0subset$eqdiv-ZGA,type="p",pch=16,col="cadetblue",x
lim=c(0.0,1.0),ylim=c(ylow,yhigh),xlab=NA,ylab=NA,main=NA)
legend(1.08,yhigh,c("Alpha","Beta","Delta"),pch=c(16,16,16),col=c("blue","red","cadet
blue"))
```

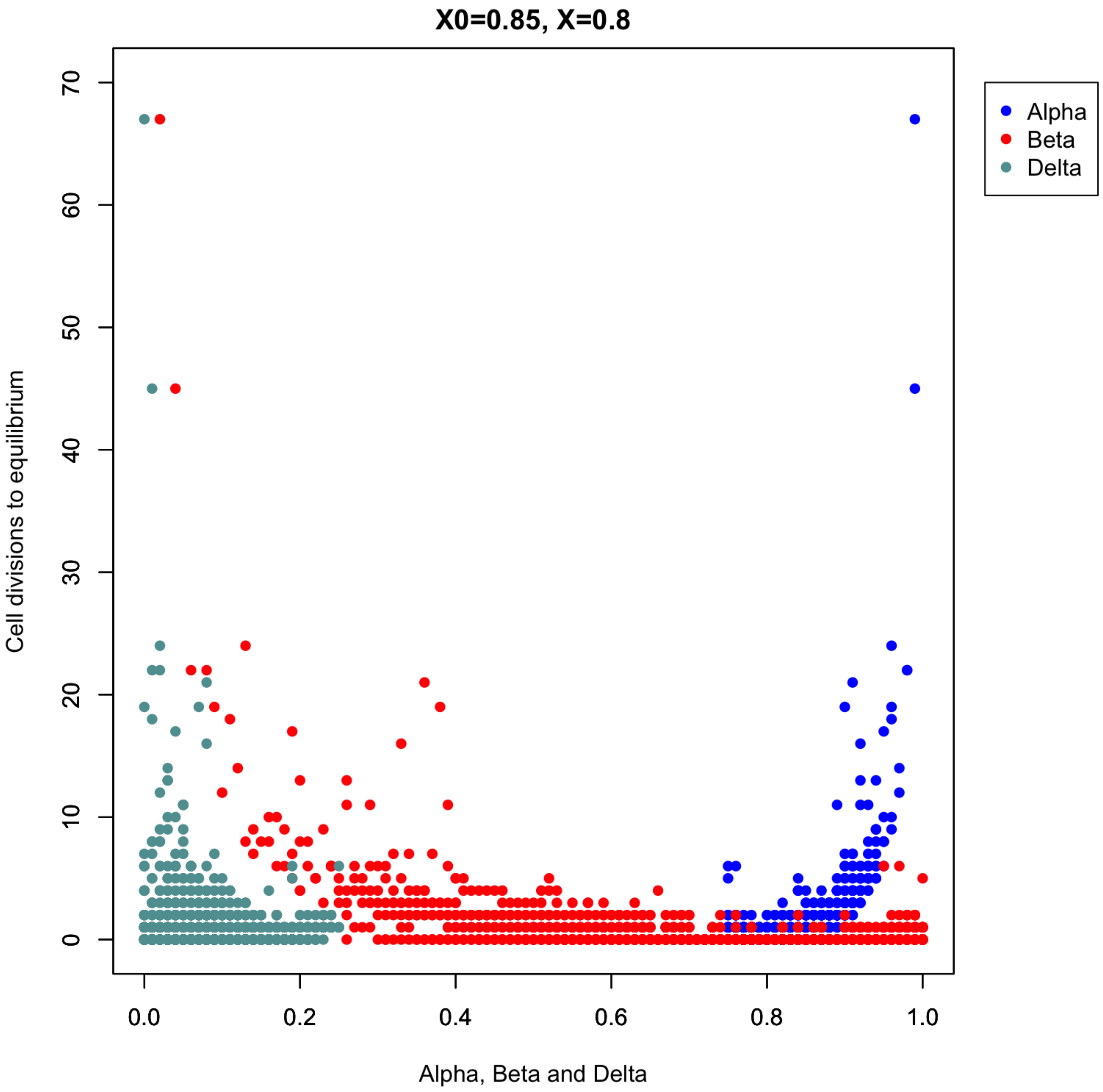

Hide

```
desiredX0<-0.50 #Human zygote.
desiredX<-0.54 #Human sperm.
X0subset<-subset(parresultsplots,round(parresultsplots$X0,digits=2)==desiredX0 & roun
d(parresultsplots$X,digits=2)==desiredX)
X0alphaplot<-plot(X0subset$alpha,X0subset$eqdiv-ZGA,type="p",pch=16,col="blue",xlim=c
(0.0,1.0),ylim=c(ylow,yhigh),xlab="Alpha, Beta and Delta",ylab="Cell divisions to equ
ilibrium",main=paste0("X0=",desiredX0,", X=",desiredX))
par(new=TRUE)
```

Hide

```
X0betaplot<-plot(X0subset$beta,X0subset$eqdiv-ZGA,type="p",pch=16,col="red",xlim=c(0.
0,1.0),ylim=c(ylow,yhigh),xlab=NA,ylab=NA,main=NA)
par(new=TRUE)
```

Hide

```
X0deltaplot<-plot(X0subset$delta,X0subset$eqdiv-ZGA,type="p",pch=16,col="cadetblue",x
lim=c(0.0,1.0),ylim=c(ylow,yhigh),xlab=NA,ylab=NA,main=NA)
legend(1.08,yhigh,c("Alpha","Beta","Delta"),pch=c(16,16,16),col=c("blue","red","cadet
blue"))
```

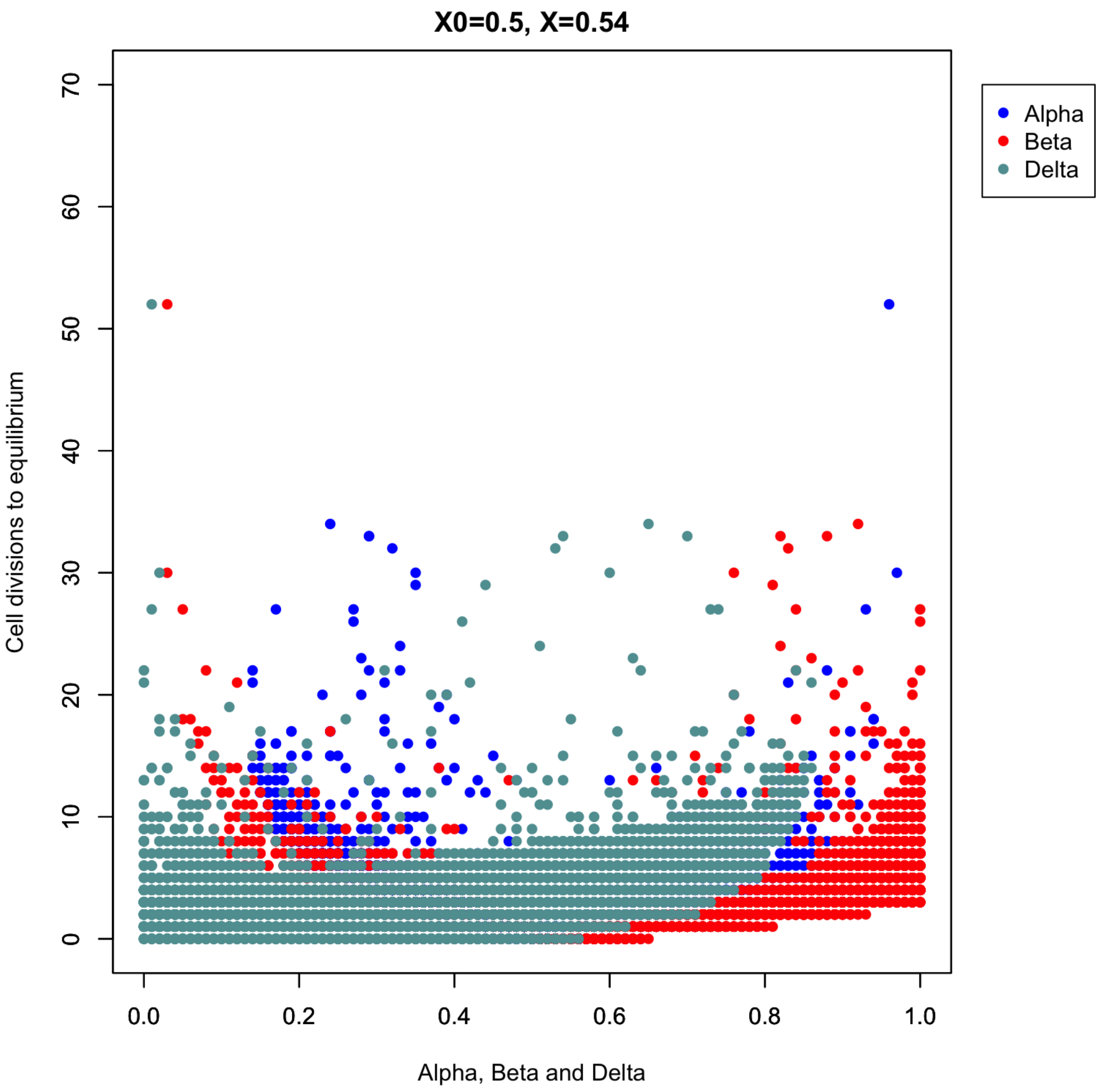

Hide

```
X0alphaplot<-plot(X0subset$alpha,X0subset$eqdiv-ZGA,type="p",pch=16,col="blue",xlim=c
(0.0,1.0),ylim=c(ylow,yhigh),xlab="Alpha, Beta and Delta",ylab="Cell divisions to equ
ilibrium",main=paste0("X0=",desiredX0,", X=",desiredX))
legend(1.08,yhigh,c("Alpha","Beta","Delta"),pch=c(16,16,16),col=c("blue","red","cadet
blue"))
```

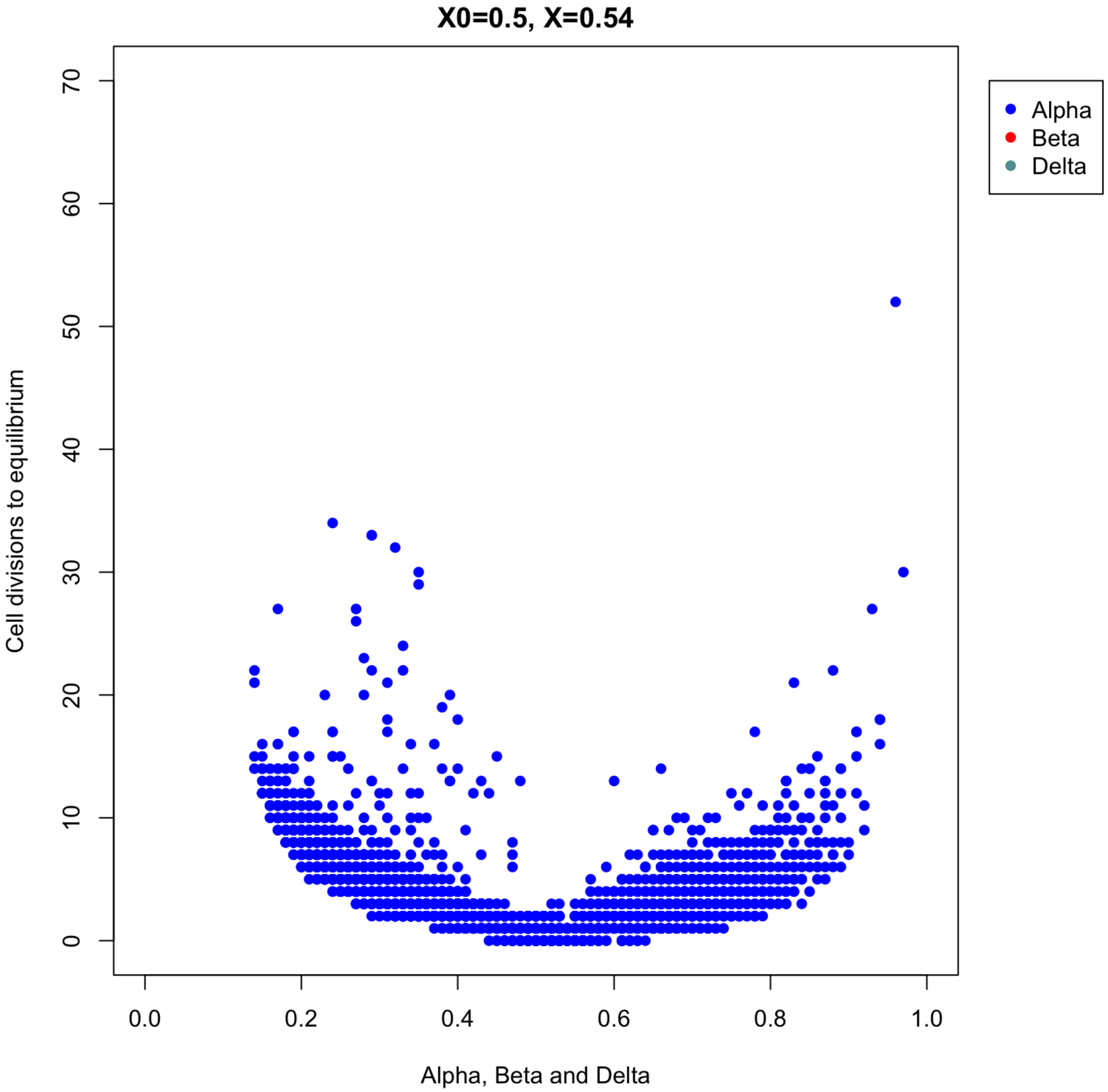

Hide

```
#par(new=TRUE)
X0betaplot<-plot(X0subset$beta,X0subset$eqdiv-ZGA,type="p",pch=16,col="red",xlim=c(0.
0,1.0),ylim=c(ylow,yhigh),xlab="Alpha, Beta and Delta",ylab="Cell divisions to equili
brium",main=paste0("X0=",desiredX0,", X=",desiredX))
legend(1.08,yhigh,c("Alpha","Beta","Delta"),pch=c(16,16,16),col=c("blue","red","cadet
blue"))
```

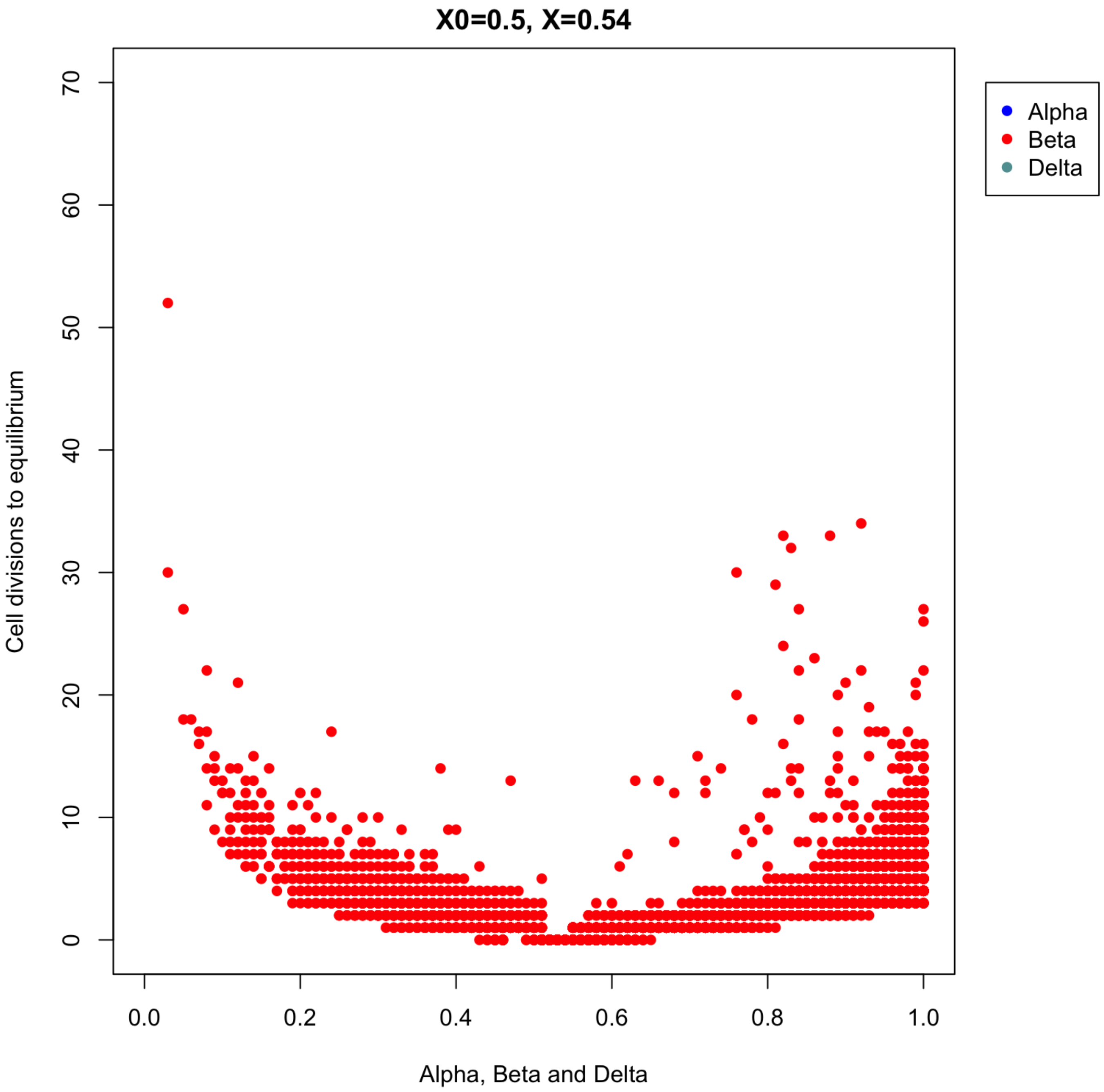

Hide

```
#par(new=TRUE)
X0deltaplot<-plot(X0subset$delta,X0subset$eqdiv-ZGA,type="p",pch=16,col="cadetblue",xlim=c(0.0,1.0),ylim=c(ylow,yhigh),xlab="Alpha, Beta and Delta",ylab="Cell divisions to equilibrium",main=paste0("X0=",desiredX0,", X=",desiredX))
legend(1.08,yhigh,c("Alpha","Beta","Delta"),pch=c(16,16,16),col=c("blue","red","cadetblue"))
```

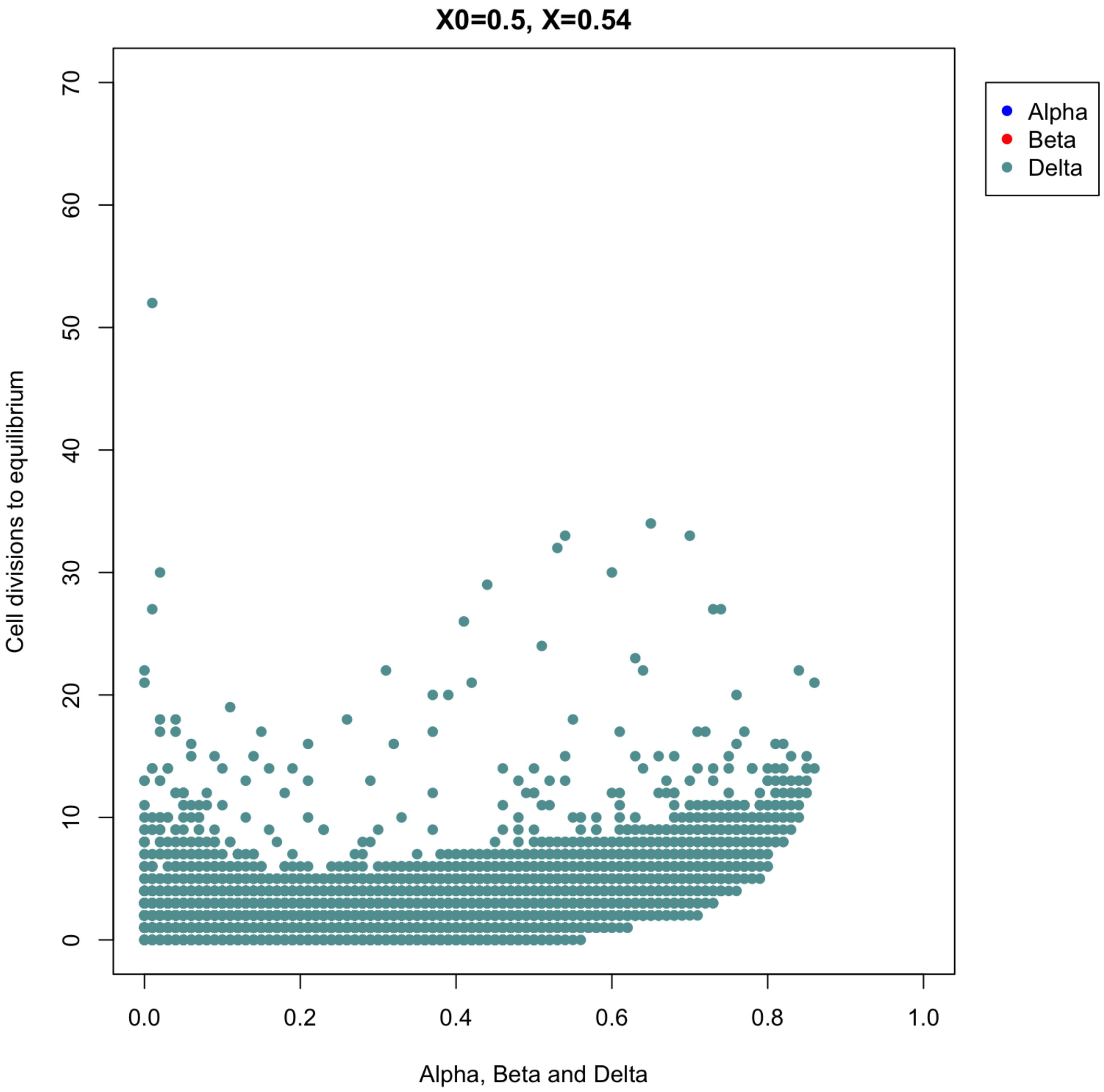

Hide

```
desiredX0<-0.50 #Hypothetical in which the average is 0.50 and the final is 0.90.
desiredX<-0.90 #Hypothetical gamete.
X0subset<-subset(parresultsplots,round(parresultsplots$X0,digits=2)==desiredX0 & roun
d(parresultsplots$X,digits=2)==desiredX)
X0alphaplot<-plot(X0subset$alpha,X0subset$eqdiv-ZGA,type="p",pch=16,col="blue",xlim=c
(0.0,1.0),ylim=c(ylow,yhigh),xlab="Alpha, Beta and Delta",ylab="Cell divisions to equ
ilibrium",main=paste0("X0=",desiredX0,", X=",desiredX))
par(new=TRUE)
```

Hide

```
X0betaplot<-plot(X0subset$beta,X0subset$eqdiv-ZGA,type="p",pch=16,col="red",xlim=c(0.
0,1.0),ylim=c(ylow,yhigh),xlab=NA,ylab=NA,main=NA)
par(new=TRUE)
```

Hide

```
X0deltaplot<-plot(X0subset$delta,X0subset$eqdiv-ZGA,type="p",pch=16,col="cadetblue",x
lim=c(0.0,1.0),ylim=c(ylow,yhigh),xlab=NA,ylab=NA,main=NA)
legend(1.08,yhigh,c("Alpha","Beta","Delta"),pch=c(16,16,16),col=c("blue","red","cadet
blue"))
```

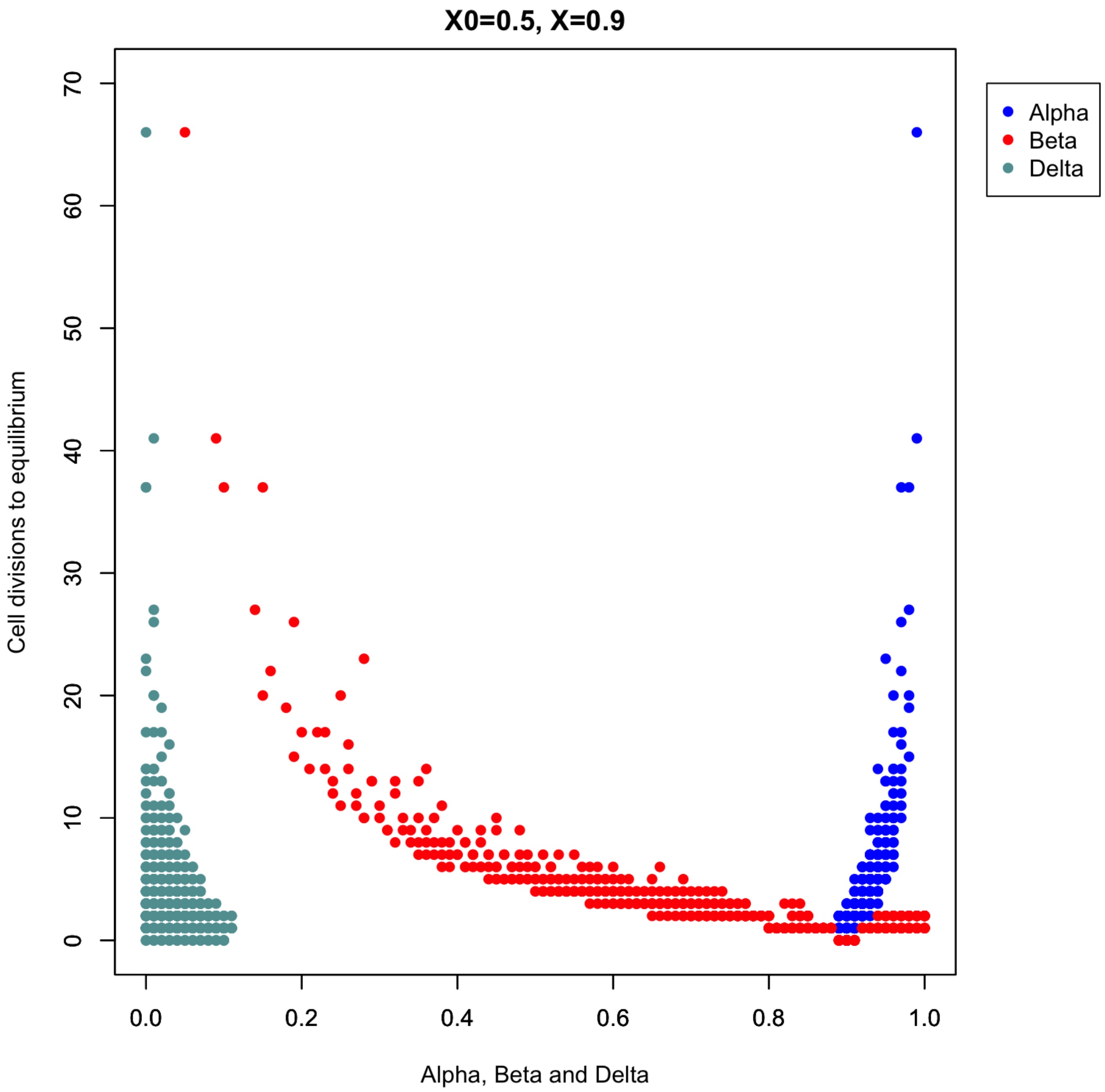

Hide

```
if(saveplots=="T"|saveplots=="TRUE"){
 dev.off()
}
```

Plot 14

This plot shows the number of cell divisions after ZGA that are necessary to reach a specific X, starting from different initial values of X0.

Hide

```
if(saveplots=="T"|saveplots=="TRUE"){
 pdf(file=paste0("article1-output/",fileout,"plot14.pdf"),width=30,height=30,pointsiz
e=30)
}
ZGA<-45 #Number of cell divisions to ZGA. Do not change unless you run parexploration
 again.
desiredX<-0.9
Xsubset<-subset(parresultsplots,round(parresultsplots$X,digits=2)==desiredX)
X0divplot1<-plot(Xsubset$X0,Xsubset$eqdiv-ZGA,type="p",pch=16,col="blue",xlim=c(0.0,
1.0),ylim=c(0,max(Xsubset$eqdiv-ZGA)),xlab="X0",ylab="Cell divisions to equilibrium",
main=paste0("X=",desiredX))
```

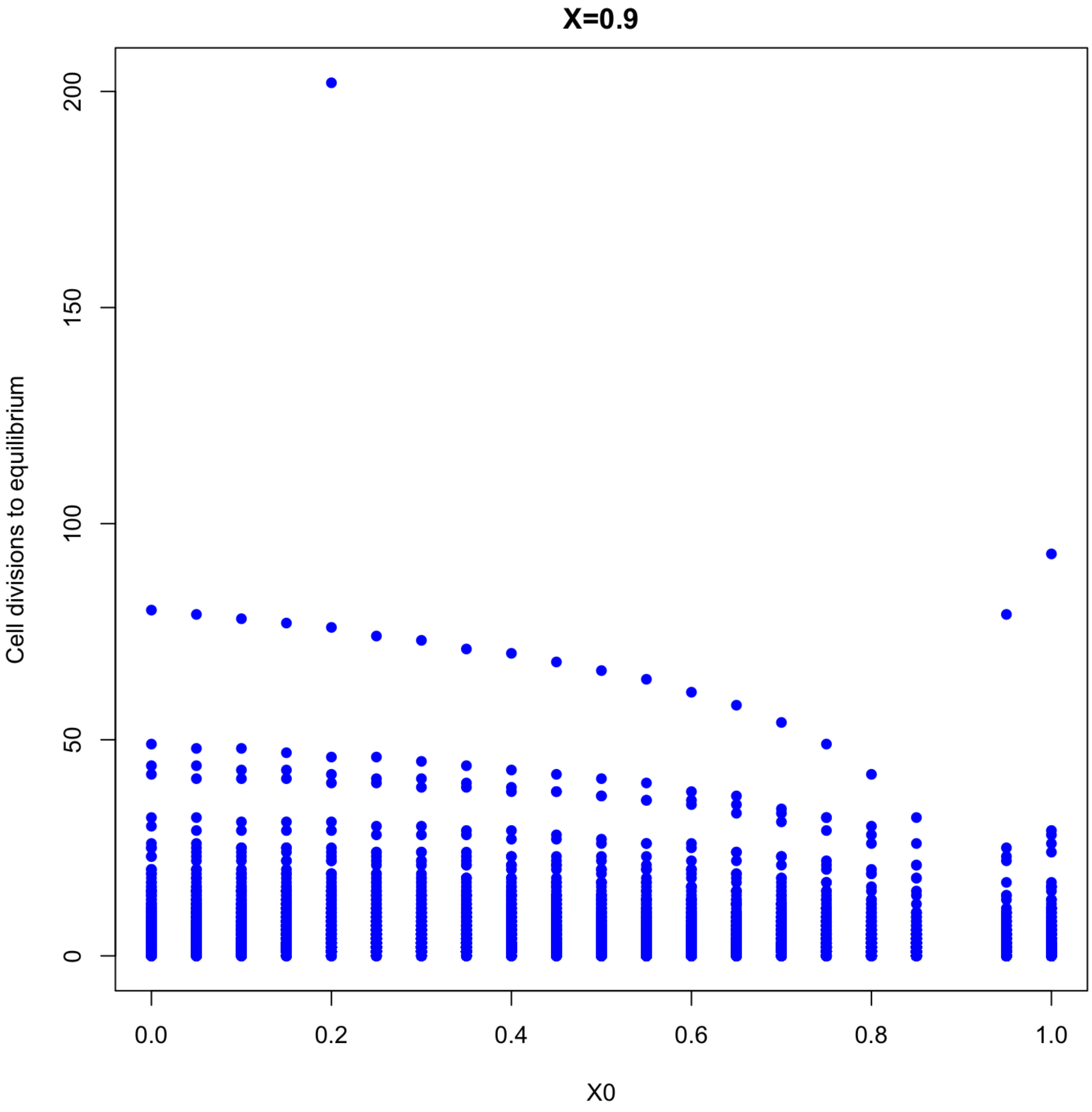

Hide

```
desiredX<-0.5
Xsubset<-subset(parresultsplots,round(parresultsplots$X,digits=2)==desiredX)
X0divplot2<-plot(Xsubset$X0,Xsubset$eqdiv-ZGA,type="p",pch=16,col="blue",xlim=c(0.0,
1.0),ylim=c(0,max(Xsubset$eqdiv-ZGA)),xlab="X0",ylab="Cell divisions to equilibrium",
main=paste0("X=",desiredX))
```

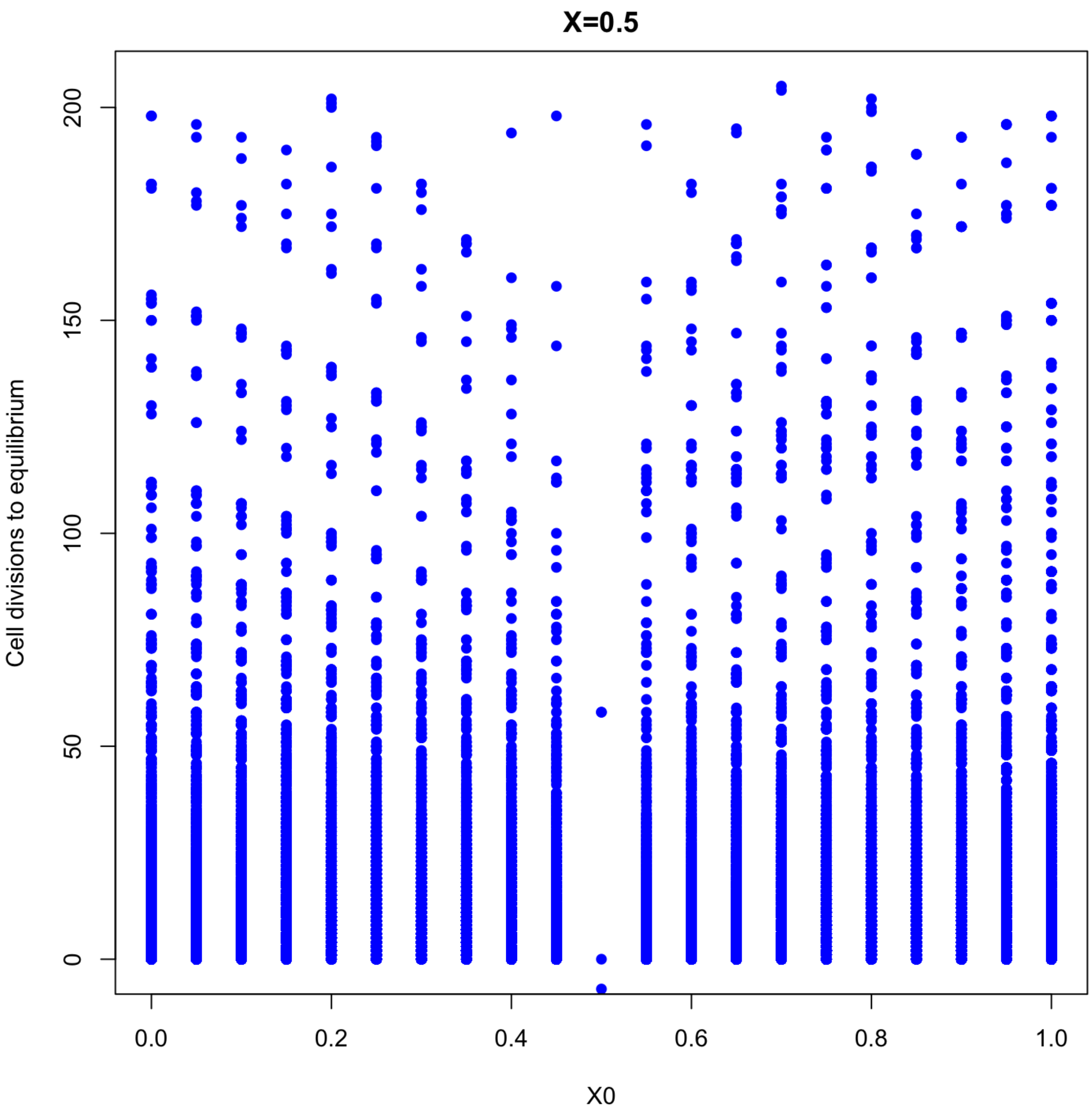

Hide

```
desiredX<-0.1
Xsubset<-subset(parresultsplots,round(parresultsplots$X,digits=2)==desiredX)
X0divplot3<-plot(Xsubset$X0,Xsubset$eqdiv-ZGA,type="p",pch=16,col="blue",xlim=c(0.0,
1.0),ylim=c(0,max(Xsubset$eqdiv-ZGA)),xlab="X0",ylab="Cell divisions to equilibrium",
main=paste0("X=",desiredX))
```

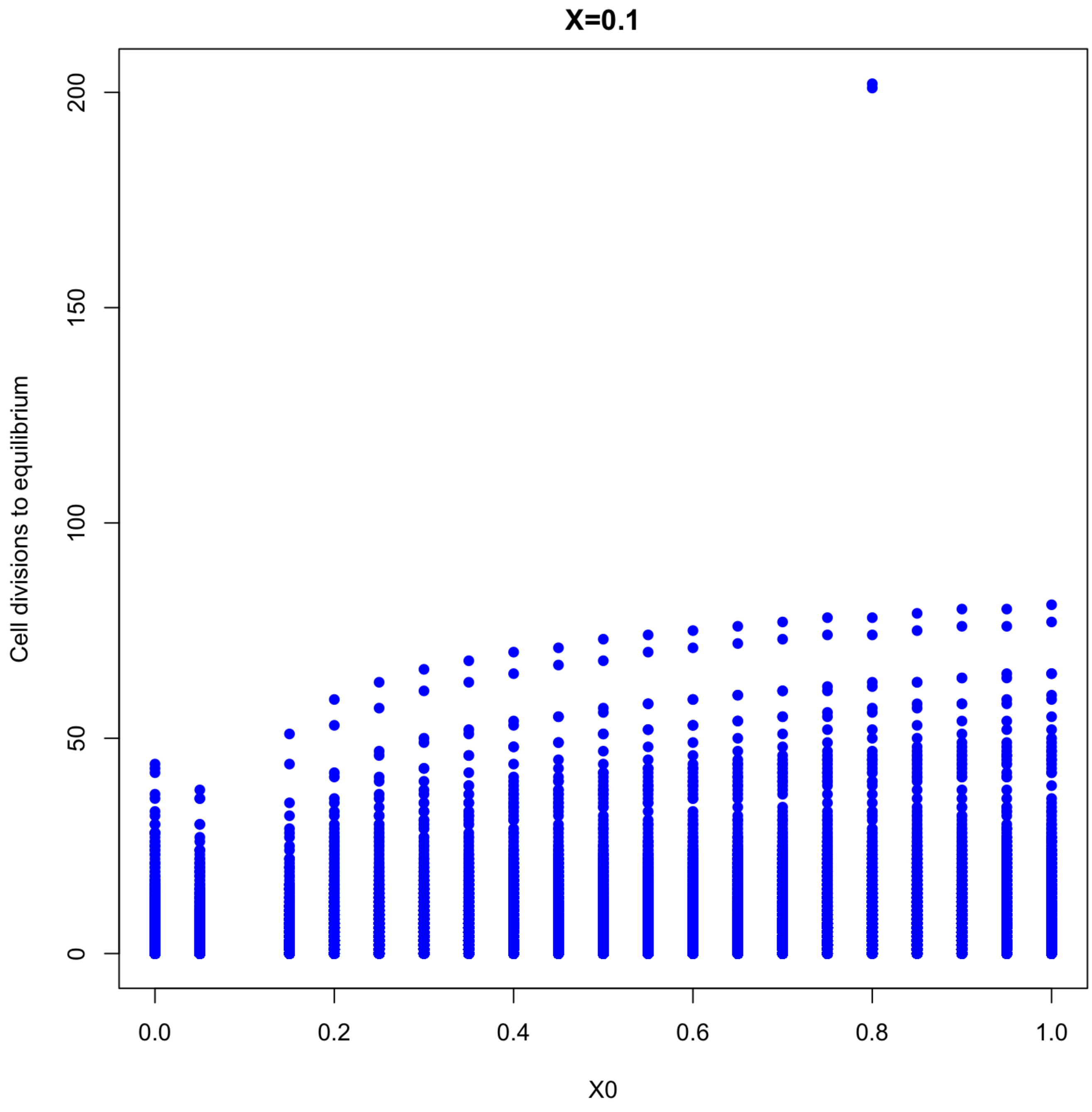

Hide

```
if(saveplots=="T"|saveplots=="TRUE"){
 dev.off()
}
```

Plot 15

This plot shows the number of cell divisions after ZGA that are necessary to reach the equilibrium X, for specific values of alpha, beta and delta, starting from different initial values of X0.

Hide

```
if(saveplots=="T"|saveplots=="TRUE"){
 pdf(file=paste0("article1-output/",fileout,"plot15.pdf"),width=30,height=30,pointsiz
e=30)
}
ZGA<-45 #Number of cell divisions to ZGA. Do not change unless you run parexploration
 again.
desiredalpha<-0.99
desiredbeta<-0.1
desireddelta<-0.01
Xsubset<-subset(parresultsplots,round(parresultsplots$alpha,digits=2)==desiredalpha &
 round(parresultsplots$beta,digits=2)==desiredbeta & round(parresultsplots$delta,digi
ts=2)==desireddelta)
X0divplot<-plot(Xsubset$X0,Xsubset$eqdiv-ZGA,type="p",pch=16,col="blue",xlim=c(0.0,1.
0),ylim=c(0,250),xlab="X0",ylab="Cell divisions to equilibrium",main=paste0("Alpha=",
desiredalpha,", Beta=",desiredbeta,", Delta=",desireddelta))
```

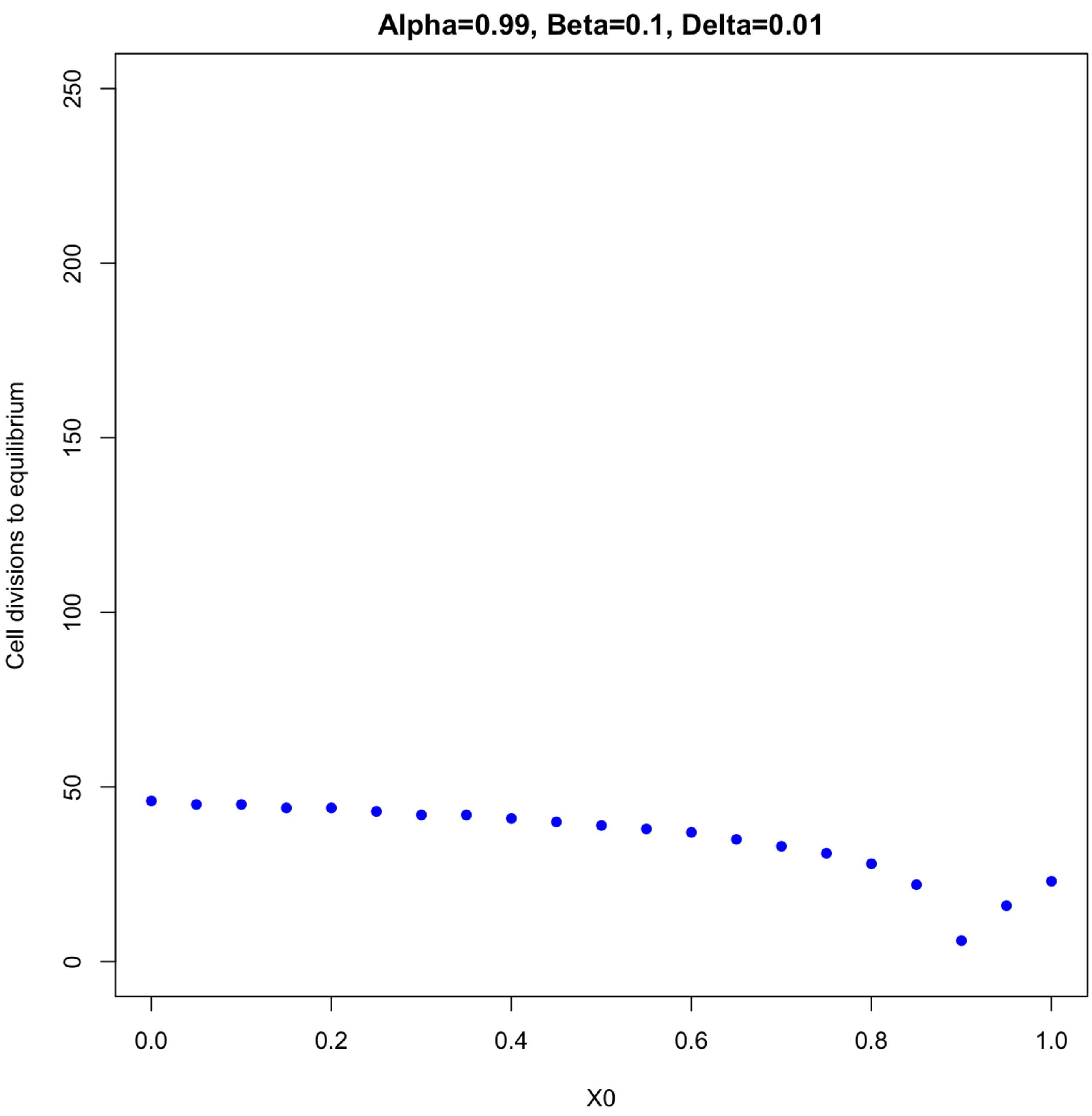

Hide

```
#grid.arrange(X0alphaplot,X0betaplot,X0deltaplot,ncol=2)
if(saveplots=="T"|saveplots=="TRUE"){
 dev.off()
}
```

Plot 16

This plot shows the final values of Y and Z that are possible for different values of Y.

Hide

```
if(saveplots=="T"|saveplots=="TRUE"){
 pdf(file=paste0("article1-output/",fileout,"plot16.pdf"),width=30,height=30,pointsiz
e=30)
}
XvsYZ<-subset(parresultsplots,parresultsplots$X0==1.0)
XvsYZ$X<-round(XvsYZ$X,digits=2)
XvsYZ$Y<-round(XvsYZ$Y,digits=2)
XvsYZ$Z<-round(XvsYZ$Z,digits=2)
plot(XvsYZ$X,XvsYZ$Y,type="p",pch=16,col=ifelse(XvsYZ$Y==max(XvsYZ$Y),"red","blue"),x
lim=c(0.0,1.0),ylim=c(0.0,1.0),xlab="Final X",ylab="Final Y")
```

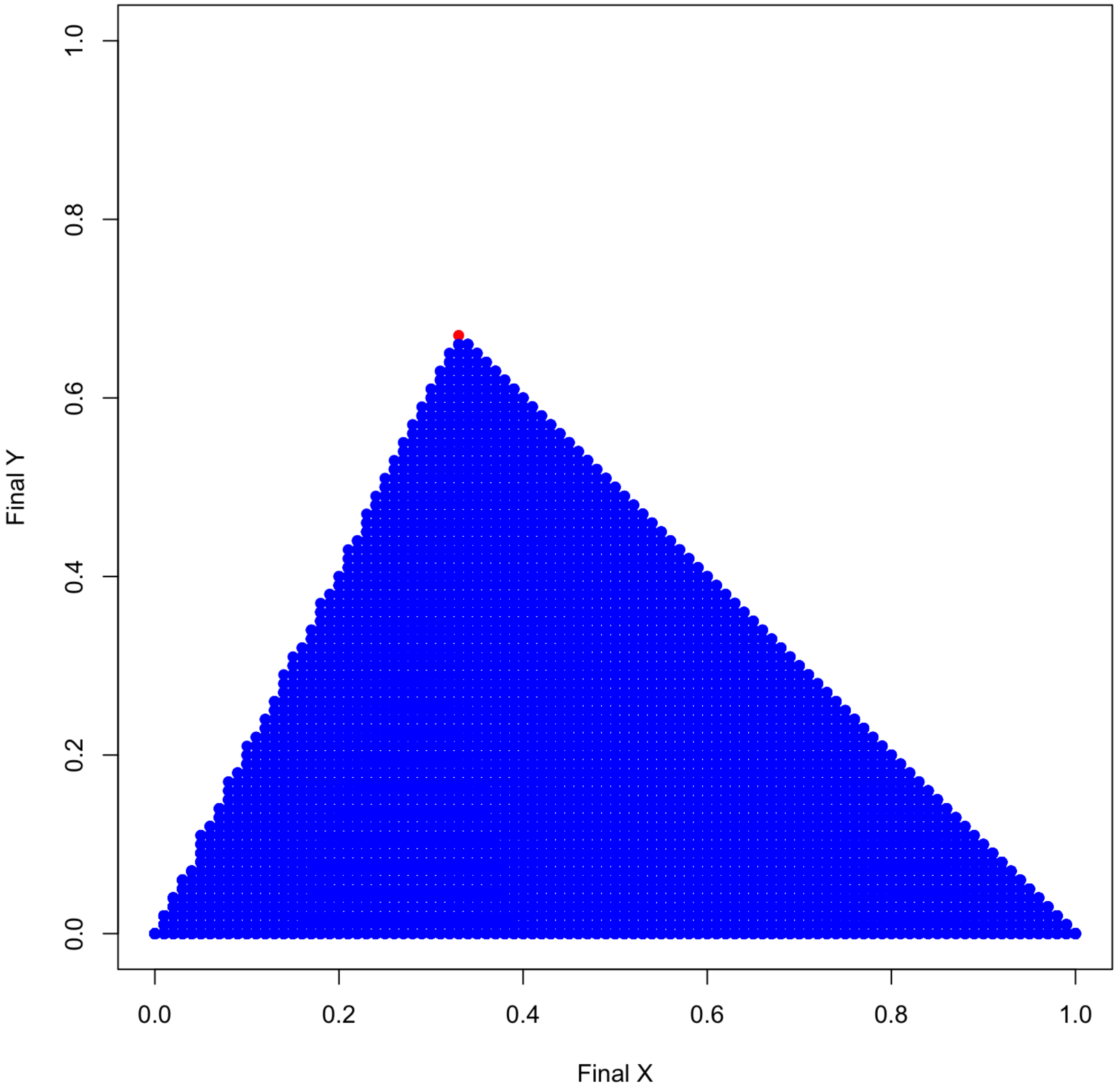

Hide

```
plot(XvsYZ$X,XvsYZ$Z,type="p",pch=16,col=ifelse(XvsYZ$Z==max(XvsYZ$Z),"red","blue"),x
lim=c(0.0,1.0),ylim=c(0.0,1.0),xlab="Final X",ylab="Final Z")
```

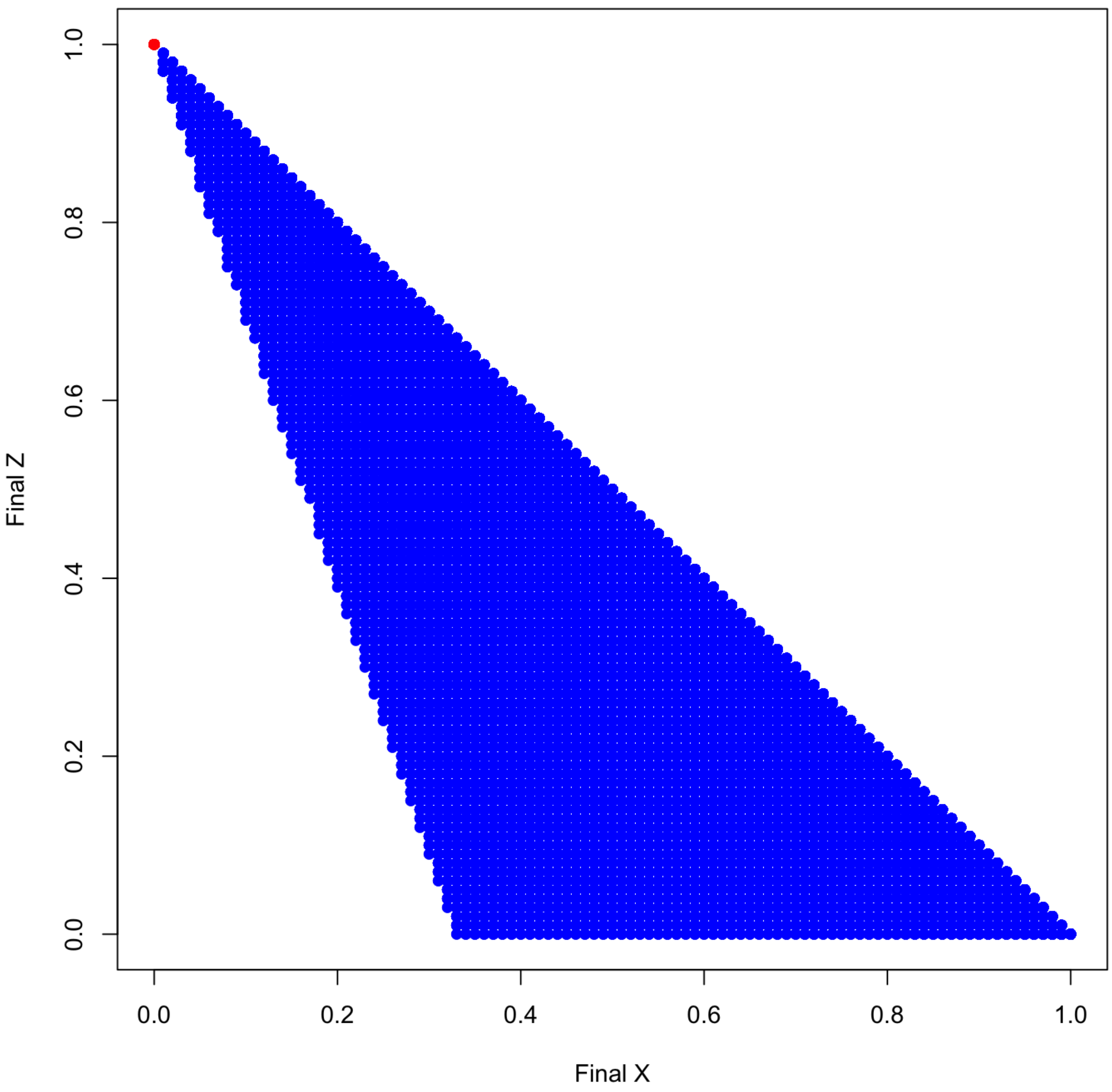

Hide

```
plot(XvsYZ$Y,XvsYZ$Z,type="p",pch=16,col=ifelse(XvsYZ$X==max(XvsYZ$X),"red","blue"),x
lim=c(0.0,1.0),ylim=c(0.0,1.0),xlab="Final Y",ylab="Final Z")
```

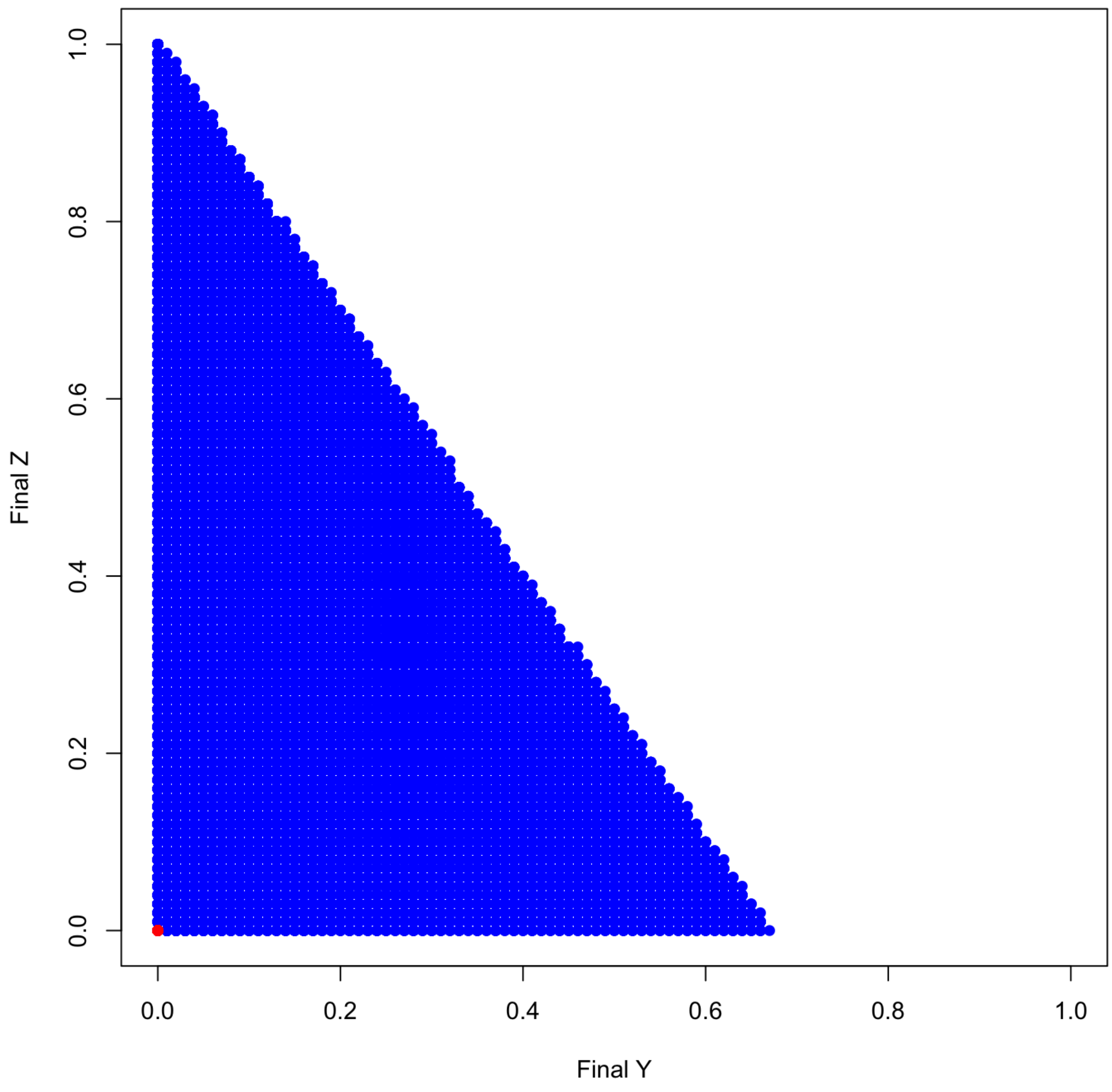

Hide

```
if(saveplots=="T"|saveplots=="TRUE"){
 dev.off()
}
```
